# Airway secretory cell fate conversion via YAP‐mTORC1‐dependent essential amino acid metabolism

**DOI:** 10.15252/embj.2021109365

**Published:** 2022-03-14

**Authors:** Hae Yon Jeon, Jinwook Choi, Lianne Kraaier, Young Hoon Kim, David Eisenbarth, Kijong Yi, Ju‐Gyeong Kang, Jin Woo Kim, Hyo Sup Shim, Joo‐Hyeon Lee, Dae‐Sik Lim

**Affiliations:** ^1^ Department of Biological Sciences National Creative Research Center for Cell Plasticity Korea Advanced Institute of Science and Technology (KAIST) Daejeon South Korea; ^2^ Jeffrey Cheah Biomedical Centre Wellcome – MRC Cambridge Stem Cell Institute University of Cambridge Cambridge UK; ^3^ Princess Máxima Center for Pediatric Oncology Utrecht The Netherlands; ^4^ Graduate School of Medical Science and Engineering Korea Advanced Institute of Science and Technology (KAIST) Daejeon South Korea; ^5^ GenomeInsight Inc. Daejeon South Korea; ^6^ Department of Pathology Yonsei University College of Medicine Seoul South Korea; ^7^ Department of Physiology Development and Neuroscience University of Cambridge Cambridge UK

**Keywords:** Damage‐Associated Transient Progenitors, essential amino acid metabolism, Hippo‐YAP signaling, mTORC1‐ATF4 axis, pulmonary fibrosis and bronchiolitis obliterans, Metabolism, Respiratory System, Signal Transduction

## Abstract

Tissue homeostasis requires lineage fidelity of stem cells. Dysregulation of cell fate specification and differentiation leads to various diseases, yet the cellular and molecular mechanisms governing these processes remain elusive. We demonstrate that YAP/TAZ activation reprograms airway secretory cells, which subsequently lose their cellular identity and acquire squamous alveolar type 1 (AT1) fate in the lung. This cell fate conversion is mediated via distinctive transitional cell states of damage‐associated transient progenitors (DATPs), recently shown to emerge during injury repair in mouse and human lungs. We further describe a YAP/TAZ signaling cascade to be integral for the fate conversion of secretory cells into AT1 fate, by modulating mTORC1/ATF4‐mediated amino acid metabolism *in vivo*. Importantly, we observed aberrant activation of the YAP/TAZ‐mTORC1‐ATF4 axis in the altered airway epithelium of bronchiolitis obliterans syndrome, including substantial emergence of DATPs and AT1 cells with severe pulmonary fibrosis. Genetic and pharmacologic inhibition of mTORC1 activity suppresses lineage alteration and subepithelial fibrosis driven by YAP/TAZ activation, proposing a potential therapeutic target for human fibrotic lung diseases.

## Graphical abstract

Hippo signaling‐dependent amino acid metabolism triggers cell‐type plasticity in the lung epithelium favoring inflammatory fibrosis.

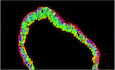

## Introduction

Epithelial integrity and homeostasis are of central importance to tissue maintenance and function. The alterations in epithelial identity and cellular state can induce lineage infidelity and eventually develop various diseases. However, the cellular and molecular mechanisms that reprogram the lineage identity and fate specification are little known.

The proliferation and differentiation of distinct stem and progenitor cells that reside alongside the pulmonary axis are vital to maintain the lung epithelium (Hogan *et al*, 2014). In the airways, secretory cells marked by the expression of *Scgb1a1*, which encodes the club cell secretory protein (CCSP, CC10), are key stem/progenitor cells that are highly prevalent in both human and mouse. They maintain the airway epithelium by self‐renewal and differentiation into ciliated cells during homeostasis and regeneration (Rawlins *et al*, 2009). Secretory cells in the proximal airways can also dedifferentiate into basal stem cells after genetic depletion of resident basal cells, whereas secretory cells in the distal airways can contribute to alveolar regeneration following alveolar injury, indicating the plasticity of airway secretory cells in context‐dependent manner (Rock *et al*, 2011b; Barkauskas *et al*, 2013; Zhao *et al*, 2014). Besides the functional role of secretory cells as stem and progenitor cells in the lung, they are critical for maintaining barrier integrity by involving in host defense and xenobiotic metabolism (Jones *et al*, 1983; Wang *et al*, 2003; Gamez *et al*, 2015). Importantly, several lines of evidence reveal that dysregulation of secretory cell maintenance is highly relevant to lung diseases such as bronchiolitis obliterans (BO) syndrome (Yanagi *et al*, 2015; Liu *et al*, 2019). Even though the regulation of secretory cells during homeostasis and injury repair has been established, the molecular program that influences the fate behavior of secretory cells remains unknown. In particular, how the reprograming of secretory cell identity impairs tissue integrity and causes lung dysfunction is poorly understood.

The Hippo signaling pathway, an evolutionally conserved kinase cascade, plays a key role in maintaining tissue homeostasis and function by regulating stem cell behaviors (Zhao *et al*, 2010). In the canonical Hippo pathway, upstream kinases MST1/2 phosphorylate LATS1/2 kinases, which then phosphorylate transcriptional coactivators of YAP/TAZ, causing their cytoplasmic localization and degradation, and inhibit their transcriptional activities (Zhao *et al*, 2010). Proper regulation of Hippo/YAP signaling is critical for proliferation and differentiation of stem and progenitor cells during lung development. Disruption of dynamic YAP/TAZ activity causes defects in airway morphogenesis, cell proliferation, and lineage differentiation in developing lungs (Mahoney *et al*, 2014; Lange *et al*, 2015; Lin *et al*, 2015; Nantie *et al*, 2018; van Soldt *et al*, 2019). In particular, dysregulation of Hippo/YAP signaling is suggested to impair lung maintenance and function, which is implicated in lung diseases such as pulmonary fibrosis (PF) (Gokey *et al*, 2018; LaCanna *et al*, 2019). Nevertheless, the molecular mechanisms of how YAP/TAZ signaling controls the fate behaviors of airway stem and progenitor cells in adult lungs and its implication in lung diseases remain largely unknown.

Here we show that YAP/TAZ signaling modulates the fate behaviors of airway secretory cells, which is implicated in human lung diseases. Single‐cell RNA sequencing (scRNA‐seq) analysis reveals that sustained activation of YAP/TAZ by LATS1/2 deletion reprograms secretory cells to lose their cellular identity and convert into squamous alveolar type 1 (AT1) cells, via the distinct transitional cell states of damage‐associated transient progenitors (DATPs) (Choi *et al*, 2020; Kobayashi *et al*, 2020; Strunz *et al*, 2020). We further demonstrate that mTORC1‐ATF4 activity mediated by YAP/TAZ activation realigns amino acid metabolism, integral to the transition of secretory cells into DATP‐AT1 fate. Importantly, aberrant activation of the YAP/TAZ‐mTORC1‐ATF4 axis is detected in the airways of human BO lungs concomitant with the loss of secretory cell identity and the emergence of DATPs and AT1‐like cells. Overall, our study identifies a core molecular program regulating the fate behaviors of secretory cells in lungs and provides new insights into how dysregulation of stem and progenitor cell fate impairs epithelium integrity and causes human lung diseases.

## Results

### Sustained activation of YAP/TAZ drives the differentiation of airway secretory cells into AT1 cells

**Figure 1:**
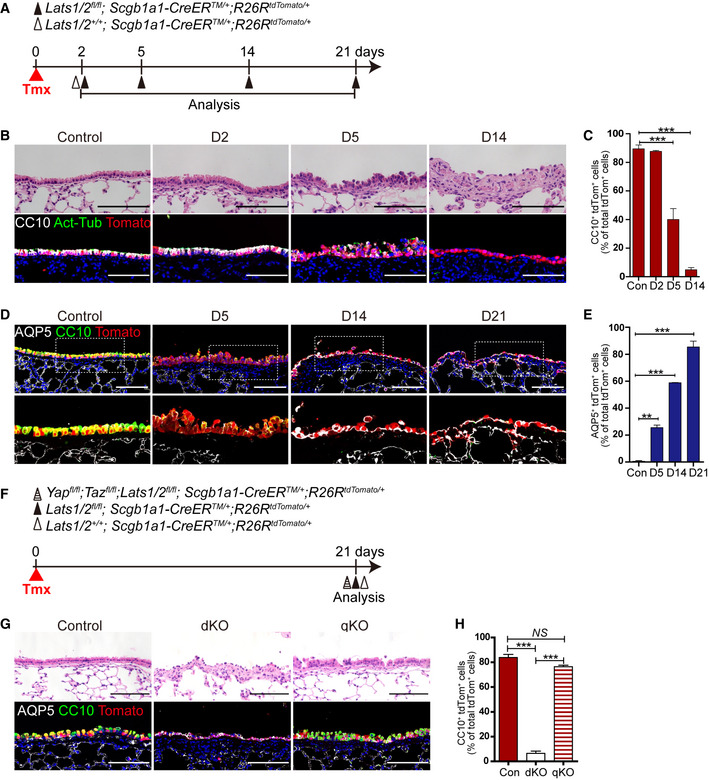
*Lats1*/*2* deletion induces the differentiation of secretory cells into AT1 cells in the airways Experimental design for lineage‐tracing analysis of secretory cells following genetic depletion of *Lats1* and *Lats2*. Specific time points for tamoxifen induction and tissue analysis are indicated.Representative H&E (top) and immunofluorescent (IF) images (bottom) of control and Lats1/2 dKO airways at indicated time points post tamoxifen treatment. Tomato (for Scgb1a1 lineage, red), CC10 (for secretory cell, white), acetylated tubulin (Act‐Tub, for ciliated cell, green), and DAPI (blue). Scale bar, 100 μm.Statistical quantification of *Scgb1a1* lineage‐labeled tdTomato^+^CC10^+^ secretory cells in (B). Data are presented as mean ± SEM (*n* = 5 mice for each group,). ***P < 0.001 (Student’s *t*‐test).Representative IF images of control and Lats1/2 dKO airways at indicated time points post tamoxifen treatment. Tomato (for Scgb1a1 lineage, red), CC10 (green), AQP5 (for AT1 cell, white), and DAPI (blue). Scale bar, 100 μm.Quantification of *Scgb1a1* lineage‐labeled tdTomato^+^AQP5^+^ AT1 cells in (D). Data are presented as mean ± SEM (*n* = 5 mice for each group). **P < 0.01, ***P < 0.001 (Student’s *t*‐test).Experimental design for lineage‐tracing analysis of secretory cells following genetic depletion of *Lats1*/*2* (double knockout, dKO) and *Lats1*/*2*;*Yap*;*Taz* (quadruple knockout, qKO). Specific time points for tamoxifen induction and tissue analysis are indicated.Representative H&E (top) and IF images (bottom) of control, dKO, and qKO airways at day 21 post tamoxifen treatment. Tomato (for Scgb1a1 lineage, red), CC10 (green), AQP5 (white), and DAPI (blue). Scale bar, 100 μm.Quantification of *Scgb1a1* lineage‐labeled tdTomato^+^CC10^+^ secretory cells in (G). Data are presented as mean ± SEM (*n* = 3 mice for each genotype). ***P < 0.001, NS, not significant (Student’s *t*‐test).

To investigate the regulatory role of YAP/TAZ signaling in the cellular identity of airway stem and progenitor cells, we established *Lats1^fl^* ^/^ *^fl^*;*Lats2^fl^* ^/^ *^fl^*;*Scgb1a1‐CreER^TM^* ^/+^;*R26R^tdTomato^* ^/+^ mice (hereafter Lats1/2 dKO) to delete both LATS1 and LATS2 genes in secretory cells (Fig 1A). Two days after tamoxifen induction, *Scgb1a1* ^+^ lineage‐labeled secretory cells of the control lungs (*Lats1* ^+/+^;*Lats2* ^+/+^;*Scgb1a1‐CreER^TM^* ^/+^;*R26R^tdTomato^* ^/+^) barely expressed YAP and TAZ (Appendix Fig S1A and B). By contrast, *Lats1*/*2*‐deficient secretory cells showed a significant increase of nuclear expression of YAP and TAZ (Appendix Fig S1A and B). Consistent with YAP/TAZ nuclear localization, *Lats1*/*2*‐deficient secretory cells significantly upregulated the expression levels of genes known to be regulated by YAP and TAZ (Appendix Fig S1C). Histological analysis revealed that at day 5 following YAP/TAZ activation, the pseudostratified airway epithelium was disrupted and begun to be stratified and flattened in Lats1/2 dKO lungs (Fig 1B). Remarkably, the expression of secretory cell marker CC10 was substantially decreased in *Lats1*/*2*‐deficient secretory cells, suggesting the loss of cellular identity by YAP/TAZ activation (Fig 1B and C). Furthermore, we also observed the subepithelial fibrosis with an increase in collagen deposition and proliferating mesenchymal cells following the alteration of airway epithelium in Lats1/2 dKO lungs, which is consistent with previous reports (Lee *et al*, 2016; McNeill & Reginensi, 2017) (Appendix Fig S1D).

**Figure EV1:**
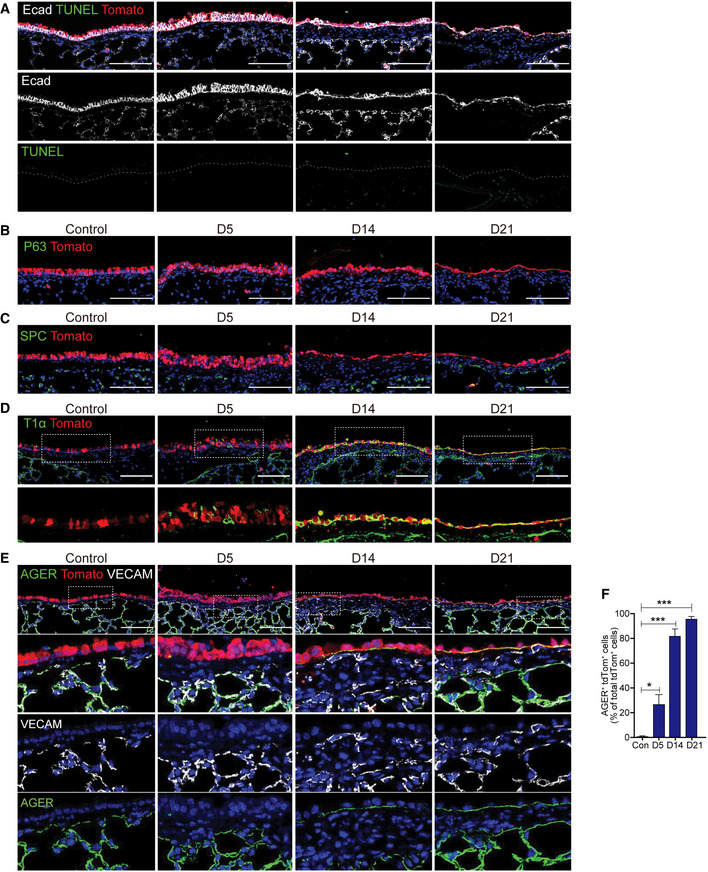
YAP/TAZ activation in secretory cells enforces fate conversion into AT1 cells, but bypasses basal or AT2 cells ARepresentative IF images showing the expression of epithelial cell marker E‐cadherin and apoptosis marker TUNEL in *Scgb1a1* lineage‐labeled tdTomato^+^ cells of control and Lats1/2 dKO lungs. Tomato (for secretory lineage, red), E‐cadherin (for epithelial cell, white), TUNEL (for apoptotic cells, green), and DAPI (blue). Scale bars, 100 μm.BRepresentative IF images showing the expression of basal cell marker P63 in *Scgb1a1* lineage‐labeled tdTomato^+^ cells in control and Lats1/2 dKO lungs. Tomato (for secretory lineage, red), P63 (green), and DAPI (blue). Scale bars, 100 μm.CRepresentative IF images showing the expression of AT2 cell marker SPC in *Scgb1a1* lineage‐labeled tdTomato^+^ cells in control and Lats1/2 dKO lungs. Tomato (for secretory lineage, red), SPC (green), and DAPI (blue). Scale bars, 100 μm.D, ERepresentative IF images showing the expression of AT1 cell markers T1α (D), AGER (E), and endothelial marker VECAM (E) in *Scgb1a1* lineage‐labeled tdTomato^+^ cells in control and Lats1/2 dKO lungs. Tomato (for secretory lineage, red), T1α (D, green), AGER (E, green), VECAM (E, white), and DAPI (blue). Scale bars, 100 μm.FQuantification of *Scgb1a1* lineage‐labeled tdTomato^+^AGER^+^ AT1 cells in (E). Data are presented as mean ± SEM (*n* = 5 mice for each group). *P < 0.05, ***P < 0.001 (Student’s *t*‐test).

Of interest, *Lats1*/*2*‐deficient secretory cells significantly lost their identity, but still maintained epithelial identity based on the expression of E‐cadherin with no sign of apoptosis (Fig EV1A). Thus, we next sought to further investigate the changes in cellular property of secretory cells driven by YAP/TAZ activation. Given the differentiation potential of secretory cells into basal or alveolar type 2 (AT2) cells (Rock *et al*, 2011b; Barkauskas *et al*, 2013; Zhao *et al*, 2014), we first checked the lineage conversion of *Scgb1a1* ^+^ lineage‐labeled secretory cells into these cell types in Lats1/2 dKO lungs. Immunofluorescent (IF) staining showed that neither p63^+^ basal nor SPC^+^ AT2 cells arose from *Lats1*/*2*‐deficient secretory cells (Fig EV1B and C). Lineage‐labeled airway epithelium in Lats1/2 dKO lungs displayed the squamous morphology (Figs 1B and EV1A–C). Therefore, we then examined the acquisition of AT1 cell fate. Surprisingly, the expression of AQP5, a mature marker for AT1 cells, emerged in *Scgb1a1* ^+^ lineage‐labeled cells at day 14 post tamoxifen treatment in Lats1/2 dKO airways (Fig 1D and E). By additional IF staining for other canonical AT1 markers, such as T1α and AGER, we further verified that lineage‐labeled cells in Lats1/2 dKO airways displayed positive for T1α and AGER (Fig EV1D–F). We also observed the microvascular structure expressing endothelial marker VECAM adjacent to lineage‐labeled AT1 cells, suggesting that the secretory cells are converted into bona fide AT1 cells retaining the potential for gas exchange with capillary endothelial cells (Fig EV1F). Significantly, deletion of *Yap* and *Taz* in Lats1/2 dKO lungs, by establishing *Yap^fl^* ^/^ *^fl^*;*Taz^fl^* ^/^ *^fl^*;*Lats1^fl^* ^/^ *^fl^*;*Lats2^fl^* ^/^ *^fl^*;*Scgb1a1‐CreER^TM^* ^/+^;*R26R^tdTomato^* ^/+^ mice (hereafter quadruple knockout mice, or qKO mice), completely rescued the alterations of airway epithelial lineage fate including the restoration of secretory cell identity with CC10 expression (Fig 1F–H). Taken together, we conclude that sustained activation of YAP/TAZ by *Lats1*/*2* deletion drives the lineage conversion of secretory cells into squamous AT1 cell fate.

### Sustained activation of YAP/TAZ reprograms airway secretory cells to enter DATP cell states leading to AT1 cell fate conversion

**Figure 2:**
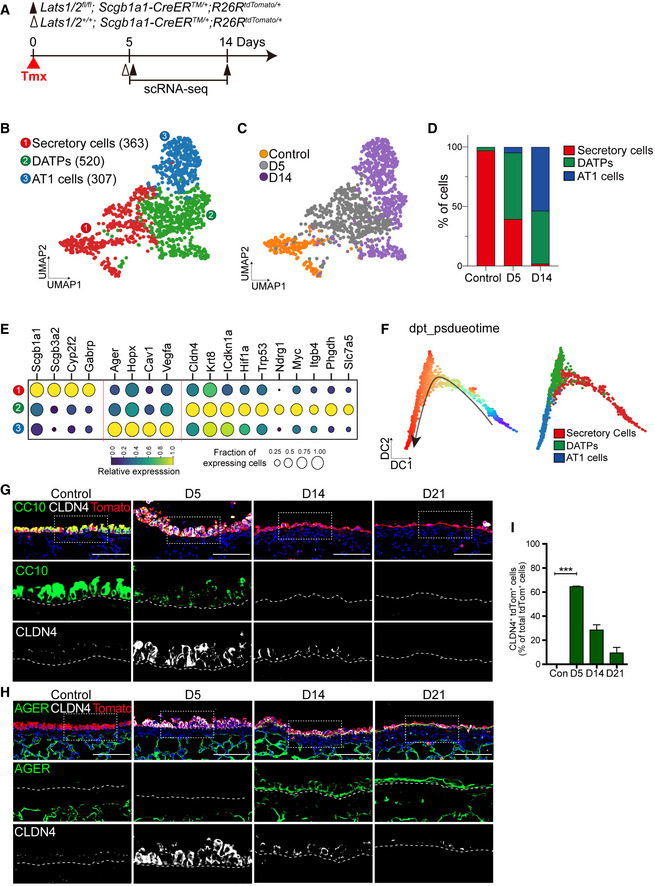
Sustained activation of YAP/TAZ drives the fate conversion of secretory cells into AT1 cells via DATP cell states AExperimental design for isolating *Scgb1a1* lineage‐labeled cells from control and Lats1/2 dKO lungs at indicated time points post tamoxifen treatment. Isolated cells were subjected to scRNA‐seq analysis.BClusters of *Scgb1a1* lineage‐labeled cells from 10XGenomics 3’ single‐cell RNA sequencing (scRNA‐seq) analysis visualized by Uniform Manifold Approximation and Projection (UMAP), assigned by specific colors at day 5 and 14 post tamoxifen treatment. The number of cells in the individual cluster is depicted in the figure.CDistribution of each cluster across indicated time points after tamoxifen treatment.DBar graph showing the percentage of secretory, DATPs, and AT1 cell clusters in control and Lats1/2 dKO lungs at day 5 and 14 post tamoxifen treatment.EBlob plot depicting selected marker genes in cell clusters. Dot size encodes the percentage of cells expressing the gene, and color encodes the average per cell gene expression level.FPseudotime ordering of *Scgb1a1* lineage‐labeled cells colored by cluster assignment (right) according to diffusion pseudotime (DPT, left) order.G, HRepresentative IF images of control and Lats1/2 dKO airways at indicated time points post tamoxifen treatment. Tomato (for Scgb1a1 lineage, red), CLDN4 (for DATPs, white), CC10 (G, green), AGER (H, for AT1 cell, green), and DAPI (blue). Scale bar, 100 μm.IQuantification of *Scgb1a1* lineage‐labeled tdTomato^+^CLDN4^+^ DATPs in (G and H). Data are presented as mean ± SEM (*n* = 5 mice for each genotype). ***P < 0.001 (Student’s *t*‐test).

We next attempted to define differentiation programs mediating the transition of secretory cells to AT1 cell fate by YAP/TAZ activation. To do this, we performed scRNA‐seq analysis of freshly isolated *Scgb1a1* ^+^ lineage‐labeled cells (CD45^−^CD31^−^EpCAM^+^Tomato^+^) from control and Lats1/2 dKO lungs post tamoxifen treatment (Fig 2A and Appendix Fig S2A). To focus on the trajectory and molecular identity of secretory to AT1 fate conversion, we further analyzed lineage‐labeled epithelial cells of control and Lats1/2 dKO lungs across different time points after excluding non‐epithelial populations and ciliated cells (Appendix Fig S2B and C; see Method section). Based on the expression of canonical markers for secretory and AT1 cells, we identified three distinct cell populations from the scRNA‐seq analysis (Fig 2B and C). Distribution of each cluster across the time points allowed us to assess how YAP/TAZ activation modulates the loss of secretory cell identity and further differentiation into AT1 cells (Fig 2D). As expected, most lineage‐labeled cells in control lungs were secretory cells based on the expression of secretory cell markers such as *Scgb1a1* and *Scgb3a2* (Fig 2D and E). However, *Lats1*/*2*‐deficient secretory cells showed much lower expression levels of most secretory cell markers such as *Cyp2f2* and *Scgb3a2* (Appendix Fig S2D). Yet, they still express a comparable level of *Scgb1a1*, which assigns them as “secretory cell” at day 5 post tamoxifen treatment (Appendix Fig S2D). Consistent with our IF staining results (Figs 1D and EV1D and E), scRNA‐seq analysis revealed that AT1 cells emerged with a substantial reduction of secretory cells at day 14 post tamoxifen treatment (Fig 2B–E). Significantly, we also identified a population showing distinct transcriptional signatures that emerged at day 5 and persisted until day 14 (Fig 2B–E). This population expressed neither secretory nor AT1 cell markers. Instead, they were marked by enriched expressions of *Cldn4*, *Krt8*, or *Ndrg1*, which are known as the markers for the transitional cell states of DATPs emerged during alveolar regeneration (Choi *et al*, 2020; Kobayashi *et al*, 2020; Strunz *et al*, 2020) (Fig 2E). Pseudotime analysis suggested that AT1 cell differentiation from secretory cells is mediated by DATPs (Fig 2F). To further validate the emergence of DATPs from lineage‐labeled secretory cells upon YAP/TAZ activation, we performed IF staining for a DATP marker, CLDN4, in Lats1/2 dKO airways. Consistent with our scRNA‐seq analysis, CLDN4^+^ DATPs were detected in lineage‐labeled cells at day 5 following tamoxifen treatment (Fig 2G–I). The frequency of DATPs was reduced with the differentiation of AT1 cells at day 14 (Fig 2H and I). We observed no detectable levels of CLDN4 expression in control airways (Fig 2G–I). Collectively, these data suggest that activation of YAP/TAZ reprograms secretory cells to lose their cellular identity and acquires AT1 cell fate through DATP cell states that are known to be induced during lung regeneration.

### Uptake of essential amino acids mediated by YAP/TAZ activation is integral to the transition of secretory cells to DATP‐AT1 fate

**Figure EV2:**
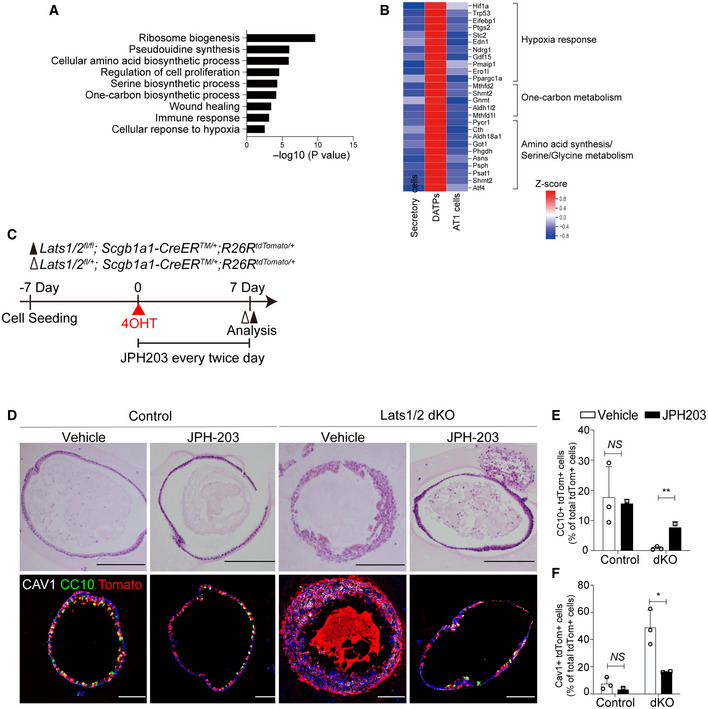
YAP/TAZ signaling modulates the uptake of essential amino acids that are crucial for cell fate conversion of secretory cells AAnalysis of Gene Ontology (GO) terms in DATP cell states driven by YAP/TAZ activation.BHeatmap of secretory, DATPs, and AT1 cells for metabolism‐related genes including hypoxia response, one‐carbon metabolism, and amino acid synthesis/serine/glycine metabolism.CExperimental designs for organoid cultures derived from secretory cells isolated from unlabeled control and Lats1/2 dKO lungs. Isolated secretory cells were seeded in 3D organoid cocultures with stromal cells. At day 7 post seeding, 4‐hydroxytamoxifen (4‐OHT) was treated, followed by the addition of SLC7A5/LAT1 inhibitor (JPH203) twice every day for 7 days. Organoids were analyzed at day 7 post 4‐OHT treatment.DRepresentative H&E and IF images of 3D organoids in (C). Tomato (red), CC10 (green), CAV1 (for AT1 cells, white), and DAPI (blue). Scale bars, 100 μm.E, FQuantification of *Scgb1a1* lineage‐labeled tdTomato^+^CC10^+^ secretory (E) and CAV1^+^ AT1 (F) cells in 3D organoids in (D). Data are presented as mean ± SEM (*n* = at least 2 technical replicates per group). *P < 0.05, **P < 0.01, NS, not significant (Student’s *t*‐test).

**Figure 3:**
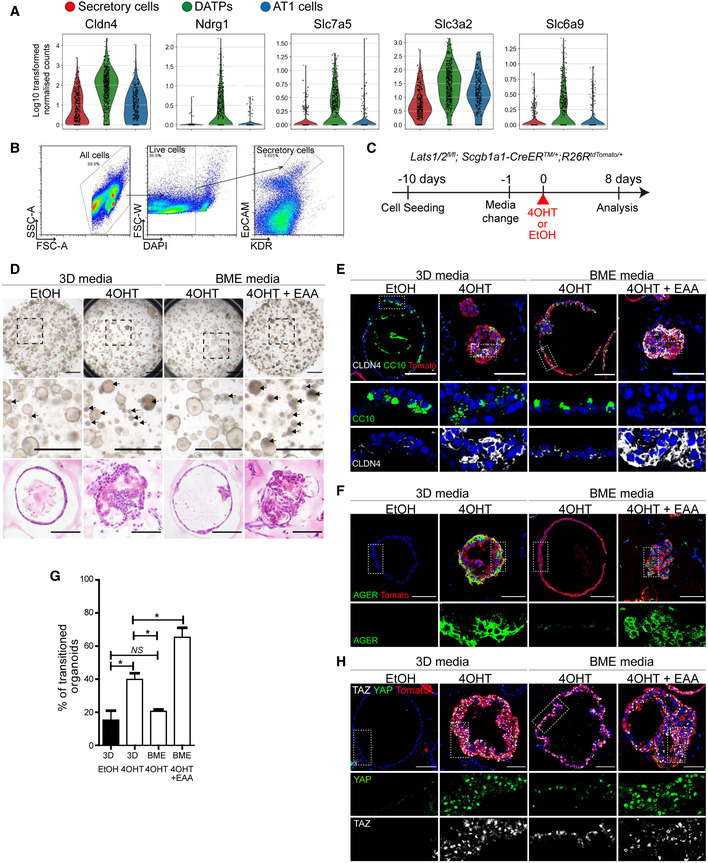
The uptake of essential amino acids (EAAs) is required for cell fate conversion of secretory cells mediated by YAP/TAZ activation AViolin plots of DATP markers (*Cldn4, Ndrg1*) and EAA transporters (*Slc7a5, Slc3a2, Slc6a9*) in control and Lats1/2 dKO lung cells at day 5 post tamoxifen treatment.B, CFlow cytometry sorting scheme for isolation of secretory cells (EpCAM^+^KDR^+^) (B) and experimental designs for organoid cultures (C). Secretory cells were isolated from unlabeled Lats1/2 dKO lungs, followed by 3D organoid cocultures with stromal cells. 3D normal media was changed to experimental media 1 day prior to 4‐hydroxytamoxifen (4‐OHT) or ethanol (EtOH) treatment. Organoids were analyzed at day 8 post 4‐OHT or EtOH treatment.DRepresentative brightfield and H&E images of 3D organoids. Arrow indicates transitioned 3D organoids retaining DATPs and AT1 cells. Scale bars, 1 mm for brightfield images, 200 μm for H&E images.E, FRepresentative IF images of secretory, DATPs, or AT1 cells in secretory organoids in indicated culture conditions. Tomato (for Scgb1a1 lineage, red), CC10 (E, green), CLDN4 (E, white), AGER (F, green), and DAPI (blue). Scale bars, 100 μm.GQuantification of transitioned 3D organoids in 3D normal media (3D), amino acid‐limited Basal MEM media (BME), or affluent repletion of EAA in BME media (BME+EAA) with 4‐OHT or EtOH treatment. Data are presented as mean ± SEM (*n* = 3 technical replicates per group). *P < 0.05, NS, not significant (Student’s *t*‐test).HRepresentative IF images of YAP and TAZ expression of secretory organoids in indicated culture conditions. Tomato (for Scgb1a1 lineage, red), YAP (green), TAZ (white), and DAPI (blue). Scale bars, 100 μm.

We next sought to determine the molecular programs, driven by YAP/TAZ activation, responsible for modulating the fate conversion of secretory cells. We found that transcriptional signatures of DATPs showed marked increases in the genes regulating metabolic process, especially in the amino acid synthesis (Fig EV2A and B). Importantly, DATPs showed much higher expression of solute carrier transporters such as *Slc7a5*/*Lat1* and *Slc3a2*, which form a heterodimeric complex and uptake essential amino acid (EAA) from the extracellular region (Yanagida *et al*, 2001; Dejure *et al*, 2020) (Fig 3A). Previous studies revealed that subsets of EAA transporters, such as *Slc7a5*/*Lat1*, are directly regulated by YAP/TAZ (Hansen *et al*, 2015; Park *et al*, 2016). Therefore, we aimed to evaluate the functional role of EAAs in directing the fate conversion of secretory cells into DATP‐AT1 cells using an *ex vivo* three‐dimensional (3D) organoid culture system. We recently identified KDR/FLK1 as the surface marker of airway secretory cells (Choi *et al*, 2021; Jiang *et al*, 2021). Using this marker, we isolated secretory cells (KDR^+^EpCAM^+^) from Lats1/2 dKO lungs, followed by organoid coculture with stromal cells (Fig 3B). At day 9 after cell seeding, we replaced culture medium with medium supplemented with AA‐limited basal medium (BME) or EAA‐enriched medium (BME+EAA) (Fig 3C). Ethanol (EtOH) or 4‐hydroxytamoxifen (4‐OHT) was added for control or *Lats1*/*2* deletion, respectively, at the following day post medium replacement (Fig 3C). As expected, control secretory cells formed normal cystic organoids (Fig 3D). By contrast, organoids derived from 4‐OHT‐treated secretory cells showed marked morphological changes with dense and folded structures (Fig 3D). Significantly, these organoids contained CLDN4^+^ DATPs and AGER^+^ AT1 cells, whereas control cystic organoids mainly retained CC10^+^ secretory cells (Fig 3D–G). Thus, we named folded organoids as “transitioned organoids.” Notably, amino acid depletion in the 3D medium dramatically restored these alterations, including the maintenance of secretory cell identity and reduction of DATP and AT1 cell conversion, driven by YAP/TAZ activation (Fig 3D–G). However, the addition of EAA in organoids derived from *Lats1*/*2*‐deficient secretory cells enhanced the generation of transitioned organoids retaining DATPs and AT1 cells (Fig 3D–G). We also verified increased nuclear expression of YAP and TAZ by 4‐OHT treatment in organoids with 3D and BME medium (Fig 3H). We then further asked whether a solute carrier transporter SLC7A5/LAT1, modulated by YAP/TAZ signaling, is a crucial regulator for secretory cell conversion by mediating EAA uptake. To do this, we treated JPH‐203, a potent inhibitor for SLC7A5/LAT1, in secretory organoids derived from control and Lats1/2 dKO lungs (Oda *et al*, 2010) (Fig EV2C). Notably, pharmacological inhibition of SLC7A5/LAT1 substantially rescued the loss of secretory cell identity and their conversion into AT1 cell fate mediated by YAP/TAZ activation (Fig EV2D–F). Taken together, these results indicate that fate conversion of secretory cells into DATPs and AT1 cells by YAP/TAZ activation requires SLC7A5/LAT1‐mediated EAA uptake.

### mTORC1‐ATF4 activity mediated by YAP/TAZ activation regulates amino acid uptake

**Figure 4:**
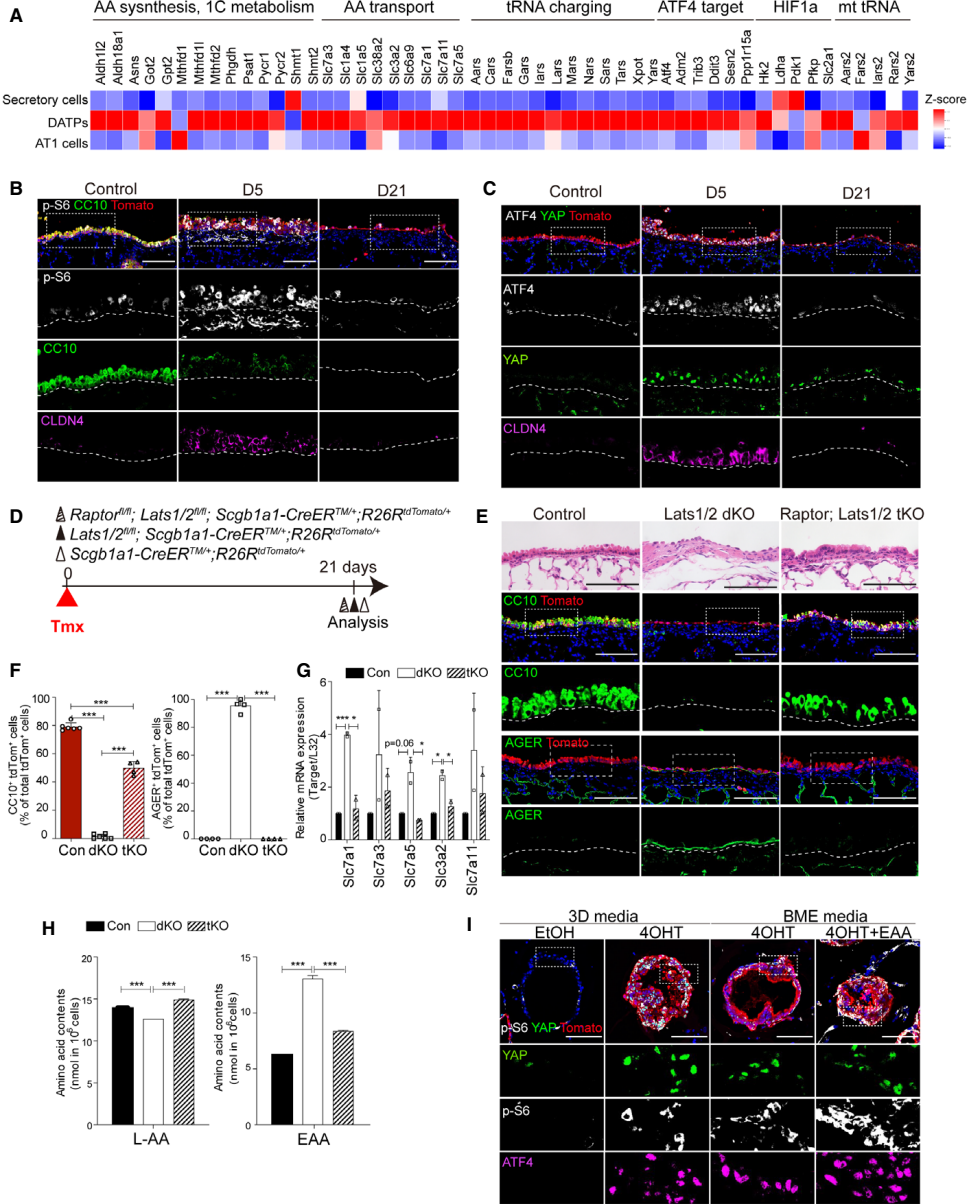
mTORC1‐ATF4 activity regulates the amino acid uptake for fate conversion of secretory cells Heatmap of ATF4‐dependent mTORC1 targets (AA synthesis, 1C metabolism, AA transport, tRNA charging), ATF4 targets, and ATF4‐independent mTORC1 targets (HIF1a, mt tRNA).Representative IF images showing the expression of secretory cell marker CC10, mTORC1 activity p‐S6, and DATP marker CLDN4 in *Scgb1a1* lineage‐labeled toTomato^+^ cells from serial sections of control and Lats1/2 dKO lung tissues. Tomato (for Scgb1a1 lineage, red), CC10 (green), p‐S6 (white), CLDN4 (magenta), and DAPI (blue). Scale bars, 100 μm.Representative IF images showing the nuclear expression of YAP and ATF4 in *Scgb1a1* lineage‐labeled tdTomato^+^ cells from serial sections of control and Lats1/2 dKO lung tissues. Of note, CLDN4 expression in lineage‐labeled cells having the nuclear YAP and TAZ. Tomato (for Scgb1a1 lineage, red), YAP (green), ATF4 (white), CLDN4 (magenta) and DAPI (blue). Scale bars, 100 μm.Experimental designs for lineage‐tracing of control, *Lats1*/*2* double knockout (dKO), and *Raptor*;*Lats1*/*2* homozygous triple knockout (tKO) lungs. Specific time points for tamoxifen treatment and analysis are indicated.Representative H&E and IF images of control, Lats1/2 dKO, and Raptor;Lats1/2 tKO airways at day 21 post tamoxifen treatment. Tomato (for Scgb1a1 lineage, red), CC10 (green, upper), AGER (green, lower), and DAPI (blue). Scale bar, 100 μm.Quantification of *Scgb1a1* lineage‐labeled tdTomato^+^CC10^+^ secretory cells (left) and tdTomato^+^AGER^+^ AT1 cells (right) in (E). Data are presented as mean ± SEM (*n* = 2–3 mice for each genotype). ***P < 0.001 (Student’s *t*‐test).Quantitative PCR (qPCR) analysis of ATF4‐dependent amino acid transporters (*Slc7a1, Slc7a3, Slc7a5, Slc34a2, Slc7a11*), on freshly isolated *Scgb1a1* lineage‐labeled cells from control and Lats1/2 dKO lungs at day 2 post tamoxifen treatment. Data are presented as mean ± SEM (*n* = 2 mice for each group). *P < 0.05, ***P < 0.0001 (Student’s *t*‐test).Measurement of L‐amino acid or EAAs (leucine, isoleucine, valine) contents in *Scgb1a1* lineage‐labeled tdTomato^+^ organoids derived from control, Lats1/2 dKO or Raptor;Lats1/2 tKO mice. Data are presented as mean ± SEM (*n* = 3 technical replicates for each genotype). *P < 0.05, **P < 0.001 (Student’s *t*‐test).Representative IF images showing the expression of YAP, p‐S6, and ATF4 in serial sections of 3D organoids derived from secretory cells in indicated culture conditions. Tomato (for Scgb1a1 lineage, red), YAP (green), p‐S6 (white), ATF4 (magenta), and DAPI (blue). Scale bars, 100 μm. Source data are available online for this figure.

The Gene Ontology (GO) enrichment analysis of our scRNA‐seq data showed that transcriptional signatures of DATPs retained not only “AA transporter” genes but also a wide range of mTORC1‐related targets, including “AA synthesis,” “1C metabolism,” and “tRNA charging” genes (Fig 4A). Interestingly, we also found that DATPs showed upregulated expression levels of the ATF4 transcript and its downstream target genes (Fig 4A). Recent studies have suggested a functional interaction between mTORC1 and ATF4 signaling, and Slc7a5/Lat1 was identified as its potent target gene (Adams, 2007; Ben‐Sahra *et al*, 2016; Torrence *et al*, 2021). Therefore, we hypothesized that upregulation of an mTORC1‐ATF4 response in DATPs, induced by YAP/TAZ activation, controls EAA uptake, which is crucial for fate conversion of secretory cells into AT1 cells. To test our hypothesis, we first checked whether mTORC1 signaling is activated in Lats1/2 dKO airways by assessing phosphorylated S6 ribosomal protein (p‐S6), the best‐characterized marker for mTORC1 signaling activation. We detected elevated expression of p‐S6 in *Scgb1a1* ^+^ lineage‐labeled cells at day 5 post tamoxifen treatment, which coincides with lineage‐labeled dKO secretory cells converting to CLDN4^+^ DATPs (Fig 4B). Elevated levels of p‐S6 expression reduced by day 21 when secretory cells were completely converted into AT1 fate, and DATPs were no longer present (Fig 4B). This suggests that YAP/TAZ‐mediated mTORC1 activation is transient with the emergence of DATPs during cell fate conversion. Furthermore, consistent with the p‐S6 expression pattern and scRNA‐seq analysis, we also observed increased expression of ATF4 in *Scgb1a1* ^+^ lineage‐labeled cells at day 5 following *Lats1*/*2* deletion, suggesting the activation of an mTORC1‐ATF4 axis in DATPs induced by YAP/TAZ activation (Fig 4C).

**Figure EV3:**
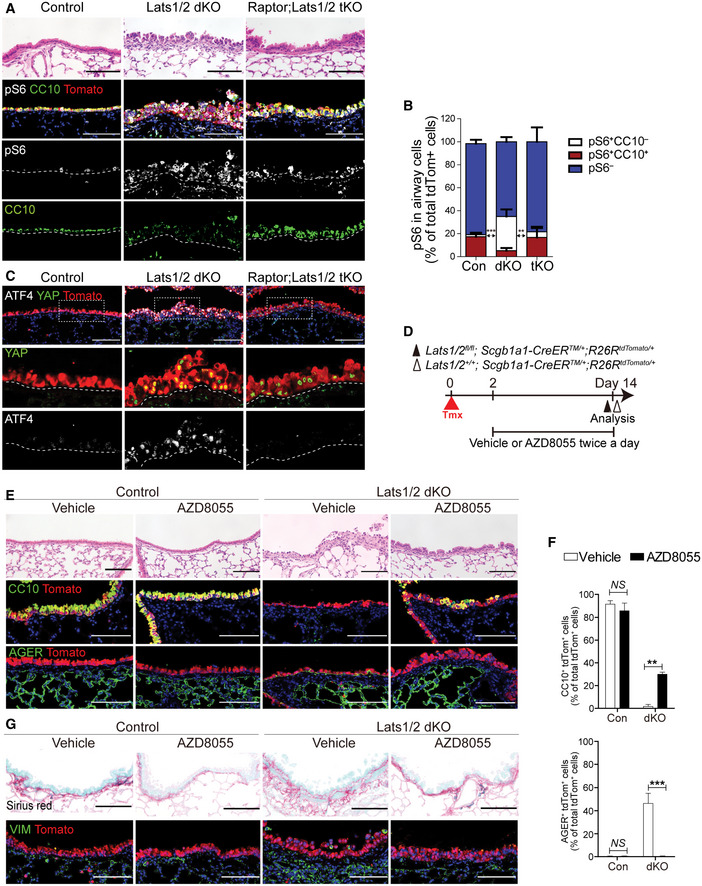
Ablation of *Raptor* suppresses YAP/TAZ‐induced mTORC1 and ATF4 activation A, BRepresentative H&E and IF images (A), and quantification (B) of secretory cells and mTORC1 activity in control, Lats1/2 dKO (dKO), and Raptor;Lats1/2 triple knockout (tKO) airways at day 5 post tamoxifen treatment. Tomato (A, for Scgb1a1 lineage, red), CC10 (A, green), pS6 (A, white), and DAPI (blue). Scale bars, 100 μm. Data are presented as mean ± SEM (*n* = 3 mice for each genotype). **P < 0.01, ***P < 0.0001 for pS6^+^CC10^−^ cells (Student’s *t*‐test).CRepresentative IF images showing the expression of nuclear YAP and ATF4 in *Scgb1a1* ^+^ lineage‐labeled cells of control, Lats1/2 dKO, and Raptor;Lats1/2 tKO airways at day 5 post tamoxifen treatment. Tomato (for Scgb1a1 lineage, red), YAP (green), ATF4 (white), and DAPI (blue). Scale bars, 100 μm.DExperimental designs for treating mTOR inhibitor (AZD8055) or vehicle to control and Lats1/2 dKO mice twice a day at indicated time points post tamoxifen treatment.ERepresentative H&E and IF images of control and Lats1/2 dKO lungs with vehicle or AZD8055 treatment at day 14 post tamoxifen treatment. Tomato (for secretory lineage, red), CC10 (middle, green), AGER (bottom, green), and DAPI (blue). Scale bars, 100 μm.FQuantification of *Scgb1a1* lineage‐labeled tdTomato^+^CC10^+^ secretory (top) and AGER^+^ AT1 cells (bottom) in (E). Data are presented as mean ± SEM (*n* = 3 mice for each group). **P < 0.005, ***P < 0.001 (Student’s *t*‐test).GRepresentative Sirius red staining for collagen (top) and IF images showing the expression of mesenchymal marker Vimentin (bottom) in control and Lats1/2 dKO lungs with vehicle or AZD8055 treatment. Tomato (for secretory lineage, red), vimentin (VIM, green), and DAPI (blue). Scale bars, 100 μm. Source data are available online for this figure.

We next sought to validate whether the activation of mTORC1 signaling causes fate conversion of secretory cells, mediated by YAP/TAZ activation. To address this question, we ablated Raptor, a core regulator for mTORC1 kinase in Lats1/2 dKO lungs, by establishing *Raptor^fl^* ^/^ *^fl^*;*Lats1^fl^* ^/^ *^fl^*;*Lats2^fl^* ^/^ *^fl^*;*Scgb1a1‐CreER^TM^* ^/+^;*R26R^tdTomato^* ^/+^ mice (hereafter triple knockout mice, or tKO mice) (Fig 4D). Deletion of *Raptor* significantly impaired mTORC1 activity based on the expression of p‐S6 in lineage‐labeled cells (Fig EV3A and B). We also observed a significant reduction of ATF4 expression in the cells from tKO mice, despite maintenance of strong nuclear expression of YAP (Fig EV3C). Importantly, ablation of mTORC1 activity by deletion of *Raptor* substantially rescued the loss of secretory cell identity driven by YAP/TAZ activation (Fig 4E and F). Further, *Raptor* deletion also blocked the fate conversion of secretory cells into AT1 cells (Fig 4E and F). We also verified that the expression of EAA transporters, such as *Slc7a5 and Slc3a2*, was augmented by YAP/TAZ activation in secretory cells (Fig 4G). However, disruption of mTORC1 activity significantly decreased their expressions in tKO secretory cells (Fig 4G). Consistently, we confirmed the enhanced uptake of EAA in Lats1/2‐deficient secretory organoids where secretory cells extensively transited into DATPs and AT1 fate (Fig 4H). Further, increased EAA uptake by YAP/TAZ activation was inhibited in tKO secretory organoids, suggesting the direct effect of mTORC1 activity on EAA uptake (Fig 4H). Pharmacological inhibition of mTOR signaling by treatment of AZD8055, a potent mTOR inhibitor, also suppressed the alterations of secretory cell fate by maintaining columnar airway epithelium retaining CC10 expression but lacking AGER expression (Fig EV3D–F). Notably, we also found that AZD8055 treatment alleviated the accumulation of peribronchiolar collagen and mesenchymal cells (Fig EV3G).

We further assessed the increased mTORC1‐ATF4 activity in *Lats1*/*2*‐deficient secretory organoids. The elevation of p‐S6 level and ATF4 expression coincided with the activation of YAP/TAZ in organoids treated with 4‐OHT in 3D medium (Fig 4I). Significantly, depletion of EAA retained the activity of mTORC1‐ATF4, yet blocked the transition of secretory cells into DATP‐AT1 cell fate in organoids with AA‐limited BME media (Fig 4I). These results indicate that EAA uptake via mTORC1‐ATF4 activity is determinant for the transition of secretory cells into DATPs and AT1 cells.

**Figure 5:**
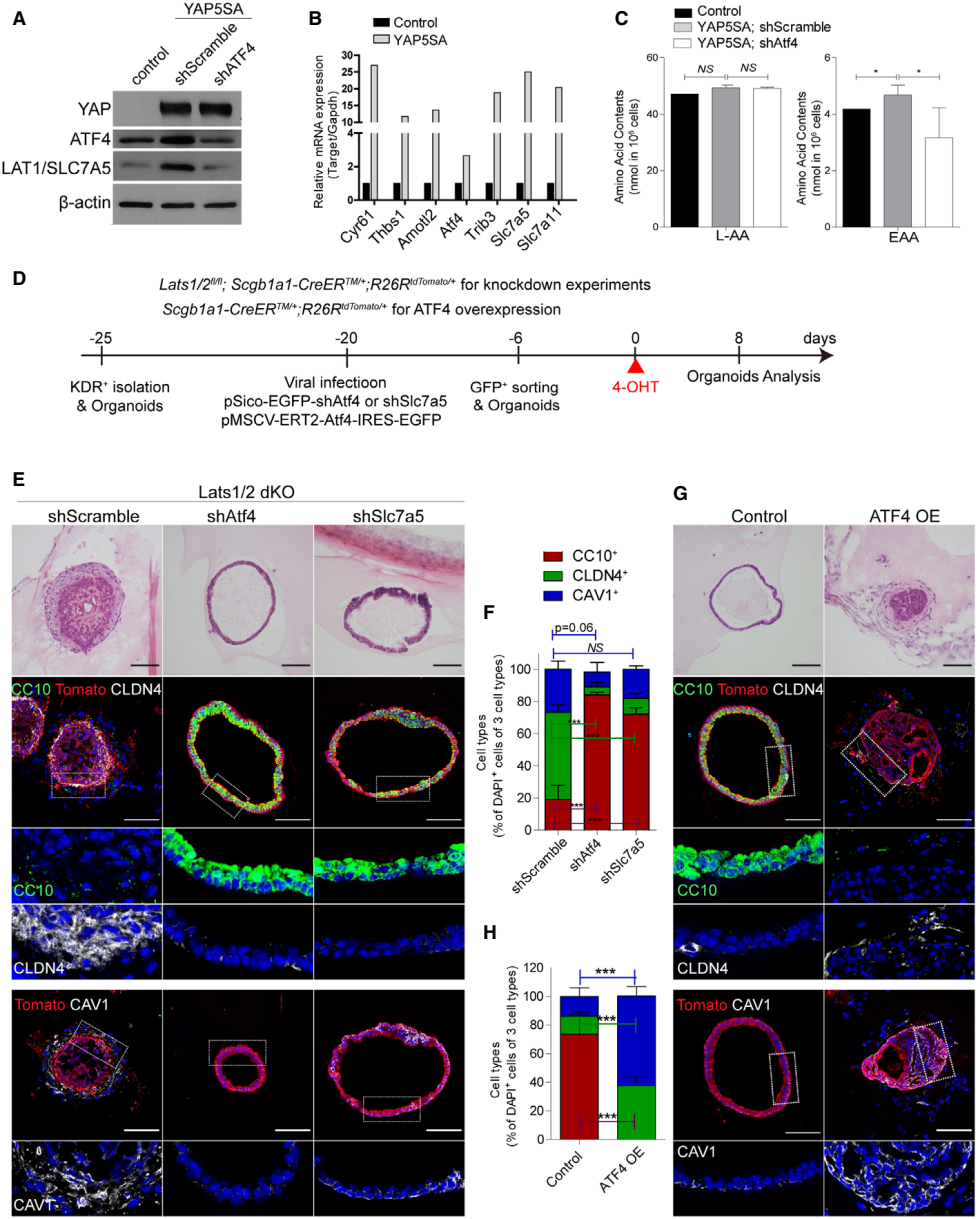
ATF4‐dependent essential amino acid uptake is crucial for the cell fate decision of secretory cells Western blot images for the expression of YAP, ATF4, and LAT1/Slc7a5 in control or YAP5SA overexpressed C22 airway epithelial cell line. Knockdown (KD) of ATF4 was induced by using lentiviral‐mediated inducible scramble shRNA (shScramble, control) or shATF4.Quantitative PCR (qPCR) analysis of YAP/TAZ targets (*Cyr61*, *Thbs1*, *Amotl2*), *ATF4* and the targets of ATF4 (*Trib3*, *Slc7a5*, *Slc7a11*).Concentration of L‐amino acids (L‐AA, left) or EAA (right; leucine, isoleucine, valine) in cell lines in (A). Data are presented as mean ± SEM (*n* = 3 biological replicates for each group). *P < 0.05, NS, not significant (Student’s *t*‐test).Experimental designs for culture and viral transduction for KD constructs (shRNA vectors) targeting Atf4 or Slc7a5 and ectopic expression of Atf4 in 3D secretory organoids.Representative H&E images and IF images showing the expression of secretory cell marker CC10, DATP marker CLDN4, and AT1 cell marker CAV1 in organoids derived from Lats1/2 dKO secretory cells with shscramble, shAtf4, or shSlc7a5. CC10 (green), CLDN4 (white), CAV1 (white) and DAPI (blue). Scale bars, 100 μm.Quantification of *Scgb1a1* ^+^ lineage‐labeled tdTomato^+^CC10^+^ secretory cells (red), tdTomato^+^CLDN4^+^ DATPs (green), and tdTomato^+^CAV1^+^ AT1 cells (blue) in (E). Data are presented as mean ± SEM (*n* = 3 technical replicates for each group). ***P < 0.0001, NS, not significant (Student’s *t*‐test).Representative H&E images and IF images showing the expression of secretory cell marker CC10, DATP marker CLDN4, or AT1 cell marker CAV1 in organoids derived from *Scgb1a1* lineage‐labeled secretory cells with or without hydroxytamoxifen‐induced ATF4 overexpression. CC10 (green), CLDN4 (white), CAV1 (white), and DAPI (blue). Scale bars, 100 μm.Quantification of *Scgb1a1* lineage‐labeled tdTomato^+^CC10^+^ secretory cells (red), tdTomato^+^CLDN4^+^ DATPs (green), and tdTomato^+^CAV1^+^ AT1 cells (blue) in (G). Data are presented as mean ± SEM (*n* = 3 technical replicates for each group). ***P < 0.0001 (Student’s *t*‐test).

Finally, we validated whether ATF4 activity regulated by YAP/TAZ activation is essential for fate conversion of secretory cells. To do this, we generated the airway cell line overexpressing YAP 5SA mutant in which all five Lats1/2 target sites are mutated (Kim *et al*, 2015). Consistent with Lats1/2 dKO lungs, constitutive activation of YAP signaling enhanced the EAA uptake with increased expression of ATF4 and its target genes, including Slc7a5 (Fig 5A–C). However, knockdown (KD) of ATF4 caused the defects in EAA uptake with reduced expression of Slc7a5 (Fig 5A and C). Furthermore, ATF4 KD in organoids derived from *Lats1*/*2*‐deficient secretory cells blocked the fate conversion of secretory cells into DATPs and AT1 cells (Fig 5D–F). We also confirmed that Slc7a5 KD in Lats1/2 dKO secretory organoids inhibited the transition into DATPs and AT1 cells (Fig 5D–F). In contrast, sustained overexpression of ATF4 in secretory organoids significantly promoted the fate conversion of secretory cells into DATPs and AT1 cells (Fig 5D and G, and H). These results strongly support that ATF4 activity induced by YAP/TAZ signaling is crucial for fate conversion of secretory cells into DATPs and AT1 cells via regulation of Slc7a5/Lat1 expression, allowing EAA uptake.

### Aberrant activation of YAP/TAZ and mTORC1 signaling in human lungs of BO syndrome

**Figure 6:**
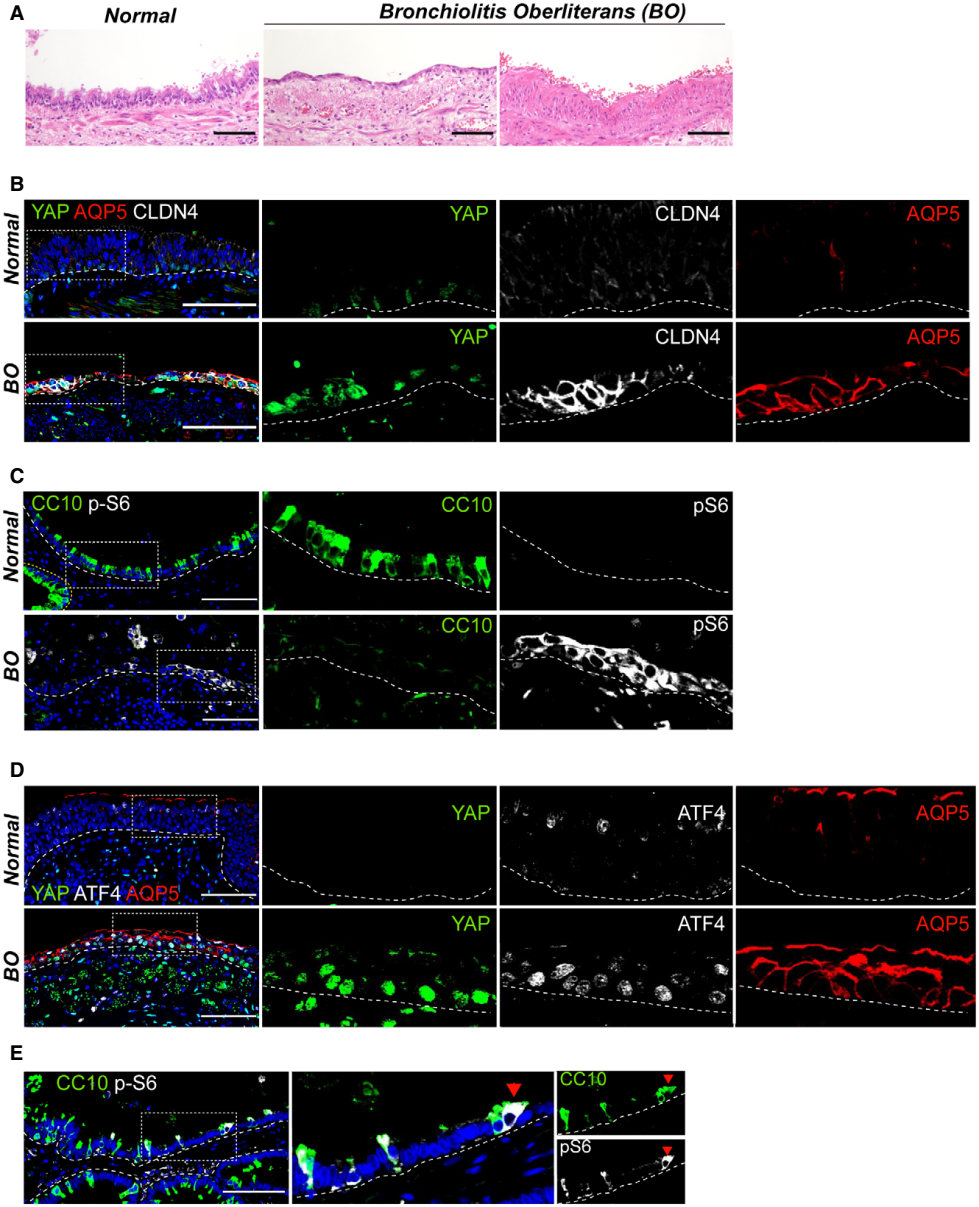
Aberrant activation of YAP/TAZ‐mTORC1 signaling in the human lungs of bronchiolitis obliterans syndrome (BO) Representative H&E images of human lung tissues from normal background and bronchiolitis obliterans (BO) with progressive fibrosis.Representative IF images showing the expressions of nuclear YAP, DATP marker CLDN4, and AT1 cell marker AQP5 in the airways of normal background (top) and BO (bottom) human lungs. CLDN4 (white), AQP5 (red), YAP (green), and DAPI (blue). Of note, a flattened airway layer in BO lungs. Scale bars, 100 μm.Representative IF images showing the expressions of secretory cell marker CC10 and mTORC1 activation marker p‐S6 in the airways of normal background (top) and BO (bottom) human lungs. CC10 (green), p‐S6 (white), and DAPI (blue). Scale bars, 100 μm.Representative IF images showing the expression of nuclear YAP, ATF4, and AQP5 in the airways of normal background (top) and BO (bottom) human lungs. YAP (green), ATF4 (white), AQP5 (red), and DAPI (blue). Of note, co‐expressions of nuclear YAP, nuclear ATF4, and AQP5 in the airways of BO lungs. Scale bars, 100 μm.Representative IF images showing the expression of p‐S6 (white) in the airway cells losing CC10 (green) expression of BO human lungs. Scale bars, 100 μm.

Dysregulation of airway secretory cell maintenance is implicated in human lung diseases such as BO; characterized by severe fibrosis with collagen deposition and loss of epithelial integrity (Liu *et al*, 2019). Our data demonstrated that persistent activation of YAP/TAZ leads to fate changes of secretory cells, coincided with peribronchiolar fibrosis (Appendix Fig S1D). We therefore asked if YAP/TAZ and mTORC1‐ATF4 signaling are activated in the lungs of patients with PF, including BO. In the distal airways of normal regions, the expression of nuclear YAP was barely detected (Fig 6A and B). However, we found a marked increase in nuclear YAP protein in the airways of BO where there were flattened epithelial layers and evident subepithelial fibrosis (Fig 6A and B). Significantly, DATP‐like cells expressing CLDN4 and AT1‐like cells expressing AQP5 were readily detected in this region, whereas they were barely seen in the airways of normal regions (Fig 6A and B). Moreover, we found increased levels of p‐S6 in the airway epithelium of BO lungs accompanied by the loss of secretory cells (Fig 6C). Nuclear ATF4 expression was also correlated with the nuclear expression of YAP in this altered epithelium of BO lungs (Fig 6D). Notably, we readily detected an early transitioning region with partial loss of CC10 expression in secretory cells showing higher expression of p‐S6, implying that acquisition of mTORC1 activity may trigger the loss of secretory cell identity in the airways of BO (Fig 6E).

**Figure EV4:**
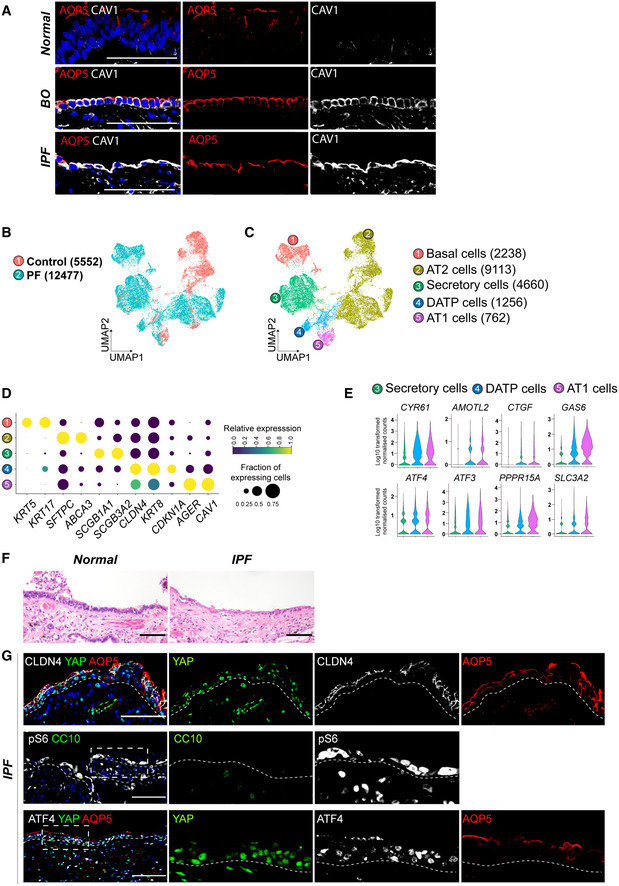
Aberrant activation of YAP/TAZ‐mTORC1 signaling in the human lungs of idiopathic pulmonary fibrosis (IPF) Representative IF images showing the expressions of AT1 cell marker AQP5 and CAV1 in the airways of normal background (top), IPF (middle), and BO (bottom) human lungs. AQP5 (red), CAV1 (white), and DAPI (blue). Scale bars, 100 μm.UMAP plot showing single cells from normal background (control, 5552) and pulmonary fibrosis (PF, 12477) of human lung tissues (GSE135893).UMAP plot representing 5 color‐coded cell clusters identified in merged single‐cell transcriptomes from control and PF lungs.Blob plot depicting selected marker genes in each cell cluster. Dot size encodes the percentage of cells expressing the genes, and color encodes the average per cell gene expression level.Violin plots showing normalized expression levels of YAP/TAZ targets (*CYR61, AMOTL2, CTGF, GAS6*) and mTORC1‐ATF4 targets (*ATF4, ATF3, PPPR15A, SLC3A2*).Representative H&E images of human airway from normal background or IPF. Scale bars, 100 μm.Representative IF images showing the expression of YAP, ATF4, DATP marker CLDN4, AT1 cell marker AQP5, secretory cell marker CC10, and mTORC1 activity marker p‐S6 in human lungs of IPF. YAP (green, top and bottom), ATF4 (white, bottom), CLDN4 (white, top), AQP5 (red, top and bottom), CC10 (green, middle), p‐S6 (white, middle), and DAPI (blue). Scale bars, 100 μm.

Airway epithelium alterations and subepithelial fibrosis are also evident in other types of PF. Therefore, we sought to gain further insights into the role of YAP/TAZ in PF, where we also observed persistent activation of YAP/TAZ‐mTORC1 signaling. We reanalyzed scRNA‐seq data of PF (GSE135893) and identified the presence of DATP‐like cells marked by *CLDN4* and *KRT8* (Habermann *et al*, 2020) (Fig EV4A–D). Notably, this population has been reported as basaloid cells that are aberrantly expanded in human IPF lungs (Habermann *et al*, 2020; Kobayashi *et al*, 2020; Strunz *et al*, 2020). Furthermore, in human PF, we identified elevated transcript levels of YAP/TAZ target genes (*CYR61, AMOTL2, CTGF,* and *GAS6)*, *ATF4* and its targets (*ATF3*, *PPPR15A* and *SLC3A2*), in DATP‐like/basaloid and AT1 cells (Fig EV4E). Finally, we evaluated the potential direct transition of airway secretory cells into DATP‐like and AT1‐like cells by performing IF staining for these lineage markers in IPF lung tissues. The airways of IPF lungs exhibited flattened epithelial morphology, accompanied with subepithelial fibrosis (Fig EV4F). Surprisingly, consistent with BO lungs, nuclear YAP protein was readily observed in the airway epithelium where CLDN4^+^ DATP‐like/basaloid and AQP5^+^ AT1‐like cells were prominent but not CC10^+^ secretory cells. (Fig EV4G). We also verified increased mTORC1‐ATF4 signaling in this altered airway epithelium of IPF lungs (Fig EV4G). Taken together, these results indicate that the aberrant activation of YAP/TAZ in the airway epithelium may modulate the fate conversion of secretory cells into DATP states and AT1 cells via regulating mTORC1‐ATF4 activity, leading to development of peribronchiolar fibrosis in human lung diseases.

## Discussion

The loss of stem cell lineage fidelity leads to disruption and failure in maintaining epithelial barriers, resulting in severe pathological diseases. Here, we demonstrated that sustained activation of YAP/TAZ reprograms airway secretory cells to lose their cellular identity and acquires squamous AT1 cell fate via DATP transitional cell states. This transitional cell states derived from airway secretory cells shared very similar transcriptional signatures of DATPs derived from AT2 cells during alveolar regeneration upon damage signals(Choi *et al*, 2020; Kobayashi *et al*, 2020; Strunz *et al*, 2020). Notably, the fate transition of secretory cells into DATPs and AT1 cells, driven by YAP/TAZ activation, coincided with severe subepithelial fibrosis, strongly suggesting that dysregulation of lineage fidelity within the airway epithelium causes PF. Further, we discovered a core molecular program directing this process. Activation of YAP/TAZ induces mTORC1‐ATF4 activity, enabling amino acid uptake which is required for the fate conversion of secretory cells. Remarkably, elevated YAP and mTORC1‐ATF4 activities were observed in the airway epithelium of human PF lungs, including BO, where the loss of secretory cells and aberrant emergence of DATP‐like and AT1‐like cells within the airways are evident. These results indicate that YAP/TAZ‐mediated mTOR‐ATF4 signaling is a key molecular program regulating the fate behavior of secretory cells in human and mouse lungs, and its dysregulation leads to fibrotic human lung diseases.

By combining lineage‐tracing and scRNA‐seq analysis, we identified the trajectory of secretory cell conversion into AT1 cell fate, driven by YAP/TAZ activation. Airway secretory cells have been reported to give rise to AT2 cells during alveolar regeneration after injury (Rock *et al*, 2011a; Barkauskas *et al*, 2013). However, in the context of sustained YAP/TAZ activation, we found no evidence of the role for AT2 cells mediating the differentiation of secretory cells into AT1 cells. Instead, we discovered CLDN4^+^ DATPs mediated this process, similar to a previous study which suggested a new route for secretory to AT1 fate transition via an alveolar differentiation intermediate (ADI) during alveolar regeneration after injury (Strunz *et al*, 2020). Importantly, together with previous studies showing AT1 cell differentiation mediated by YAP/Hippo signaling during lung development and regeneration (Mahoney *et al*, 2014; Zhao *et al*, 2014; Lange *et al*, 2015; Lin *et al*, 2015; Nantie *et al*, 2018; LaCanna *et al*, 2019; van Soldt *et al*, 2019; Penkala *et al*, 2021), our study indicates that YAP/TAZ is a key regulator in directing AT1 cell fate in the lung. Secretory cell‐derived AT1 cells by YAP/TAZ activation showed similar transcriptional signatures compared to bona fide AT1 cells in our scRNA‐seq analysis. However, whether the proper functional features of AT1 cells converted from secretory cells in the airways are comparable to those in the alveoli remains unanswered.

**Figure EV5:**
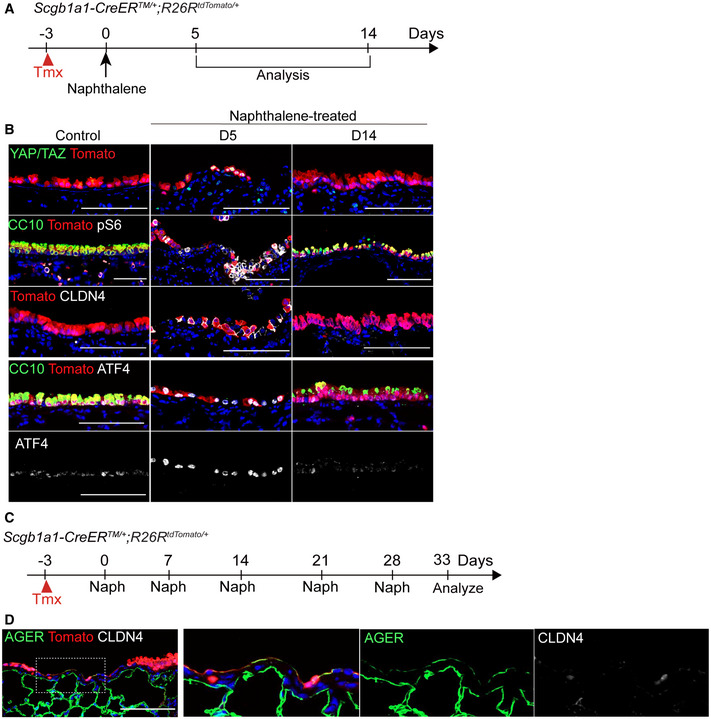
Sustained YAP/TAZ‐mTORC1‐ATF4 signaling by chronic injury caused fate conversion of secretory cells into AT1 cells in the airways Experimental schematic diagram of naphthalene‐induced acute injury in *Scgb1a1‐CreER^TM^* ^/+^ *;R26R^tdTomato^* ^/+^ mice post tamoxifen treatment.Representative IF images showing the expression of YAP and TAZ, secretory cell marker CC10, DATP marker CLDN4, mTORC1 activity marker p‐S6, and ATF4 in *Scgb1a1* lineage‐labeled tdTomato^+^ cells in control and naphthalene‐treated lungs. YAP/TAZ (green, 1^st^ panel), CC10 (green, 2^nd^ and 4^th^ panels), p‐S6 (white, 2^nd^ panel), CLDN4 (white, 3^rd^ panel), ATF4 (white, 4^th^ and 5^th^ panels). Scale bars, 100 μm.Experimental schematic diagram of repetitive naphthalene‐induced chronic injury in *Scgb1a1‐CreER^TM^* ^/+^ *;R26R^tdTomato^* ^/+^ mice post tamoxifen treatment.Representative IF images showing the expression of AT1 cell marker AGER and DATP marker CLDN4 in *Scgb1a1* lineage‐labeled tdTomato^+^ cells in control and naphthalene‐treated lungs. AGER (green), CLDN4 (white), and DAPI (blue). Scale bars, 100 μm.

The loss of secretory cells and/or their squamous alterations have been suggested to cause a severe fibrotic eruption in the distal airways. Previous studies reported that the transient activation of YAP/TAZ promotes regeneration of the airway epithelium (Lange *et al*, 2015; Sun *et al*, 2019). Further, it is noted that YAP is activated in transitional cell states during lung injury repair or in disease cell states (Gokey *et al*, 2018; Choi *et al*, 2020; Kobayashi *et al*, 2020; Strunz *et al*, 2020). Based on these reports and our observations, we propose that YAP/TAZ signaling is a core program regulating the differentiation of DATP cell states, which arise from either AT2 or airway secretory cells in a spatiotemporal manner during lung regeneration. Interestingly, we observed transient upregulation of mTORC1‐ATF4 activity with enhanced nuclear YAP/TAZ localization during airway injury repair post naphthalene treatment (Fig EV5A and B). In accordance with YAP/TAZ activation, secretory cells lost their cellular identity and transiently acquired CLDN4^+^ DATP cell states prior to repopulating secretory cells for injury resolution. However, repetitive naphthalene injuries caused impaired injury repair and the loss of epithelial integrity. DATPs were persisted, and, of note, we observed the fate conversion of lineage‐labeled secretory cells into AT1 cells in the airway epithelium, which is similar to the phenotype of Lats1/2 dKO lungs (Fig EV5C and D). These results suggest that persistent YAP/TAZ activation induced by chronic lung damage promotes fate conversion of secretory cells into DATPs/AT1 cells, which could be directly implicated in lung diseases. Indeed, we observed increased expression of nuclear YAP/TAZ in the airway epithelium of human PF, where the loss of cellular integrity coinciding with the accumulation of DATP‐like and AT1‐like cells was evident. Further, the emergence of DATP cell states after YAP/TAZ activation coincided with the observation of severe subepithelial fibrosis in Lats1/2 dKO mouse lungs. As previously suggested, it is likely that prolonged presence of DATPs by sustained YAP activation establishes fibrotic microenvironments by secreting pro‐fibrotic factors, such as *Tgfb1*, *Ctgf*, and *Edn1*, which may cause the subepithelial fibrotic lesions (Choi *et al*, 2020; Habermann *et al*, 2020; Kobayashi *et al*, 2020; Strunz *et al*, 2020). Thus, a better understanding of the cellular events and molecular programs governing the fate decisions of secretory cells by YAP/TAZ activation, and its impacts on inducing remodeling of surrounding microenvironments, could potentially enhance our insight into human lung diseases.

Our scRNA‐seq analysis revealed that the regulation of secretory cell fate driven by YAP/TAZ activation requires dynamic regulation of metabolism; the synthesis and uptake of amino acids were highly elevated in DATP cell states. Our *ex vivo* organoid results strongly suggested the crucial role of extracellular EAAs in directing the YAP/TAZ‐mediated fate transition of secretory cells into DATPs and AT1 cells. Notably, the expressions of amino acid transporters, such as *Slc7a5*/*Lat1,* are highly enriched in DATPs driven by YAP/TAZ activation. YAP has been reported to directly regulate the expression of *Slc7a5*/*Lat1* (Hansen *et al*, 2015; Park *et al*, 2016; Edwards *et al*, 2017). However, our study suggests that mTORC1 signaling is essential for YAP/TAZ‐mediated upregulation of *Slc7a5*/*Lat1,* enabling the promotion of EAA uptake in airway secretory cells. Notably, both amino acid withdrawals and pharmacological inhibition of SLC7A5/LAT1 significantly alleviated YAP/TAZ‐induced fate conversion of secretory cells into AT1 cells.

We further provide an *in vivo* demonstration that activation of mTOR‐dependent ATF4 signaling, mediated by YAP/TAZ activation, is essential for the fate conversion of secretory cells into DATPs and AT1 cells. YAP/TAZ is known to modulate mTORC1 activity, which acts as gatekeepers in cellular differentiation and growth (Tumaneng *et al*, 2012; Hansen *et al*, 2015; Hu *et al*, 2017). We found that inactivation of mTORC1 signaling, by ablating *Raptor* in YAP/TAZ‐activated lungs, suppressed upregulation of ATF4, the loss of secretory cell identity, and the emergence of DATPs and AT1 cells. Remarkably, genetic and pharmacologic inhibition of mTORC1 activity substantially suppressed aberrant fibrotic lesions by YAP/TAZ activation in secretory cells. These results suggest the direct interconnection between epithelial lineage alterations and the development of subepithelial fibrosis in the lungs. Significantly, despite the activation of an mTORC1‐ATF4 axis in *Lats1*/*2*‐deficient secretory cells, they failed to convert into DATPs and AT1 fate in the absence of EAAs. These results strongly suggest that mTOR‐ATF4 activity mediated by YAP/TAZ is required for fate conversion of secretory cells into DATPs and AT1 fate by modulating the expression of amino acid transporters such as *Slc7a5*/*Lat1,* which enables EAA uptake.

BO syndrome is a poorly understood airway disease characterized by severe fibrotic bronchiolar occlusions (Liu *et al*, 2019). The lack of understanding of the molecular mechanisms for its initiation and progression limits the therapeutic interventions possible for BO patients. Previous studies suggested that damage to secretory cells, or decrease of CC10 expression in secretory cells, are related to BO (Nord *et al*, 2002; Bourdin *et al*, 2012; Kelly *et al*, 2012; Liu *et al*, 2019). Importantly, we discovered aberrant YAP and mTORC1‐ATF4 signaling in the airway epithelium of human BO lungs with the loss of secretory cells and emergence of CLDN4^+^ DATP‐like and AQP5^+^ AT1‐like cells in the airways. These were strikingly similar observations to those made in our LATS1/2 dKO mouse model. Our findings suggest the YAP/TAZ‐mTORC1‐ATF4 axis as a potential therapeutic target for human fibrotic lung diseases such as BO by modulating the differentiation program of secretory or DATP cells in PF lungs.

In summary, our results identify the molecular and cellular mechanisms governing the fate and identity of airway secretory cells, which are critical for maintaining epithelial integrity and functions. The regulatory axis between YAP/TAZ and mTORC1‐ATF4 signaling determines the fate behaviors of secretory cells in the lungs. Hyperactivation of this axis causes lineage infidelity of secretory cells, coupled with severe subepithelial fibrosis. Our study provides clues for potential therapeutic targets of mTORC1 signaling in the lung diseases of severe fibrosis, including BO lungs.

## Materials and Methods

### Human tissues

For histological analysis, human distal lung parenchymal tissues with BO and IPF were retrieved from surgical pathology archives, with the approval of the institutional review board of Yonsei University Severance Hospital, Seoul, Korea. The fibrotic lung diseases were classified according to the American Thoracic Society and European Respiratory Society criteria. After histologic review, paraffin‐embedded lung tissues from 5 patients with IPF and 5 patients with BO were collected and analyzed. We defined regions with normal morphology as “normal.”

### Animals

*Lats1^fl^* ^/^ *^fl^* mice (Heallen *et al*, 2011) were generated and kindly provided by Dr. Randy L. Johnson. *Yap^fl^* ^/^ *^fl^* (Xin *et al*, 2011) and *Taz^fl^* ^/^ *^fl^* mice (Xin *et al*, 2013) were generated and generously donated by Dr. Eric N. Olson. *Lats2^fl^* ^/^ *^fl^* mice were generated in our laboratory (Kim *et al*, 2013). *Scgb1a1‐CreER^TM^* mice (Rawlins *et al*, 2009), *R26‐LSL‐tdTomato* mice (Madisen *et al*, 2010), and *Raptor^fl^* ^/^ *^fl^* mice (Sengupta *et al*, 2010) were purchased from Jackson Laboratory. For analysis, C57BL/6J male mice were used throughout the study at the age of 4–5 weeks. Mice were bred and maintained under specific pathogen‐free conditions. Experiments were performed in accordance with the procedures approved by Korea Advanced Institute of Science and Technology‐Animal Care and Use Committee, approved by local ethical review committees, and conducted according to UK Home Office project license PC7F8AE82.

### Chemical treatment in mice

Tamoxifen (CAY‐13258‐2, Cayman) was dissolved in sterile corn oil at a 25 mg/ml dosage and administered into mice through intraperitoneal injection at a 0.25 mg/g body weight. AZD8055 mTORC1/2 inhibitor (HY‐10422, MedChemExpress) was injected into mice via intraperitoneal injection 24 h prior to tamoxifen treatment at a 10 mg/kg dose. After tamoxifen injection, AZD8055 was reinjected 6 h later. Then, AZD8055 inhibitor was continuously injected every 12 h until the indicated day of tissue collection. AZD8055 was dissolved in the solution of 5% Tween20, 10% DMSO, 40% PEG300, and 45% saline. Naphthalene (176044, Sigma) was dissolved in corn oil and administered to mice through intraperitoneal injection at a 250 mg/kg body weight. Single dose of naphthalene was injected and analyzed at day 5 and 14 post treatment. For repetitive injuries, naphthalene was injected once a week for 5 weeks, and tissues were analyzed at 5 days after a final dose.

### Tissue sample preparation for histology

Lung tissues were exsanguinated using filtered PBS and inflated with freshly prepared 4% paraformaldehyde (PFA). After incubation for 2–4 h at 4°C, lung tissues were washed with PBS at 4°C for 16 h. Lung lobes were cut in half, to process half of the tissue for paraffin blocks and the half for frozen blocks. For formalin‐fixed paraffin (FFPE) blocks, tissue was dehydrated in ethanol, cleared with xylene, then embedded in paraffin. For frozen tissue blocks, tissues were submerged in 30% sucrose for 2–3 days at 4°C. Tissues were embedded in the optimal cutting temperature (OCT) compound and stored at −80°C. Frozen tissue blocks were sectioned using a Leica cryostat into 10 µm thick sections and attached to Superfrost Plus™ Adhesion Microscope slides (ThermoFisher Scientific, J1800AMNT). Paraffin blocks were sectioned into 4 µm thick sections.

### Hematoxylin and Eosin staining

Hematoxylin and Eosin staining was performed on 4 µm thick FFPE‐embedded tissue sections. Briefly, slides were deparaffinized and hydrated. The sections were then placed in Mayer’s Hematoxylin (Sigma HHS32) for 30 s. Slides were placed in HCl and rinsed excessively in tap water. The intensity of the hematoxylin was checked under a bright microscope. Sections were then advanced to ammonium chloride, dehydrated, and mounted in DPX mountant (44581, Sigma Aldrich).

### Sirius Red staining

Collagen fibers were observed in lung tissues, as previously reported (Gomer Ronkainen *et al*, 2021). Briefly, tissue samples were deparaffinized and incubated overnight at room temperature with a saturated picric acid (Sigma 80456) with 0.1% Fast Green (Sigma F7252) and 0.1% Direct Red (Sigma 365548). Sections were repeatedly washed with distilled water, then dehydrated and mounted with DPX Mounting.

### Immunofluorescent staining

FFPE sections were deparaffinized, hydrated, and emerged in PBS. OCT‐embedded frozen sections were directly submerged in PBS for 5 min. After antigen retrieval with citrate acid (0.01 M, pH6.0), blocking was performed with 10% donkey serum in 0.2% Triton‐X/PBS (PBST) at room temperature (RT) for 1 h. Primary antibodies were diluted in 0.2% PBST at appropriate dilution (Refer to Table 1) and incubated overnight at 4°C. For immunofluorescence, Alexa‐Fluor‐488, ‐594, or ‐647 were used as 2’ab and incubated for 1 h at RT or 25 min at 37°C. Nuclei were stained with 4,6‐diamidino‐2‐phenylindole (DAPI, Sigma D9542). Slides were then mounted in ProLong Gold antifade reagent (Invitrogen P36930) and sealed with cover glass (Marienfeld, 0101172 or 0101192).

**Table 1:** The antibodies used for immunostaining.

Antibody	Dilution	Sample prep	Company	Cat. No.
CC10	1:200	Frozen/FFPE	SantaCruz	sc‐9772
CC10	1:100	Frozen/FFPE	SantaCruz	sc‐365992
Acetylated‐tubulin	1:1000	Frozen/FFPE	Sigma‐Aldrich	T7451
p63	1:100	Frozen	Abcam	AB735
SPC	1:200	Frozen/FFPE	Millipore	AB3786
AGER	1:100	Frozen/FFPE	R&D Systems	MAB1179
CAVEOLIN‐1	1:200	FFPE	Cell Signaling	3238
AQUAPORIN5	1:400	FFPE	Abcam	78486
T1α	1:300	Frozen	DSHB	8‐1‐1
CLAUDIN4	1:100	Frozen	Invitrogen	36‐4800
tdTomato	1:500	Frozen/FFPE	Sicgen	AB8181‐200
YAP	1:100	Frozen/FFPE	Novus	H00010413
TAZ	1:300	Frozen/FFPE	Sigma‐Aldrich	HPA007415
Phospho‐S6 (S235/236)	1:100	FFPE	Cell Signaling	2211S
ATF4	1:100	FFPE	Novus	NBP2‐67766

### Image analysis and Cell counting

Microscope slides were imaged on Leica TCS SP5, Zeiss LSM800, and Zeiss LSM880 confocal imaging system. For tissue images, at least three different sections, including at least two different bronchial regions from three individual mice per group, were used. For 3D organoid coculture images, qualitative images were taken at a 20× magnification without zoom and were processed using ImageJ. Quantifications were performed in a blinded fashion using ImageJ and the cell counter plugin. Statistical significance was determined by student’s unpaired *t*‐test and set to *P* < 0.05 or 0.1. Raw data was recorded, and graphs were generated in GraphPad Prism 5.

### Quantitative Real‐Time PCR

A quantitative PCR reaction mix was prepared using cDNA from lineage‐labeled cells, SYBR qPCR mixture, and primers listed below. Comparative C_T_ (ΔΔC_T_) experiment was run on a Bio‐Rad Connect machine, using Gapdh as endogenous control and cDNA samples from lineage‐labeled cells as reference samples. Data were analyzed using the ΔΔC_T_ quantitation method. ImageJ was used for quantifying the relative gene expression from gel images. The primers used for qRT‐PCR are as follows:

**Table:** 

Target	Forward (5′–3′)	Reverse (5′–3′)
*Ctgf*	AGCTGACCTGGAGGAAAACA	GACAGGCTTGGCGATTTTAG
*Cyr61*	CTGCGCTAAACAACTCAACGA	GCAGATCCCTTTCAGAGCGG
*Thbs1*	GGTAGCTGGAAATGTGGTGCGT	GCACCGATGTTCTCCGTTGTGA
*Tgfb2*	CTCCGACGTGACAGACGCT	GCAGGGGCAGTGTAAACTTATT
*Atf4*	ATGGCCGGCTATGGATGAT	CGAAGTCAAACTCTTTCAGATCCATT
*Trib3*	CTGCGTCGCTTTGTCTTCAGCA	CTGAGTATCTCTGGTCCCACGT
*Slc7a1*	GTCTATGTCCTAGCCGGTGC	GAGCCTAGGAGACTGGTGGA
*Slc7a3*	ATTTGCTTTCTCCGAGGGCA	ATACCCAGCTCCAACACACG
*Slc7a5*	GGTCTCTGTTCACGTCCTCAAG	GAACACCAGTGATGGCACAGGT
*Slc3a2*	GAGCGTACTGAATCCCTAGTCAC	GCTGGTAGAGTCGGAGAAGATG
*Slc7a11*	CTTTGTTGCCCTCTCCTGCTTC	CAGAGGAGTGTGCTTGTGGACA
*Gapdh*	CATCACTGCCACCCAGAAGACTG	ATGCCAGTGAGCTTCCCGTTCAG
*L32*	GGCCTCTGGTGAAGCCCAAGATCG	CCTCTGGGTTTCCGCCAGTTTCGC

### Mouse lung tissue dissociation and flow cytometry

Lung tissues were dissociated with a collagenase/dispase solution as previously described (Lee *et al*, 2014). Briefly, after lungs were exsanguinated using filtered PBS, 2 ml of warm dispase (Corning 354235, 50 U/ml) was injected into lung tissues through the trachea. Each lobe was dissected and chopped excessively with a scissor in a 50 ml falcon tube. Per lung tissue, 3 ml PBS with 60 µl collagenase (Roche 10269638001, diluted to 100 mg/ml in cold PBS) was added and incubated for 35 min in 37°C shaking incubator. 25 µl of DNase I (Sigma D4527, made to 1% solution in sterile water) was added and further incubated in 37°C shaking incubator for 10 min. Dissociated cells were filtered through series of 100 µm and 40 µm filter strainer, and centrifuged at 1,000 rpm for 5 min. The supernatant was aspirated, and the remaining red blood cell (RBC) in the pellet was lysed in 1 ml 1xACK buffer (Lonza 10‐548E) for 90 s at RT. To stop the lysis, 6 ml FBS‐free DMEM was first added, then 500 µl FBS was gently layered to the bottom of the tube. Cells were centrifuged at 1,000 rpm for 5 min, and the cell pellet was resuspended in 10% FBS in PBS (PF10) for further staining. Cell sorting was performed with FACS Aria II (Beckton Dickinson).

The antibodies used for flow cytometry and sorting are as follows:

**Table:** 

Antibody	Fluorophore	Company	Cat. No.
CD45	APC	BD Pharmingen	559864
CD31	APC	BD Pharmingen	551262
EpCAM	PE‐Cy7	Biolegend	118216
KDR	APC	Biolegend	136405

### *Ex* *vivo* 3D lung organoid coculture

Freshly sorted secretory cells were resuspended in culture medium (3D basic medium (DMEM/F12, Gibco) supplemented with 10% FBS (Gibco) and ITS (Insulin‐Transferrin‐Selenium, Corning)). These cells were mixed with cultured lung stromal cells negatively isolated by microbeads of CD326/EpCAM, CD45, and CD31 via MACS (Miltenyi Biotech), followed by resuspension in growth factor‐reduced Matrigel (BD Biosciences) at a ratio of 1:5. A 100 μl mixture was placed in a 24‐well Transwell insert with a 0.4 μm pore (Corning) (Lee *et al*, 2014). Approximately 5 × 10^3^ epithelial cells were seeded in each insert. 500 μl of culture medium was placed in the lower chamber, and the medium was changed every other day. For amino acid media change experiment, 500 µl of 3D basic media, 10% dialyzed FBS in BME media (Thermofisher, 21010046), or 10% dialyzed FBS in BME media with EAA (1 mM of L‐Histidine, L‐Isoleucine, L‐Leucine, L‐Methionine, L‐Threonine, L‐Valine, L‐Glutamine, L‐Arginine, L‐Cysteine) was replaced every day.

### 5’‐Single‐cell RNA library preparation and scRNA‐sequencing analysis

Four mice from each group of day 5 (D5) *Scgb1a1‐CreER^TM^* ^/+^;*R26R^tdTomato^* ^/+^, D5 *Lats1^fl^* ^/^ *^fl^*;*Lats2^fl^* ^/^ *^fl^*;*Scgb1a1‐CreER^TM^* ^/+^;*R26R^tdTomato^* ^/+^, and day 14 (D14) *Lats1^fl^* ^/^ *^fl^*;*Lats2^fl^* ^/^ *^fl^*; *Scgb1a1‐CreER^TM^* ^/+^; *R26R^tdTomato^* ^/+^ were used. Isolated primary lung epithelial cells from the same group were pooled and sorted for DAPI^–^CD31^–^CD45^–^EpCAM^+^tdTom^+^ cells using BD AriaII FACS sorter. The purified tdTom^+^ cells were then processed using the droplet‐based 10× Genomics Chromium approach. In brief, Gel beads in Emulsion (GEMs) were prepared using barcoded Chromium Single Cell 3’Library & Gel Bead Kit v3 (PN1000075) and Chromium B Chip Kit (PN1000154). To recover 2 × 10^3^ cells for control and 4 × 10^3^ cells each for D5 and D14 samples, approximately 5–8 × 10^3^ cells were loaded onto the Chromium controller. Immediately after GEMs formation, they were reverse transcribed, and the resulting cDNAs were tagged with Unique Molecular Index (UMI) and cell barcodes. Next, GEMs were broken, and cDNA was amplified and quantified using Agilent 2100 Bioanalyzer. The final 3’ Gene Expression Library was constructed by enzymatic fragmentation and size selection. The samples were then sequenced on the Illumina HiSeq X by Macrogen with a target of 2.5–3.0 × 10^4^ reads/cells (2 × 100 paired‐end reads).

### Alignment, quantification, and quality control of scRNA‐seq data

Droplet‐based sequencing data were aligned and quantified using the Cell Ranger Single‐Cell Software Suite (version 3.0.2, 10× Genomics Inc) using the *Mus musculus* genome (GRCm38) (official Cell Ranger reference, version 1.2.0). Cells were filtered by custom cutoff (more than 500 and less than 7,000 detected genes, more than 2,000 UMI count) to remove potential empty droplets and doublets. The downstream analysis included data normalization, highly variable gene detection, log transformation, principal component analysis, neighborhood graph generation, and Louvain graph‐based clustering, which was done by python package scanpy (version 1.3.6) (Wolf *et al*, 2018) using default parameters.

### Doublet exclusion

To exclude doublets from scRNA‐seq data, we applied scrubl*et al*gorithm per sample to calculate scrublet‐predicted doublet score per cell with following parameters: sim_doublet_ratio=2; n_neighbors=30; expected_doublet_rate=0.1. Any cell with a scrublet score > 0.7 was flagged as doublet. To propagate the doublet detection into potential false‐negatives from scrublet analysis, we over‐clustered the dataset (*sc*.*tl.louvain* function from scanpy package version 1.3.4; resolution = 20), and calculated the average doublet score within each cluster. Any cluster with averaged scrublet score > 0.6 was flagged as a doublet cluster. All remaining cell clusters were further examined to detect potential false‐negatives from scrublet analysis according to the following criteria: (i) Expression of marker genes from two distinct cell types which are unlikely according to prior knowledge, (ii) higher number of UMI counts.

### Pseudotime analysis

All data contained within our processed Seurat object for the wildtype data set was converted to the AnnaData format for pseudotime analysis in Scanpy (version 1.3.6). We recalculated *k*‐nearest neighbors at k = 15. Pseudotime was calculated using Scanpy’s partitioned‐based graph abstraction function, PAGA. Diffusion pseudotime was performed using Scanpy’s DPT function with default parameters.

### Plasmid construction

For conditional shRNA expression in organoid culture, pSico plasmid was used as cloned as previously reported (Ventura *et al*, 2004). The shRNA sequences used are as follows:

shscramble forward: 5′‐TGACACGCGACTTGTACCACTTCAAGAGAGTGGTACAAGTCGCGTGTCTTTTTTC‐3′, shscramble reverse: 5′‐TCGAGAAAAAAGACACGCGACTTGTACCACTCTCTTGAAGTGGTACAAGTCGCGTGTCA‐3′, shAtf4 forward: 5′‐TGGAGTTAGTTTGACAGCTATTCAAGAGATAGCTGTCAAACTAACTCCTTTTTTC‐3′, shAtf4 reverse: 5′‐ TCGAGAAAAAAGGAGTTAGTTTGACAGCTATCTCTTGAATAGCTGTCAAACTAACTCCA‐3′, shSlc7a5 forward: 5′‐ TGCAGCCTGCAATCCTAATATTCAAGAGATATTAGGATTGCAGGCTGCTTTTTTC‐3′, shSlc7a5 reverse: 5′‐ TCGAGAAAAAAGCAGCCTGCAATCCTAATATCTCTTGAATATTAGGATTGCAGGCTGCA‐3′.

For Atf4 overexpression, pMSCV‐ERT2‐Atf4‐IRES‐EGFP was constructed in‐house. Mouse Atf4 coding sequence (CDS) was PCR amplified from mouse cDNA using the following primer set: 5′‐GGTACCGAGCTCGGATCCAACATGACCGAGATGAGCTTCC‐3′ and 5’‐GCTGGATATCTGCAGAATTCTTACGGAACTCTCTTCTTCCC‐3’. The Atf4 CDS, ERT2, and IRES‐EGFP DNA fragments were assembled and cloned into *EcoR*I/*BamH*I‐digested pMSCV plasmid using the In‐Fusion Cloning kit (Takara), and confirmed by DNA sequencing.

### Amino acid uptake assay

L‐amino acid assay kit (Sigma, ab65347, for total L‐amino acid) and branched chain amino acid (BCAA) assay kit (Sigma, ab83374, for EAA) were used to measure amino acid content in 3D organoids or C22 murine airway cell lines. For 3D organoids, Matrigel was first removed by dispase (Corning 354235, 50 U/ml) incubation for 40 min. tdTomato‐expressing organoids were picked with a pipette under a fluorescent microscope to exclude cocultured endothelial cells. The selected organoids or C22 cells were further dissociated into single cells using TrypLE (Gibco 2604021), and the number of cells was counted using Countess II automated cell counter. 1 × 10^6^ cells were each used to measure total L‐amino acids or BCAAs contents according to the manufacturer’s protocols.

### Statistical analysis

Values are presented as mean ± SEM. All experiments used at least 2–5 mice per group due to the individual variation between mice. For JPH203 or AZD8055 chemical treatment, male mice of 4–6 weeks were randomly assigned to treatment or control groups. Statistical significance was determined by student’s unpaired *t*‐test and set to *P* < 0.05 or 0.1. All graphs were generated with GraphPad Prism 5.

## Author contributions

**Hae Yon Jeon:** Conceptualization; Resources; Data curation; Formal analysis; Funding acquisition; Investigation; Writing—original draft; Writing—review & editing. **Jinwook Choi:** Resources; Software; Formal analysis; Investigation; Writing—original draft; Writing—review & editing. **Lianne Kraaier:** Validation; Investigation. **Young Hoon Kim:** Investigation. **David Eisenbarth:** Validation; Visualization. **Kijong Yi:** Software. **Ju‐Gyeong Kang:** Resources. **Jin Woo Kim:** Resources. **Hyo Sup Shim:** Resources. **Joo‐Hyeon Lee:** Conceptualization; Formal analysis; Supervision; Funding acquisition; Writing—original draft; Writing—review & editing. **Dae‐Sik Lim:** Conceptualization; Supervision; Funding acquisition; Writing—original draft; Writing—review & editing.

In addition to the CRediT author contributions listed above, the contributions in detail are:

H‐YJ, JC, DSL, JHL, conceived and designed the experiments, interpreted the data, and wrote the manuscript; HYJ performed most of the experiments and data analysis; JC analyzed murine scRNA‐seq data; DE. reanalyzed human scRNA‐seq data; LK designed the experiments, performed IF analysis, and interpreted IF image data; YHK helped with genotyping and staining of qKO mice and qPCR of C22 cell lines; KY aligned raw scRNA‐seq data; JGK constructed Atf4 overexpressing plasmid; HSS provided and analyzed pathology of human IPF tissue; JWK shared Raptor^fl/fl^ mice.

## Supporting information



Appendix

Expanded View Figures PDF

Source Data for Expanded View

Source Data for Figure 4

## Data Availability

The Gene Expression Omnibus accession number for raw expression data for scRNA‐seq analysis of *Lats1^fl^* ^/^ *^fl^*;*Lats2^fl^* ^/^ *^fl^*;*Scgb1a1‐CreER^TM^* ^/+^;*R26R^tdTomato^* ^/+^ mice reported in the manuscript is GSE178829 (https://www‐ncbi‐nlm‐nih‐gov.ezproxy.u‐pec.fr/geo/query/acc.cgi?acc=GSE178829). The Rds files for control and idiopathic PF (IPF) lungs were obtained from GEO (GSE135893; https://www‐ncbi‐nlm‐nih‐gov.ezproxy.u‐pec.fr/geo/query/acc.cgi?acc=GSE135893) (Habermann *et al*, 2020). Cell clusters of “Club,” “CLDN4^+^/KRT8^+^,” and “AT1” cells were extracted and analyzed.
