# Supplementary material for: Airway secretory cell fate conversion via YAP‐mTORC1‐dependent essential amino acid metabolism
Source: EMBO J. 2022 Mar 14;41(8):e109365. doi: 10.15252/embj.2021109365 (PMC9016350; doi:10.15252/embj.2021109365)
Supplement: Supplementary file 1 — Appendix [file EMBJ-41-e109365-s005.pdf]

Appendix

Airway secretory cell fate conversion via YAP-mTORC1-dependent essential amino acid metabolism

Hae Yon Jeon[1,8], Jinwook Choi[2,8]. Lianne Kraaier[2,3], Young Hoon Kim[1], David Eisenbarth[1], Kijong Yi[4,5], Ju-Gyeong Kang[1], Jin Woo Kim[1], Hyo Sup Shim[6], Joo-Hyeon Lee[2,7,*], Dae-Sik Lim[1,*]

Table of Content

- **Appendix Figure S1. Activation of YAP/TAZ by *Lats1/2* deletion leads to peribronchiolar fibrosis**
- **Appendix Figure S2. Unfiltered single cell RNA sequencing results**

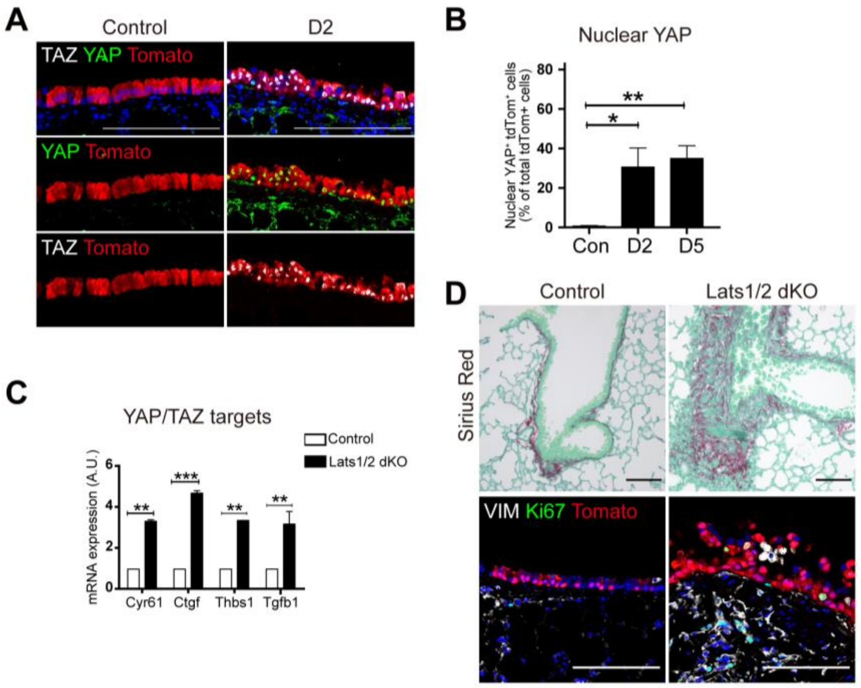

Appendix Figure S1. Activation of YAP/TAZ by *Lats1/2* deletion leads to peribronchiolar fibrosis

A Representative IF images showing the expression of YAP and TAZ in control and Lats1/2 dKO lungs at day 2 post tamoxifen treatment. Tomato (for secretory lineage, red), TAZ (white), YAP (green), and DAPI (blue). Scale bars, 100µm.

B Quantification of nuclear YAP in *Scgb1a1* lineage-labeled cells of control and Lats1/2 dKO lungs at day 2 and 5 post tamoxifen treatment. Data are presented as mean ± SEM (n=3, each group). *p<0.05, **p<0.01.

C qPCR analysis of YAP/TAZ target genes (*Cyr61*, *Ctgf*, *Thbs1*, *Tgfb1*) in freshly isolated *Scgb1a1* lineage-labeled cells of control and Lats1/2 dKO lungs at day 2 post tamoxifen treatment. Data are presented as mean ± SEM (n=3, each group). **p<0.01, ***p<0.001.

D Representative images of Sirius red staining for collagen (top) and IF images of proliferating mesenchymal cells (bottom) in control and Lats1/2 dKO lungs at day 7 post tamoxifen treatment. Tomato (for secretory lineage, red), VIM (Vimentin for mesenchyme, white), Ki67 (for proliferating cells, green), and DAPI (blue). Scale bars, 100µm.

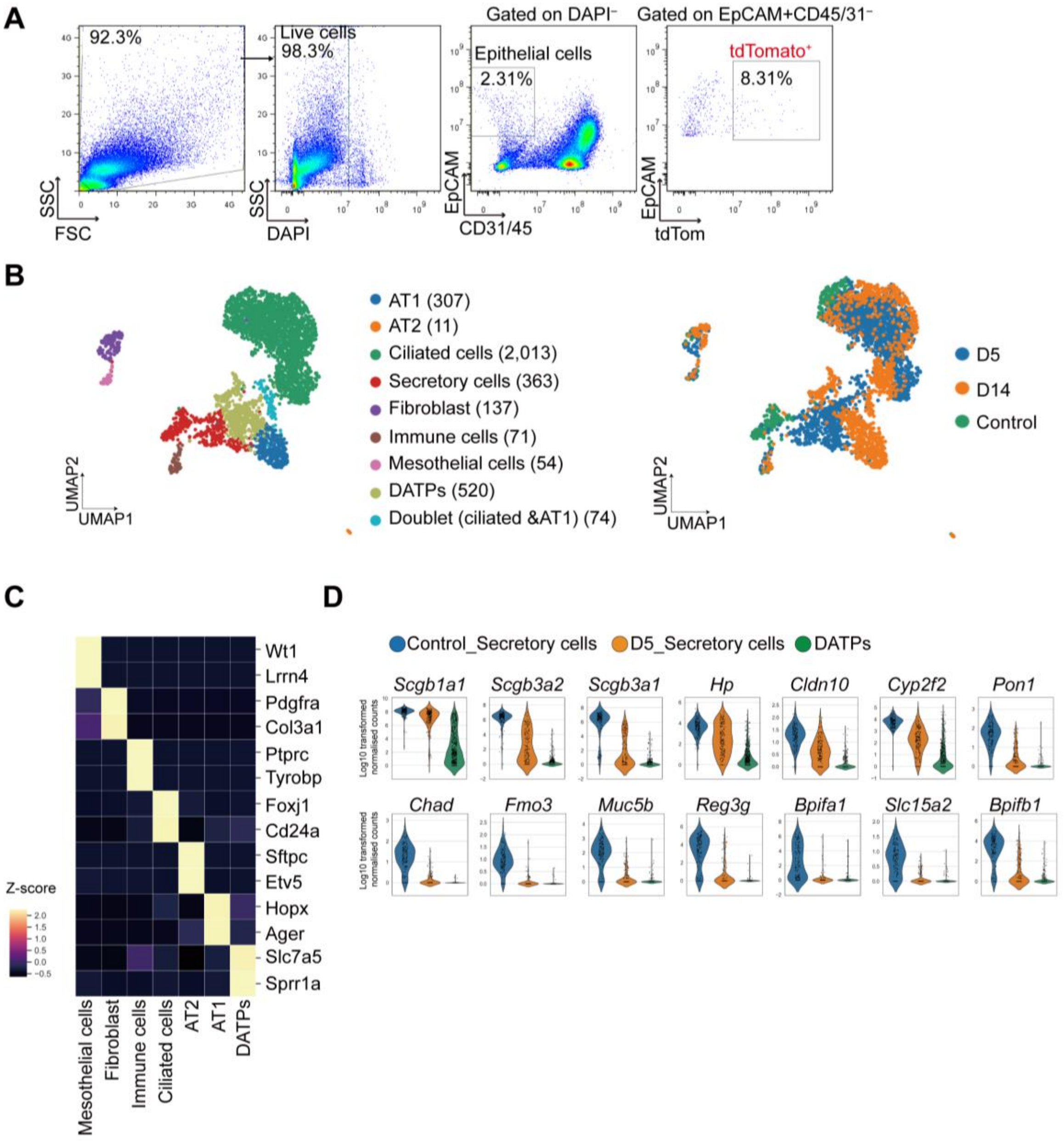

Appendix Figure S2. Unfiltered single cell RNA sequencing results

A Flow cytometry sorting scheme for isolating *Scgb1a1* lineage-labeled tdTomato$^+$ cells from control lungs at day 5 post tamoxifen treatment and Lats1/2 dKO lungs at day 5 and 14 post tamoxifen treatment for scRNA-sequencing.

B UMAP plot showing unfiltered cell clusters identified in merged single-cell transcriptomes from control and Lats1/2 dKO lungs at day 5 and 14 post tamoxifen treatment.

C Heatmap of representative cell marker genes of each cell cluster.

D Violin plot of secretory cell markers in secretory cells and DATP cells of control and Lats1/2 dKO lungs. Of note, most of the secretory marker genes are dramatically lost in *Lats1/2*-deficient secretory cells at day 5 post tamoxifen induction.
