# Supplementary material for: Airway secretory cell fate conversion via YAP‐mTORC1‐dependent essential amino acid metabolism
Source: EMBO J. 2022 Mar 14;41(8):e109365. doi: 10.15252/embj.2021109365 (PMC9016350; doi:10.15252/embj.2021109365)
Supplement: Supplementary file 2 — Expanded View Figures PDF [file EMBJ-41-e109365-s003.pdf]

Expanded View Figures

Figure EV1. YAP/TAZ activation in secretory cells enforces fate conversion into AT1 cells, but bypasses basal or AT2 cells.

A Representative IF images showing the expression of epithelial cell marker E-cadherin and apoptosis marker TUNEL in *Scgb1a1* lineage-labeled $tdTomato^+$ cells of control and Lats1/2 dKO lungs. Tomato (for secretory lineage, red), E-cadherin (for epithelial cell, white), TUNEL (for apoptotic cells, green), and DAPI (blue). Scale bars, 100 μm.

B Representative IF images showing the expression of basal cell marker P63 in *Scgb1a1* lineage-labeled $tdTomato^+$ cells in control and Lats1/2 dKO lungs. Tomato (for secretory lineage, red), P63 (green), and DAPI (blue). Scale bars, 100 μm.

C Representative IF images showing the expression of AT2 cell marker SPC in *Scgb1a1* lineage-labeled $tdTomato^+$ cells in control and Lats1/2 dKO lungs. Tomato (for secretory lineage, red), SPC (green), and DAPI (blue). Scale bars, 100 μm.

D, E Representative IF images showing the expression of AT1 cell markers T1α (D), AGER (E), and endothelial marker VECAM (E) in *Scgb1a1* lineage-labeled $tdTomato^+$ cells in control and Lats1/2 dKO lungs. Tomato (for secretory lineage, red), T1α (D, green), AGER (E, green), VECAM (E, white), and DAPI (blue). Scale bars, 100 μm.

F Quantification of *Scgb1a1* lineage-labeled $tdTomato^+AGER^+$ AT1 cells in (E). Data are presented as mean ± SEM ($n = 5$ mice for each group). $*P < 0.05$, $***P < 0.001$ (Student's t-test).

© 2022 The Authors

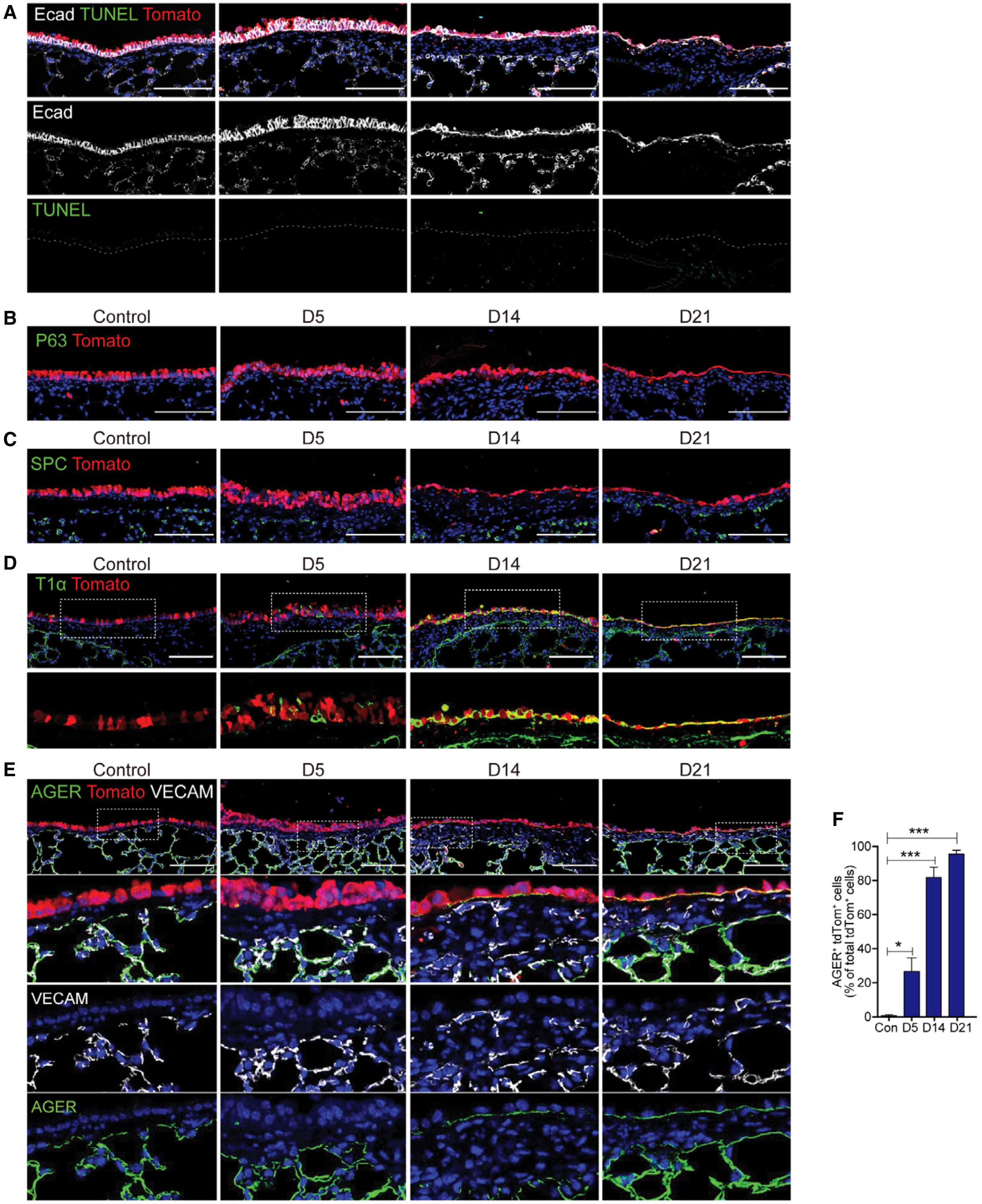
A
Ecad TUNEL Tomato
Ecad
TUNEL
B
Control
D5
D14
D21
P63 Tomato
C
SPC Tomato
D
T1α Tomato
E
AGER Tomato VECAM
VECAM
AGER
F
AGER+ tdTom+ cells (% of total tdTom+ cells)
0
20
40
60
80
100
*
***
***
Con
D5
D14
D21

Figure EV1.

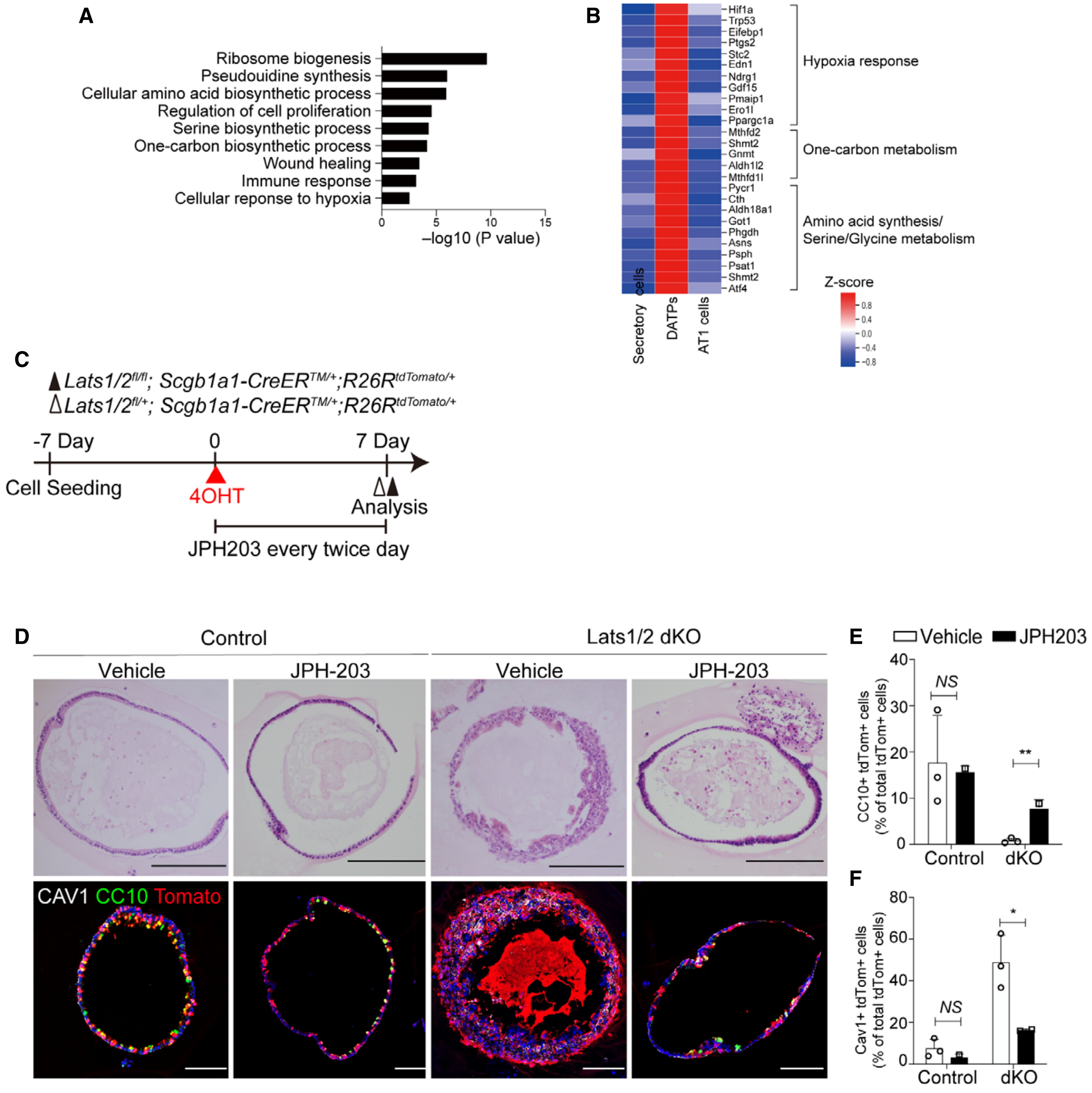

Figure EV2. YAP/TAZ signaling modulates the uptake of essential amino acids that are crucial for cell fate conversion of secretory cells.

A Analysis of Gene Ontology (GO) terms in DATP cell states driven by YAP/TAZ activation.

B Heatmap of secretory, DATPs, and AT1 cells for metabolism-related genes including hypoxia response, one-carbon metabolism, and amino acid synthesis/serine/glycine metabolism.

C Experimental designs for organoid cultures derived from secretory cells isolated from unlabeled control and Lats1/2 dKO lungs. Isolated secretory cells were seeded in 3D organoid cocultures with stromal cells. At day 7 post seeding, 4-hydroxytamoxifen (4-OHT) was treated, followed by the addition of SLC7A5/LAT1 inhibitor (JPH203) twice every day for 7 days. Organoids were analyzed at day 7 post 4-OHT treatment.

D Representative H&E and IF images of 3D organoids in (C). Tomato (red), CC10 (green), CAV1 (for AT1 cells, white), and DAPI (blue). Scale bars, 100 μm.

E, F Quantification of *Scgb1a1* lineage-labeled tdTomato^{+}CC10^{+} secretory (E) and CAV1^{+} AT1 (F) cells in 3D organoids in (D). Data are presented as mean ± SEM (n = at least 2 technical replicates per group). *$P < 0.05$, **$P < 0.01$, NS, not significant (Student's t-test).

 © 2022 The Authors

Figure EV3. Ablation of *Raptor* suppresses YAP/TAZ-induced mTORC1 and ATF4 activation.

A, B Representative H&E and IF images (A), and quantification (B) of secretory cells and mTORC1 activity in control, Lats1/2 dKO (dKO), and Raptor;Lats1/2 triple knockout (tKO) airways at day 5 post tamoxifen treatment. Tomato (A, for Scgb1a1 lineage, red), CC10 (A, green), pS6 (A, white), and DAPI (blue). Scale bars, 100 μm. Data are presented as mean ± SEM ($n = 3$ mice for each genotype). $^{**}P < 0.01$, $^{***}P < 0.0001$ for $pS6^{+}CC10^{-}$ cells (Student's *t*-test).

C Representative IF images showing the expression of nuclear YAP and ATF4 in *Scgb1a1*$^{+}$ lineage-labeled cells of control, Lats1/2 dKO, and Raptor;Lats1/2 tKO airways at day 5 post tamoxifen treatment. Tomato (for Scgb1a1 lineage, red), YAP (green), ATF4 (white), and DAPI (blue). Scale bars, 100 μm.

D Experimental designs for treating mTOR inhibitor (AZD8055) or vehicle to control and Lats1/2 dKO mice twice a day at indicated time points post tamoxifen treatment.

E Representative H&E and IF images of control and Lats1/2 dKO lungs with vehicle or AZD8055 treatment at day 14 post tamoxifen treatment. Tomato (for secretory lineage, red), CC10 (middle, green), AGER (bottom, green), and DAPI (blue). Scale bars, 100 μm.

F Quantification of *Scgb1a1* lineage-labeled $tdTomato^{+}CC10^{+}$ secretory (top) and $AGER^{+}$ AT1 cells (bottom) in (E). Data are presented as mean ± SEM ($n = 3$ mice for each group). $^{**}P < 0.005$, $^{***}P < 0.001$ (Student's *t*-test).

G Representative Sirius red staining for collagen (top) and IF images showing the expression of mesenchymal marker Vimentin (bottom) in control and Lats1/2 dKO lungs with vehicle or AZD8055 treatment. Tomato (for secretory lineage, red), vimentin (VIM, green), and DAPI (blue). Scale bars, 100 μm.

Source data are available online for this figure.

© 2022 The Authors

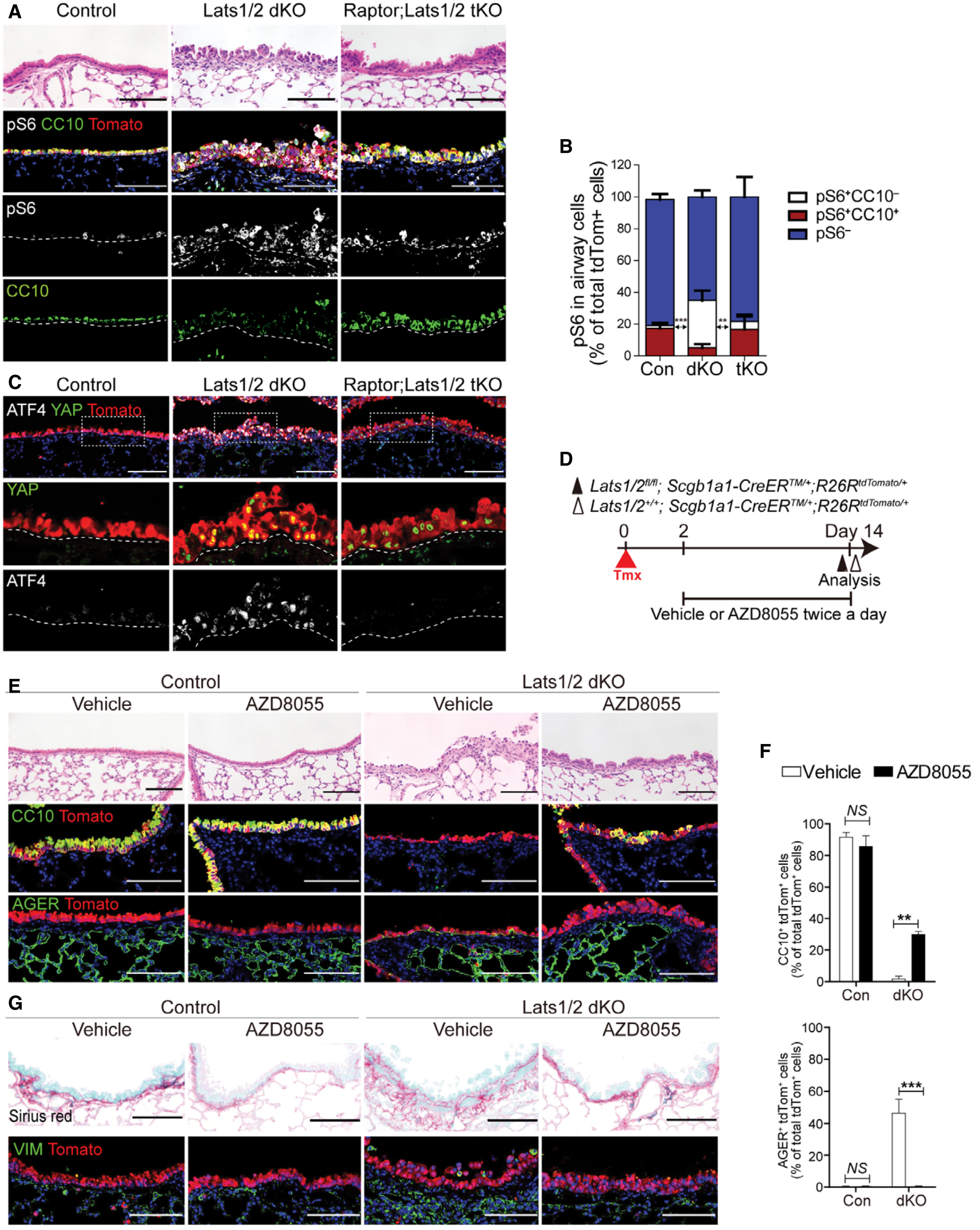

Figure EV3.

 © 2022 The Authors

Figure EV4. Aberrant activation of YAP/TAZ-mTORC1 signaling in the human lungs of idiopathic pulmonary fibrosis (IPF).

A Representative IF images showing the expressions of AT1 cell marker AQP5 and CAV1 in the airways of normal background (top), IPF (middle), and BO (bottom) human lungs. AQP5 (red), CAV1 (white), and DAPI (blue). Scale bars, 100 μm.

B UMAP plot showing single cells from normal background (control, 5552) and pulmonary fibrosis (PF, 12477) of human lung tissues (GSE135893).

C UMAP plot representing 5 color-coded cell clusters identified in merged single-cell transcriptomes from control and PF lungs.

D Blob plot depicting selected marker genes in each cell cluster. Dot size encodes the percentage of cells expressing the genes, and color encodes the average per cell gene expression level.

E Violin plots showing normalized expression levels of YAP/TAZ targets (*CYR61, AMOTL2, CTGF, GAS6*) and mTORC1-ATF4 targets (*ATF4, ATF3, PPPR15A, SLC3A2*).

F Representative H&E images of human airway from normal background or IPF. Scale bars, 100 μm.

G Representative IF images showing the expression of YAP, ATF4, DATP marker CLDN4, AT1 cell marker AQP5, secretory cell marker CC10, and mTORC1 activity marker p-S6 in human lungs of IPF. YAP (green, top and bottom), ATF4 (white, bottom), CLDN4 (white, top), AQP5 (red, top and bottom), CC10 (green, middle), p-S6 (white, middle), and DAPI (blue). Scale bars, 100 μm.

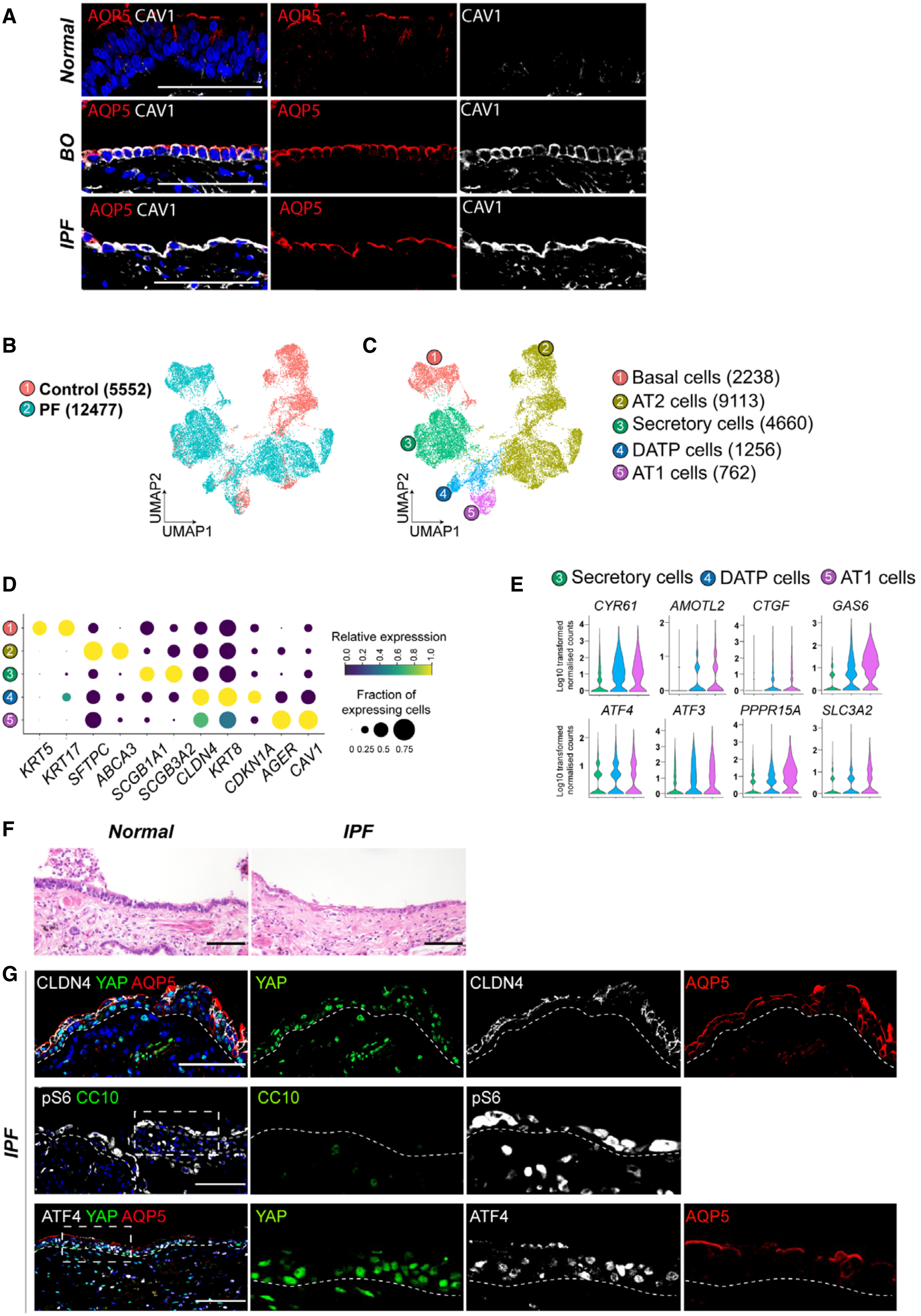

Figure EV4.

© 2022 The Authors

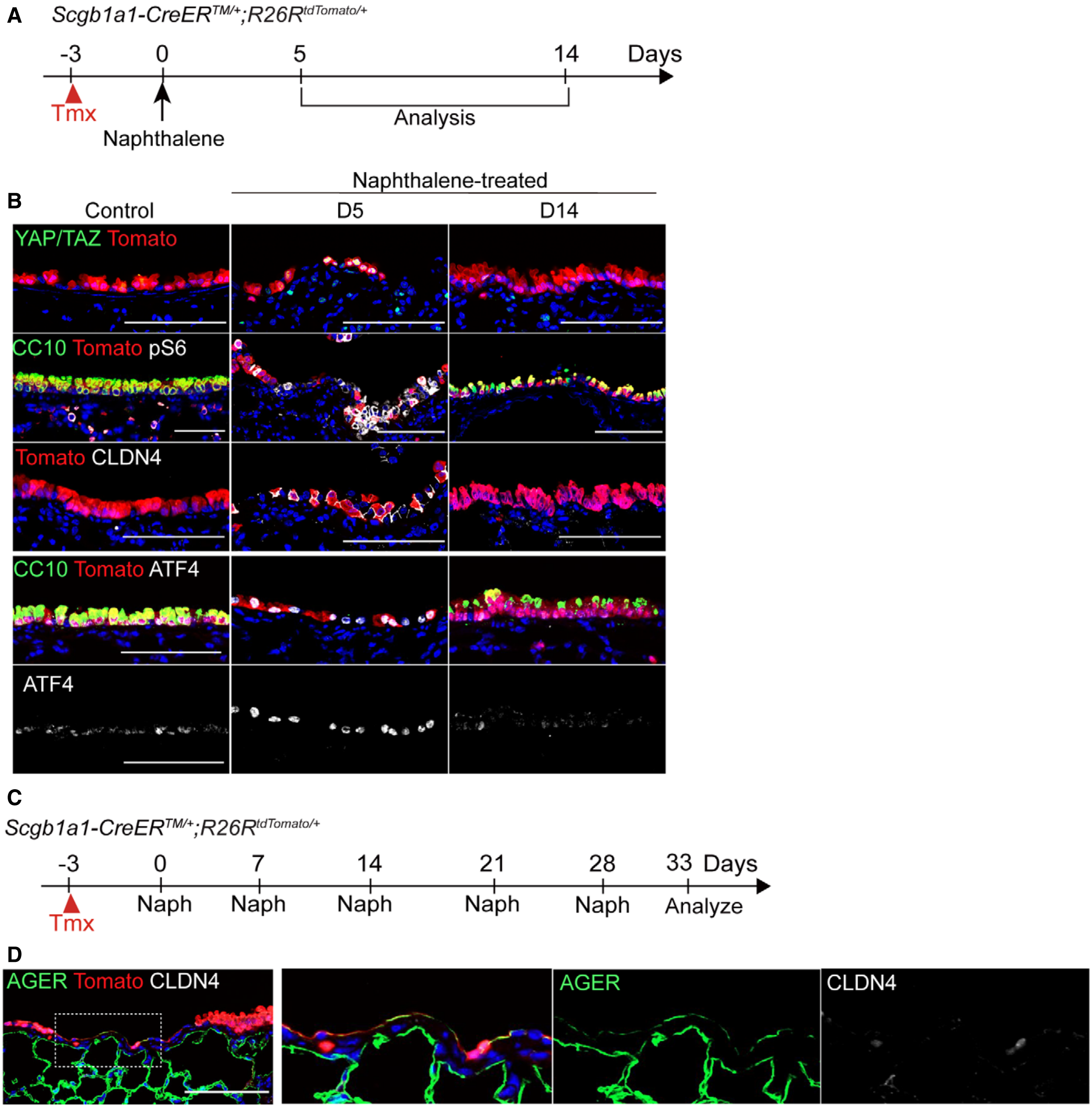

Figure EV5. Sustained YAP/TAZ-mTORC1-ATF4 signaling by chronic injury caused fate conversion of secretory cells into AT1 cells in the airways.

A Experimental schematic diagram of naphthalene-induced acute injury in *Scgb1a1-CreER*$^{TM/+}$;*R26R*$^{tdTomato/+}$ mice post tamoxifen treatment.

B Representative IF images showing the expression of YAP and TAZ, secretory cell marker CC10, DATP marker CLDN4, mTORC1 activity marker p-S6, and ATF4 in *Scgb1a1* lineage-labeled tdTomato^{+} cells in control and naphthalene-treated lungs. YAP/TAZ (green, 1st panel), CC10 (green, 2nd and 4th panels), p-S6 (white, 2nd panel), CLDN4 (white, 3rd panel), ATF4 (white, 4th and 5th panels). Scale bars, 100 μm.

C Experimental schematic diagram of repetitive naphthalene-induced chronic injury in *Scgb1a1-CreER*$^{TM/+}$;*R26R*$^{tdTomato/+}$ mice post tamoxifen treatment.

D Representative IF images showing the expression of AT1 cell marker AGER and DATP marker CLDN4 in *Scgb1a1* lineage-labeled tdTomato^{+} cells in control and naphthalene-treated lungs. AGER (green), CLDN4 (white), and DAPI (blue). Scale bars, 100 μm.

© 2022 The Authors
