# Supplementary figures and images for: Airway secretory cell fate conversion via YAP‐mTORC1‐dependent essential amino acid metabolism
Source: EMBO J. 2022 Mar 14;41(8):e109365. doi: 10.15252/embj.2021109365 (PMC9016350; doi:10.15252/embj.2021109365)

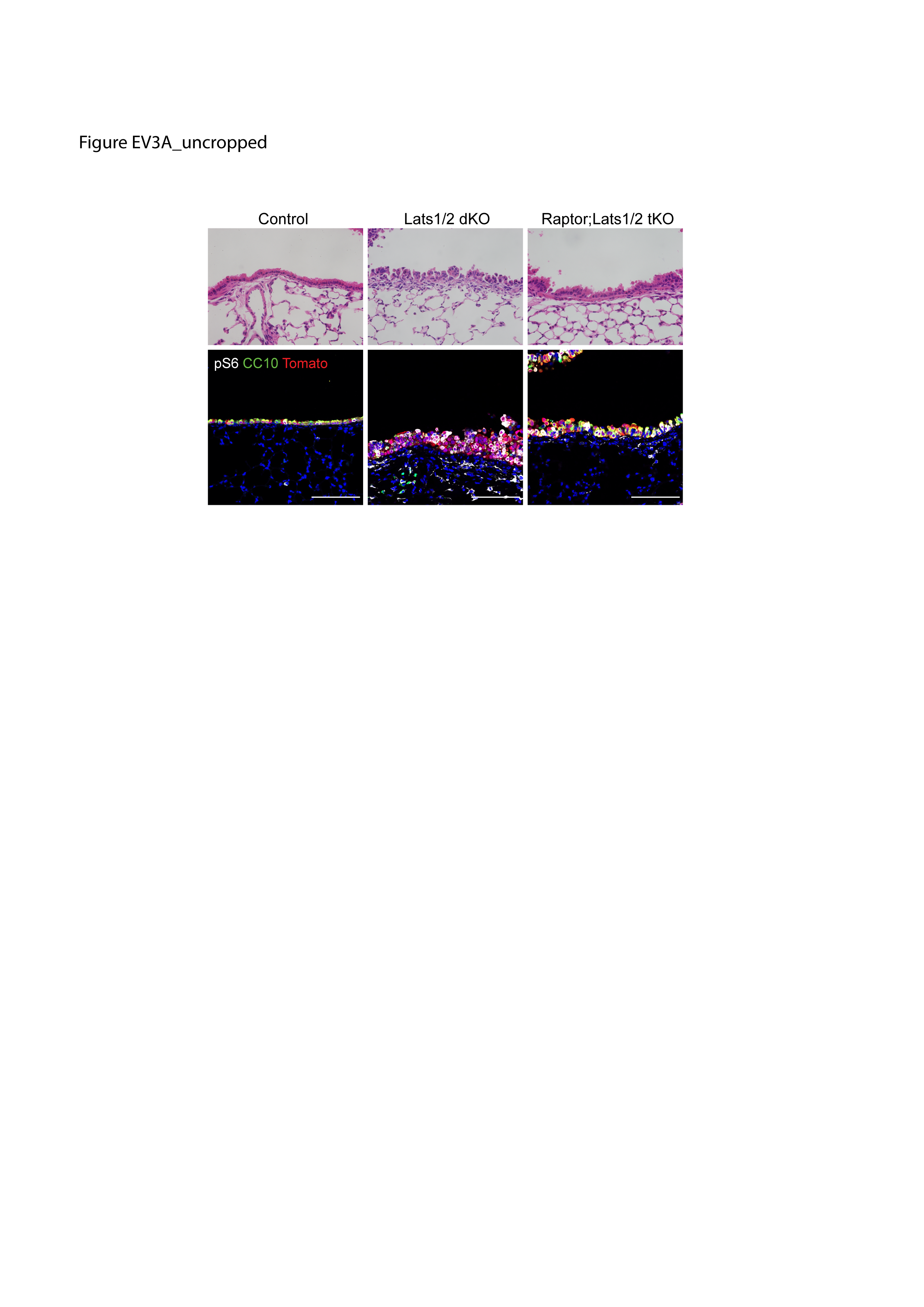

Supplement: Supplementary file 3 — Source Data for Expanded View [file EMBJ-41-e109365-s001.zip › EMBOJ-2021-109365R1-Figure_EV3A_Source_Data-sd-2.tif]

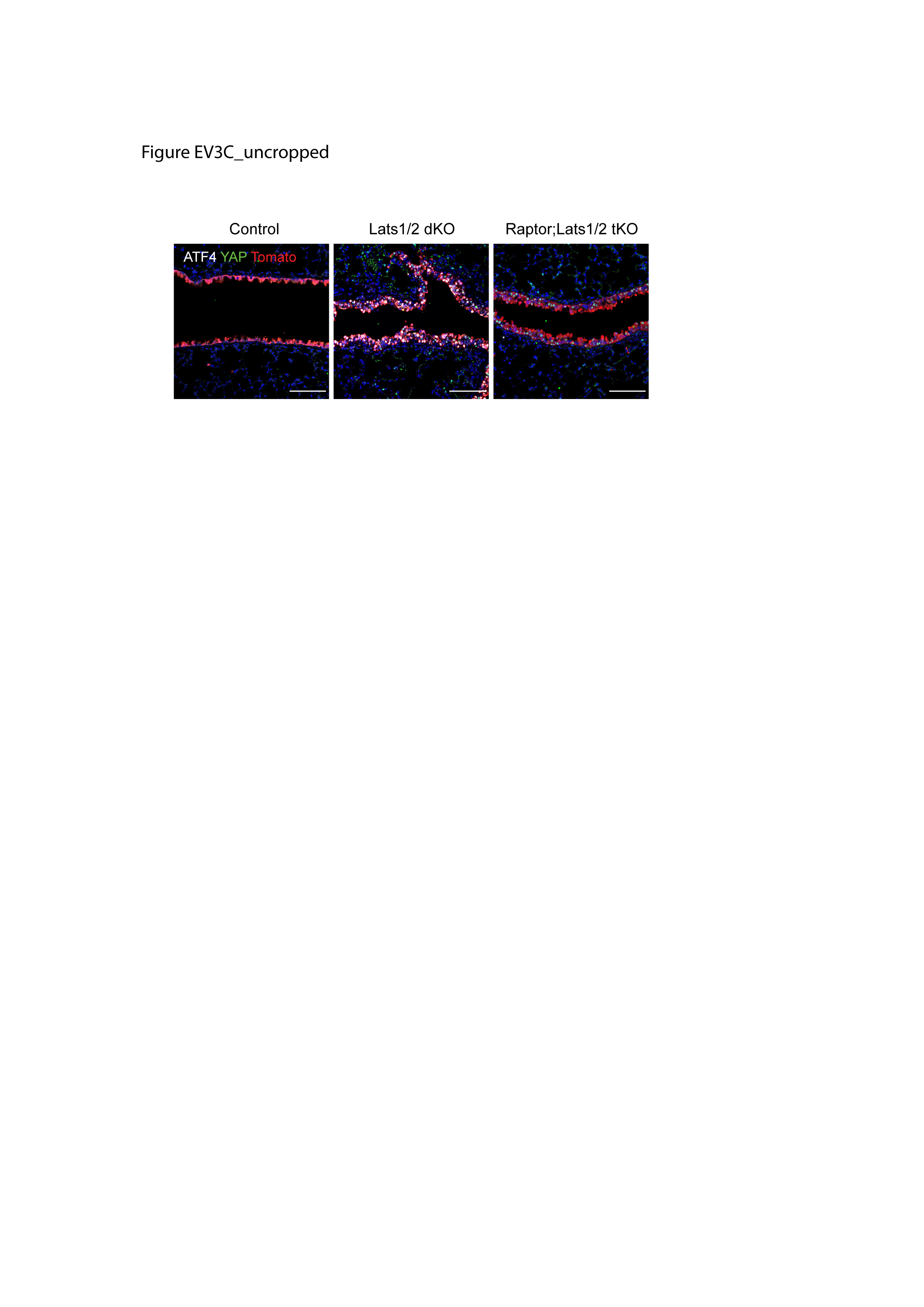

Supplement: Supplementary file 3 — Source Data for Expanded View [file EMBJ-41-e109365-s001.zip › EMBOJ-2021-109365R1-Figure_EV3C_Source_Data-sd.tif]

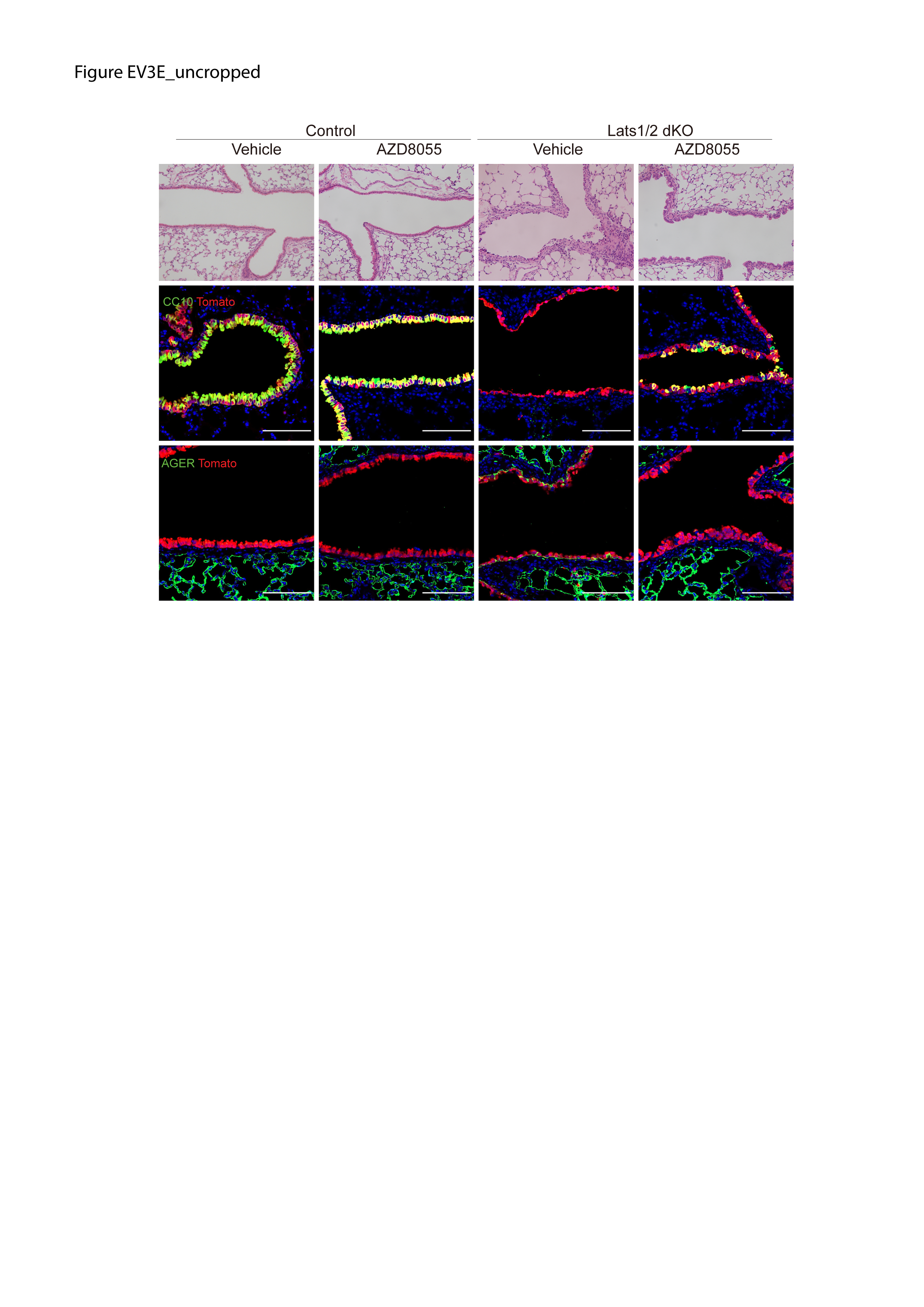

Supplement: Supplementary file 3 — Source Data for Expanded View [file EMBJ-41-e109365-s001.zip › EMBOJ-2021-109365R1-Figure_EV3E_Source_Data-sd.tif]

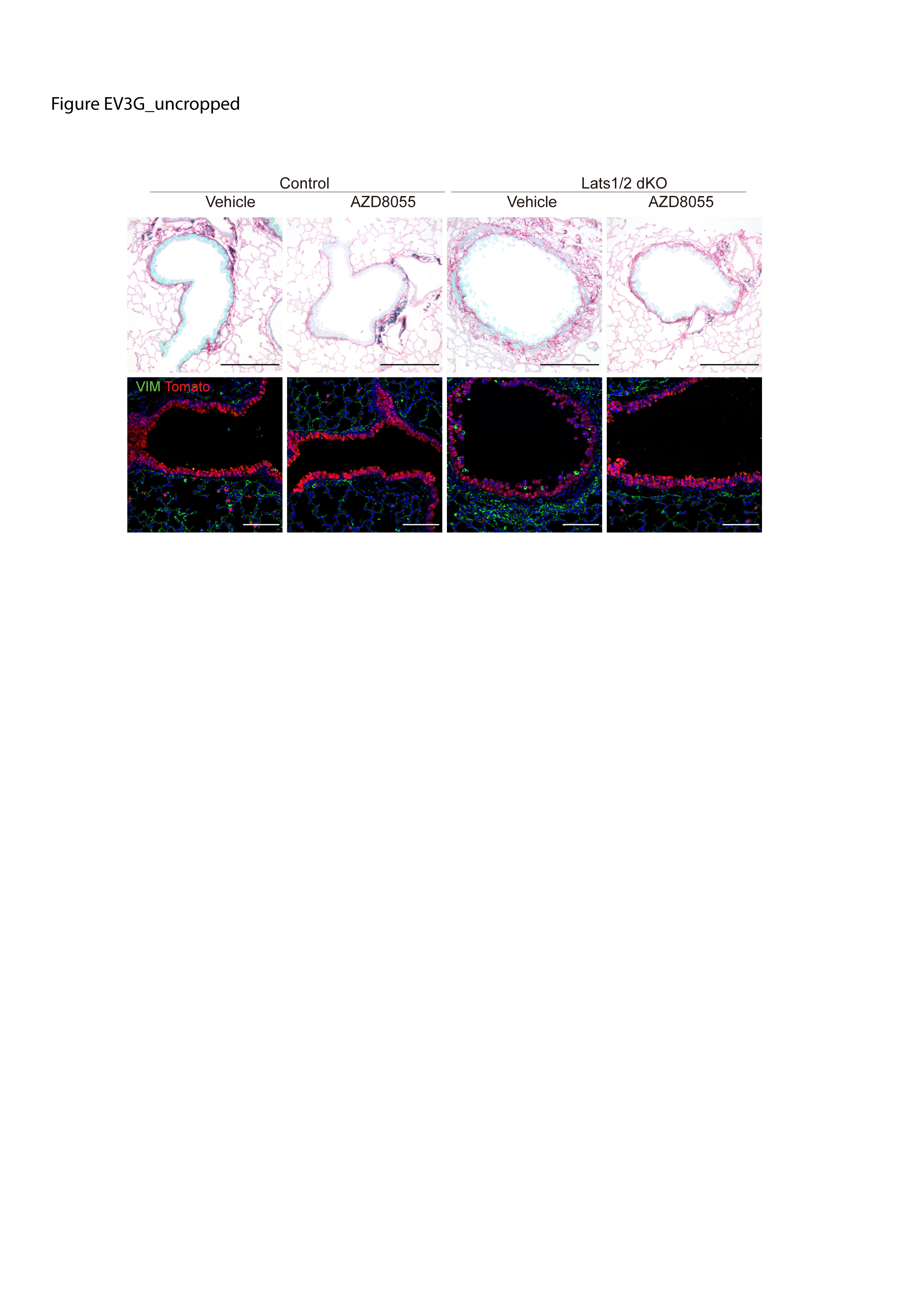

Supplement: Supplementary file 3 — Source Data for Expanded View [file EMBJ-41-e109365-s001.zip › EMBOJ-2021-109365R1-Figure_EV3G_Source_Data-sd.tif]

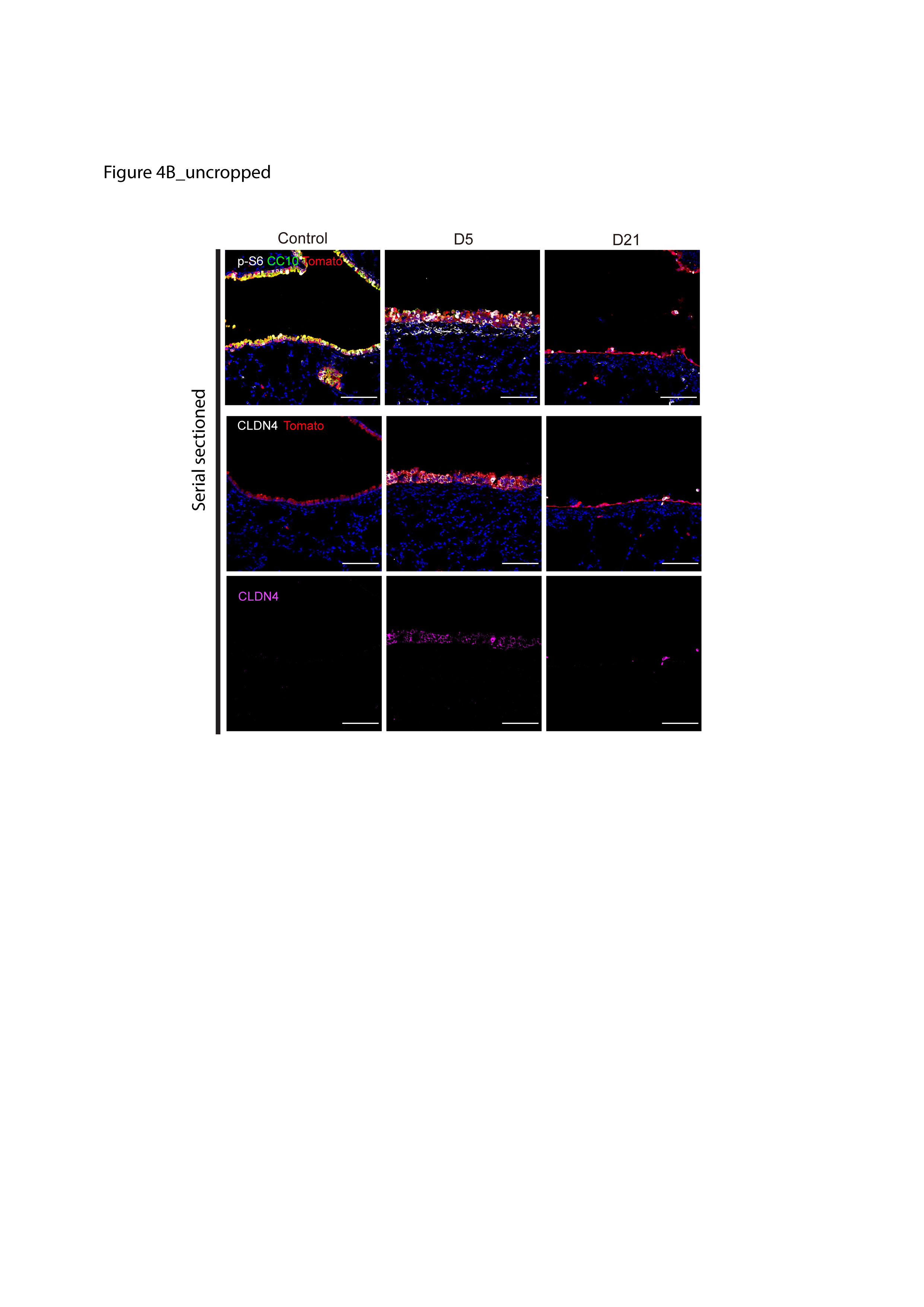

Supplement: Supplementary file 4 — Source Data for Figure 4 [file EMBJ-41-e109365-s002.zip › EMBOJ-2021-109365R1-Figure_4B_Source_Data-sd(3).tif]

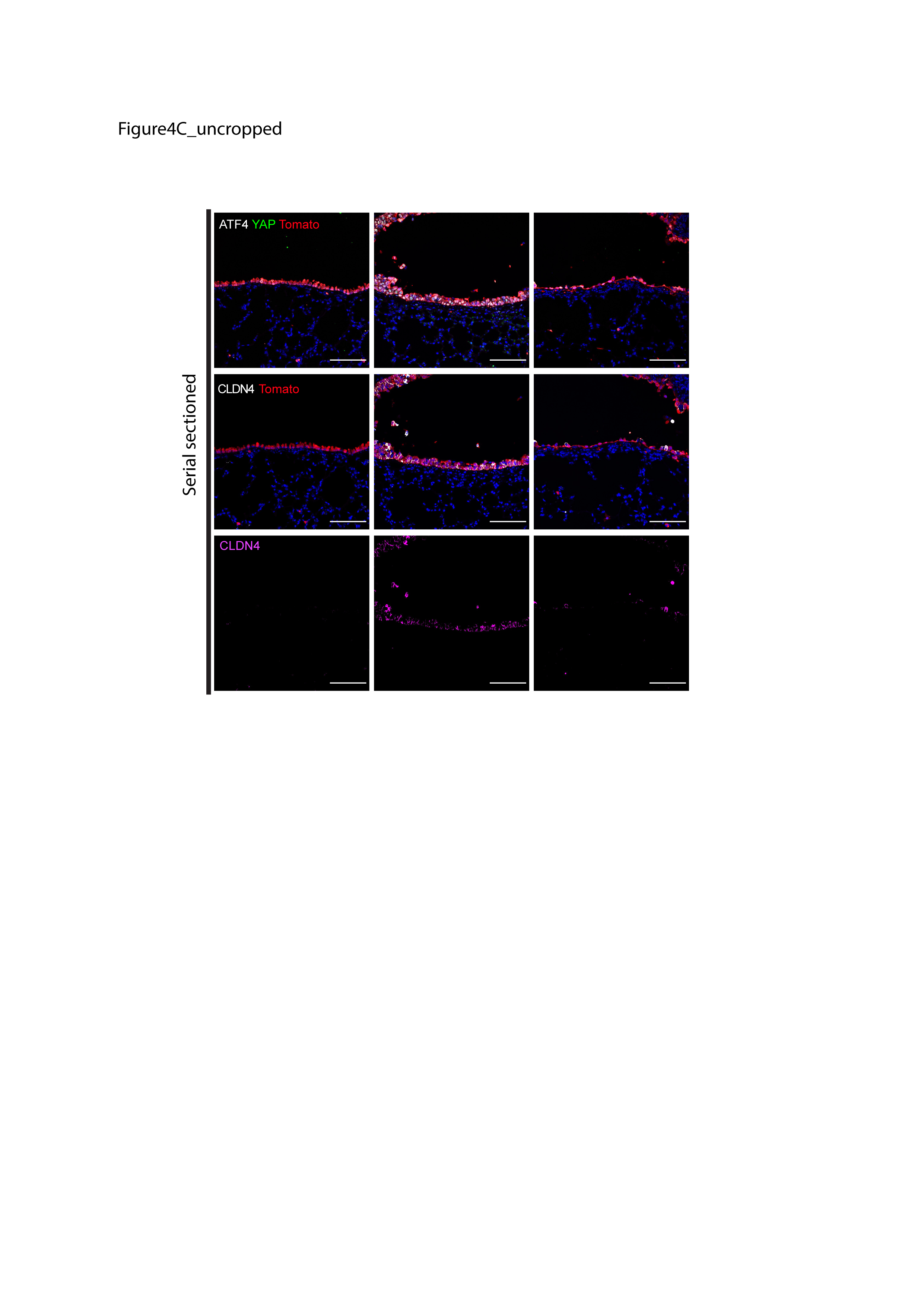

Supplement: Supplementary file 4 — Source Data for Figure 4 [file EMBJ-41-e109365-s002.zip › EMBOJ-2021-109365R1-Figure_4C_Source_Data-sd(2).tif]

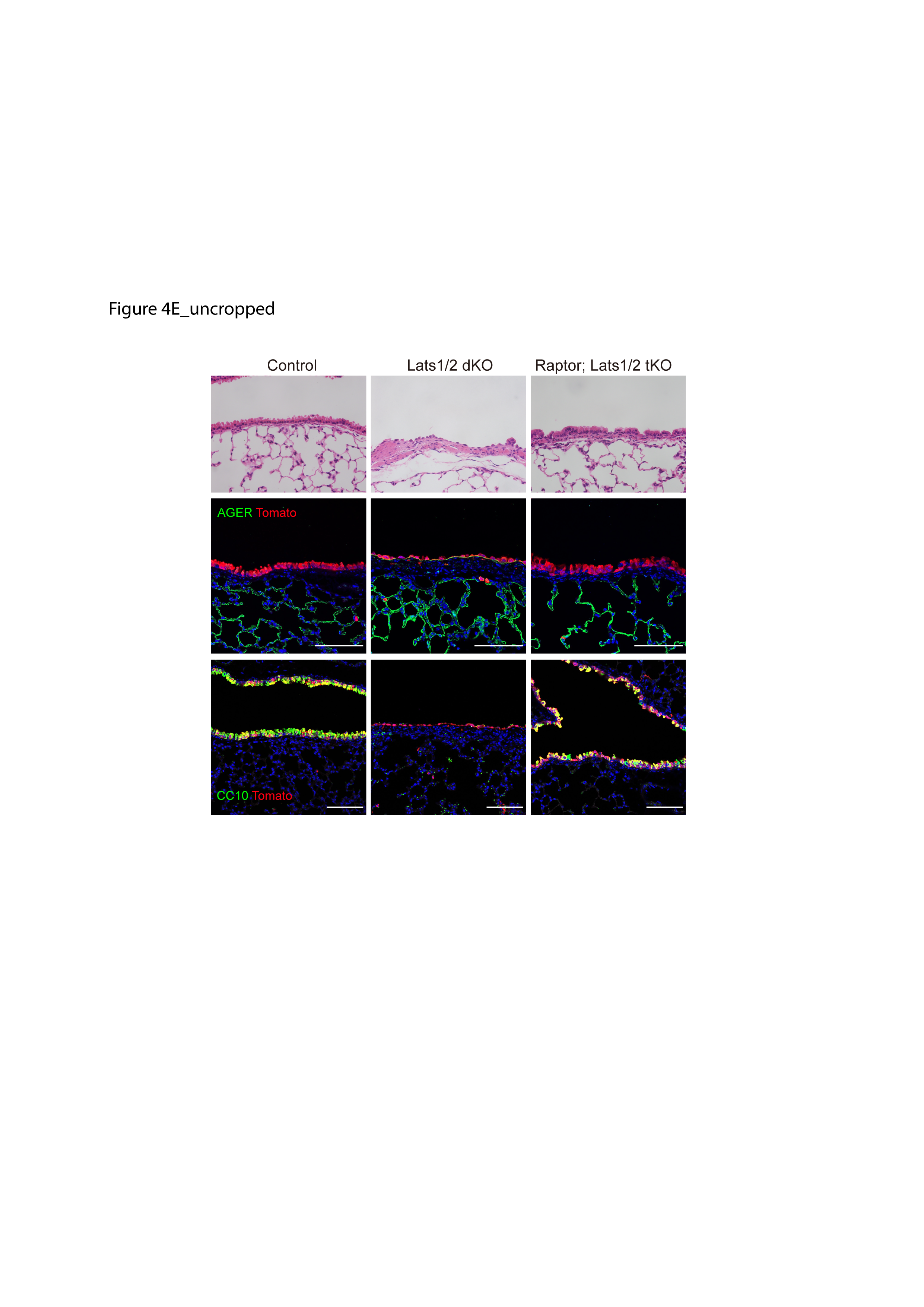

Supplement: Supplementary file 4 — Source Data for Figure 4 [file EMBJ-41-e109365-s002.zip › EMBOJ-2021-109365R1-Figure_4E_Source_Data-sd(2).tif]

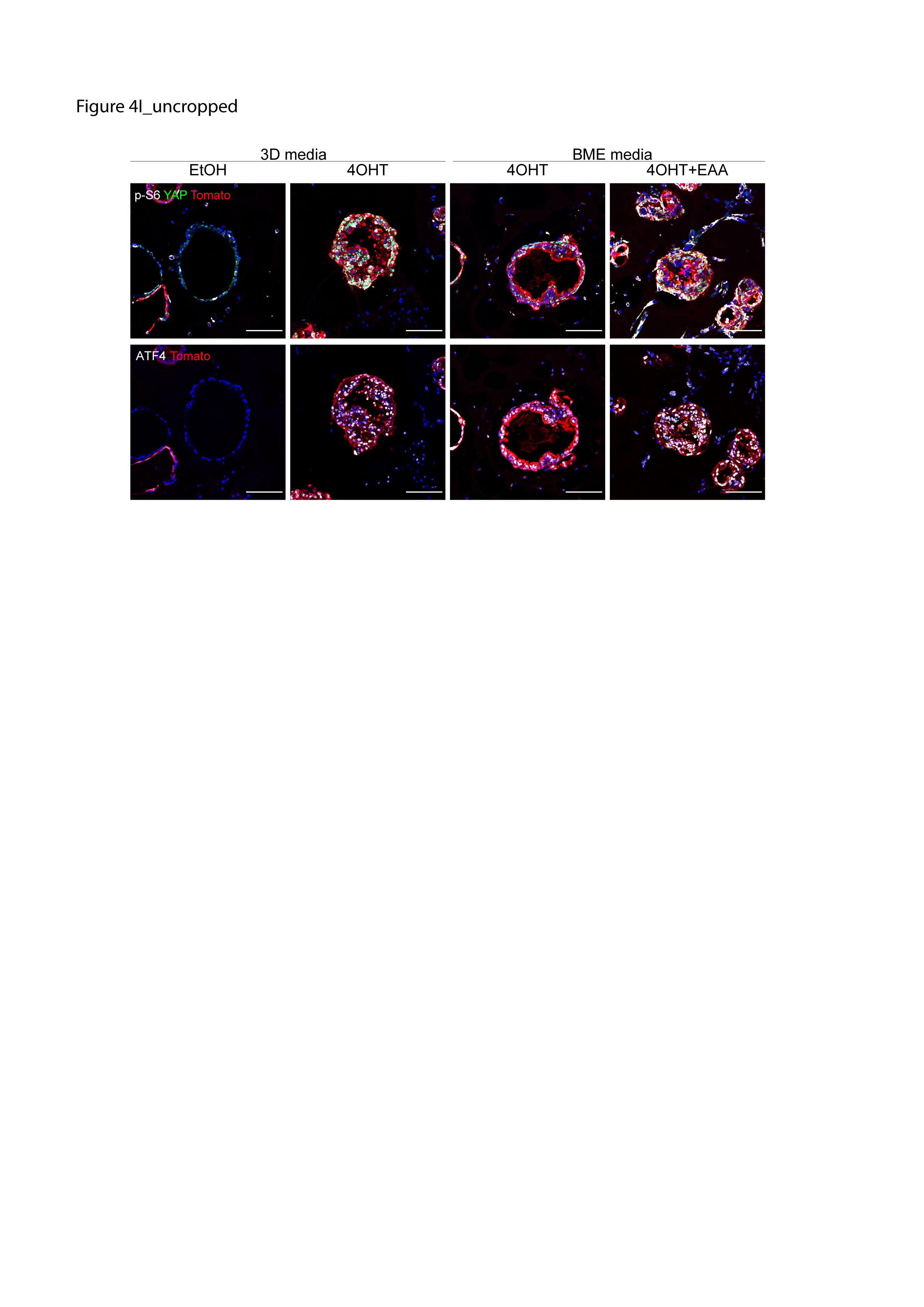

Supplement: Supplementary file 4 — Source Data for Figure 4 [file EMBJ-41-e109365-s002.zip › EMBOJ-2021-109365R1-Figure_4I_Source_Data-sd(1).tif]
