# Supplementary material for: Airway secretory cell fate conversion via YAP‐mTORC1‐dependent essential amino acid metabolism
Source: EMBO J. 2022 Mar 14;41(8):e109365. doi: 10.15252/embj.2021109365 (PMC9016350; doi:10.15252/embj.2021109365)
Supplement: Supplementary file 5 [file EMBJ-41-e109365-s004.pdf]

THE EMBO JOURNAL

Airway secretory cell fate conversion via YAP-mTORC1-dependent essential amino acid metabolism

Hae Yon Jeon, Jinwook Choi, Lianne Kraaier, Young Hoon Kim, David Eisenbarth, Kijong Yi, Ju-Gyeong Kang, Jin Woo Kim, Hyo Sup Shim, Joo-Hyeon Lee, and Dae-Sik Lim
DOI: 10.15252/embj.2021109365

Corresponding author: Dae-Sik Lim (daesiklim@kaist.ac.kr)

Review Timeline:

Submission Date:	3rd Aug 21
Editorial Decision:	23rd Sep 21
Revision Received:	5th Jan 22
Editorial Decision:	8th Feb 22
Revision Received:	17th Feb 22
Accepted:	17th Feb 22

Editor: Daniel Klimmeck

Transaction Report:

(Note: With the exception of the correction of typographical or spelling errors that could be a source of ambiguity, letters and reports are not edited. Depending on transfer agreements, referee reports obtained elsewhere may or may not be included in this compilation. Referee reports are anonymous unless the Referee chooses to sign their reports.)

1st Editorial Decision 23rd Sep 2021

Dear Dae-Sik, dear Joo-Hyeon,

Thank you again for the submission of your manuscript (EMBOJ-2021-109365) to The EMBO Journal. Please accept my apologies for the unusual delay with the peer-review of your work due to protracted referee input at this time of the year and detailed discussions in the team. Your study has been sent to three reviewers with complementary expertise on hippo developmental signaling (referee #1), lung cell biology (referee #2) and amino acid metabolism (referee #3) and we have received reports from all of them, which I enclose below.

As you will see, the referees acknowledge the potential interest of your results and in a timely context, although they also express major concerns, which need to be addressed before they can be supportive of publication at the EMBO Journal. In more detail, referee #2 states that s/he is not convinced of the physiological relevance of the observed YAP/TAZ-induced AT1-NAPT fate acquisition, and requests additional experimentation to corroborate this claim (ref#2, pts.1,2). Reviewer #3 points to concerns on the mechanistic depth presented for the induced TOR-ATF4-EAA axis and its impact on fate conversion and asks you to provide more details (ref#3, pts.1,4). Referee #1 requests a revised consideration of the discrepancies between dKO tissue versus organoid phenotypes (ref#1, pt.2). Finally, the referees list a number of additional issues related to nomenclature applied and technical controls related to the sample purity and potential confounding factors, that would need to be addressed to achieve the level of robustness needed for The EMBO Journal.

Given the referees' overall positive recommendations and detailed constructive comments, I would like to invite you to submit a revised version of the manuscript, addressing the issues raised. As it is EMBO Journal policy to allow only a single round of revision, acceptance of your manuscript will therefore depend on the completeness of your responses in this revised version.

In light of the extensive experimentation requested by the reviewers, I would appreciate if you could contact me during the next weeks via e.g. a video call to discuss your perspective on the comments and potential plan for revisions.

We generally allow three months as standard revision time. As a matter of policy, competing manuscripts published during this period will not negatively impact on our assessment of the conceptual advance presented by your study. However, we request that you contact the editor as soon as possible upon publication of any related work, to discuss how to proceed. Should you foresee a problem in meeting this three-month deadline, please let us know in advance and we may be able to grant an extension.

When submitting your revised manuscript, please carefully review the instructions below.

Thank you for the opportunity to consider your work for publication.
I look forward to your revision.

Best regards,

Daniel

Daniel Klimmeck, PhD
Senior Editor
The EMBO Journal

Instruction for the preparation of your revised manuscript:

1) a .docx formatted version of the manuscript text (including legends for main figures, EV figures and tables). Please make sure that the changes are highlighted to be clearly visible.

2) individual production quality figure files as .eps, .tif, .jpg (one file per figure).

3) a .docx formatted letter INCLUDING the reviewers' reports and your detailed point-by-point response to their comments. As part of the EMBO Press transparent editorial process, the point-by-point response is part of the Review Process File (RPF), which will be published alongside your paper.

4) a complete author checklist, which you can download from our author guidelines (https://wol-prod-cdn.literatumonline.com/pb-assets/embo-site/Author Checklist%20-%20EMBO%20J-1561436015657.xlsx). Please insert information in the checklist that is also reflected in the manuscript. The completed author checklist will also be part of the RPF.

5) Please note that all corresponding authors are required to supply an ORCID ID for their name upon submission of a revised

manuscript.

6) It is mandatory to include a 'Data Availability' section after the Materials and Methods. Before submitting your revision, primary datasets produced in this study need to be deposited in an appropriate public database, and the accession numbers and database listed under 'Data Availability'. Please remember to provide a reviewer password if the datasets are not yet public (see https://www.embopress.org/page/journal/14602075/authorguide#datadeposition).
In case you have no data that requires deposition in a public database, please state so in this section. Note that the Data Availability Section is restricted to new primary data that are part of this study.
*** Note - All links should resolve to a page where the data can be accessed. ***

7) Our journal encourages inclusion of *data citations in the reference list* to directly cite datasets that were re-used and obtained from public databases. Data citations in the article text are distinct from normal bibliographical citations and should directly link to the database records from which the data can be accessed. In the main text, data citations are formatted as follows: "Data ref: Smith et al, 2001" or "Data ref: NCBI Sequence Read Archive PRJNA342805, 2017". In the Reference list, data citations must be labeled with "[DATASET]". A data reference must provide the database name, accession number/identifiers and a resolvable link to the landing page from which the data can be accessed at the end of the reference. Further instructions are available at .

8) We would also encourage you to include the source data for figure panels that show essential data. Numerical data can be provided as individual .xls or .csv files (including a tab describing the data). For 'blots' or microscopy, uncropped images should be submitted (using a zip archive or a single pdf per main figure if multiple images need to be supplied for one panel). Additional information on source data and instruction on how to label the files are available at .

9) We replaced Supplementary Information with Expanded View (EV) Figures and Tables that are collapsible/expandable online (see examples in https://www.embopress.org/doi/10.15252/embj.201695874). A maximum of 5 EV Figures can be typeset. EV Figures should be cited as 'Figure EV1, Figure EV2" etc. in the text and their respective legends should be included in the main text after the legends of regular figures.

- For the figures that you do NOT wish to display as Expanded View figures, they should be bundled together with their legends in a single PDF file called *Appendix*, which should start with a short Table of Content. Appendix figures should be referred to in the main text as: "Appendix Figure S1, Appendix Figure S2" etc. See detailed instructions regarding expanded view here: .

- Additional Tables/Datasets should be labelled and referred to as Table EV1, Dataset EV1, etc. Legends have to be provided in a separate tab in case of .xls files. Alternatively, the legend can be supplied as a separate text file (README) and zipped together with the Table/Dataset file.

10) When assembling figures, please refer to our figure preparation guideline in order to ensure proper formatting and readability in print as well as on screen:
http://bit.ly/EMBOPressFigurePreparationGuideline

Please remember: Digital image enhancement is acceptable practice, as long as it accurately represents the original data and conforms to community standards. If a figure has been subjected to significant electronic manipulation, this must be noted in the figure legend or in the 'Materials and Methods' section. The editors reserve the right to request original versions of figures and the original images that were used to assemble the figure.

11) For data quantification: please specify the name of the statistical test used to generate error bars and P values, the number (n) of independent experiments (specify technical or biological replicates) underlying each data point and the test used to calculate p-values in each figure legend. The figure legends should contain a basic description of n, P and the test applied. Graphs must include a description of the bars and the error bars (s.d., s.e.m.).

Further information is available in our Guide to Authors: https://www.embopress.org/page/journal/14602075/authorguide

Revision to The EMBO Journal should be submitted online within 90 days, unless an extension has been requested and approved by the editor; please click on the link below to submit the revision online before 22nd Dec 2021:

https://emboj.msubmit.net/cgi-bin/main.plex

Referee #1:

This is an interesting manuscript. The phenotype of Lats1/2 dKO is carefully examined in the lung bronchial airway. In the Lats1/2 dKO, the columnar secretory cells lose their normal architecture and function, and are transformed into a flattened cell type that resembles alveolar AT1 cells. This fate conversion appears to involve TORC1 and ATF4. The human syndrome bronchiolitis obliterans appears similar to the Lats1/2 dKO phenotype.

Overall, the quantity and quality of data analysis are highly impressive, and the results will be of great interest to the Hippo signalling field, as well as to pulmonary science.

Minor comments

1. The human BO phenotype characterisation is important. Would the authors consider making this Figure 1 and mentioning it in the abstract, or even title of the manuscript?

2. It is interesting that the dKO organoids form multilayered epithelia, while in vivo the dKO forms a monolayer. Can the authors speculate as to why?

Referee #2:

In this manuscript, Jeon et al., used elegant genetic and pharmacological models to modulate Yap, mTOR and amino acid uptake mechanisms and claim that activation of Yap in airway secretory cells converts them to alveolar type-1 (AT1) cells via mTOR/amino acid import mechanisms. Specifically, the authors used genetic models to modulate Yap signaling in airway secretory cell and claim that activation of YAP in SCGB1A1+ airway secretory cells convert them into AT1-like cells in vivo and in ex vivo organotypic cultures. Additionally, the authors state that this plasticity involves a recently identified DAPT state through which secretory cells transition to AT1 cells. The authors also claim that YAP-mediated essential amino acid influx controls secretory cell plasticity via mTOR signaling. Finally, the authors claim that similar mechanisms are at play in human lung diseases including pulmonary fibrosis and bronchiolitis obliterans.

In summary, while the mechanisms described here are of potential importance to our understanding of airway cell plasticity, as I detailed below, the observations made here falls short of physiological relevance. Additionally, the data provided here does not support some of the claims (see below). Addressing the following comments would make this manuscript more meaningful and support the claims in this manuscript.

Major comments:

1. The authors show that activation of YAP/TAZ is sufficient to convert airway secretory cells into AT1 cells. However, plasticity observed here is somewhat "artificial" as the current data does not show whether the same mechanisms are involved in injury repair. Recent studies (Strunz et al., 2020) have indicated that Sox2-lineage labeled cells can generate AT2 and AT1 cells after bleomycin injury. Therefore, this reviewer questions the relevance of this finding to injury repair!
2. The secretory cell derived AT1 cells are located in airways, so, they may not have physiological relevance as these cells need to be connected to the vasculature for gas exchange. So, the question is whether there is micro-capillary associated with these AT1 cells? Additionally, prior studies have shown that airway secretory cells can migrate to alveolar regions after damage (ex: flu infection). So, how about activating Yap in secretory cells once they migrate to alveoli after injury (ex: flu infection) to test whether these cells can generate AT1? I suggest the author test this as this will reveal the physiological relevance of secretory cell derived AT1 cells.
3. The authors used the term DATPs. However, the plasticity observed here is due to genetic inactivation of Lats1/2 (and not due to injury/damage alone). It is more appropriate to use reprogrammed cells or Yap-induced transient state.
4. The authors used tdTomato+ cells from Scgb1a1-creER/R26-tdtomato mice for scRNA-sequencing (Fig. 2 and EV3). However, there are numerous immune, fibroblast and mesothelial cells. This suggests that there are contaminating cells and that might obscure some of the findings (ex: organoid cultures). The authors need to provide additional data to support cell purity or repeat these experiments with pure cell populations.
5. Day-21 Images in Fig-2G' and Fig. EV2E: It appears that the authors used the same images (red and green channels) in both figures. Although this does not affect the conclusions in this manuscript, this might be flagged as "image duplication".
6. In Fig-3F, there are some tdTomato+ cells in EtOH treated condition (top right corner). Is that due to sporadic activation of cre recombinase or is this mouse line leaky?
7. Generally, AT1 cells are localized to luminal side in most organoid cultures (Ex: Barkauskas et al., JCI 2013). However, in Fig-3D', AT1 markers are localized to basal side. Therefore, it is unclear whether the AT1-like cells that the authors observed in these models truly represent resident AT1 cells? Therefore, a comprehensive analysis of AT1-like cells in in vivo and ex vivo models and a direct comparison with resident AT1 cells is required to check the validity of the conclusions.
8. In Fig. 5 and EV6, the authors claim that AT1-like cells are observed in airways of pulmonary fibrosis and bronchiolitis Obliterans lungs. However, the marker used here (AQP5) is not specific to AT1 cells. AQP5 is known to express in airway epithelial cells. Therefore, additional markers such as AGER and PDPN are necessary to support this claim. Additionally, the

cells do not appear to show flat and thin morphology as expected for AT1 cells. So, this reviewer doubts the conclusion that they are AT1 cells.
9. It is unclear what the authors are trying to show from the data in Fig. EV6A-C? There is no indication that neither AT1 nor DATP cells have originated from secretory cells. Again, the authors chose to label cluster #4 as DATPs. However, the original study (Haberman et al., Science Advances, 2020) have termed them basaloid cells. Altogether, the current data does not support whether de novo conversion of secretory cells into AT1 cells occurs in these diseases.
10. The authors claim that they identified KDR as a marker of secretory cells. There are other prior studies identified KDR as a marker and they (ex: Jiang et al., Developmental Cell, 2021) should be cited here.

Minor:
- In Fig-1B and D, it would be easier for the authors if the panels clearly show the mouse genotype.

Referee #3:

Summary:

The manuscript by Jeon et al., titled "Airway secretory cell fate conversion via YAP-mTORC1-dependent essential amino acid metabolism" offers some mechanistic understanding of a differentiation program in airway secretory cells, implicating YAP/TAZ signaling and downstream engagement and requirement of mTORC1-ATF4-mediated essential amino acid uptake. Although overall the findings are interesting, the study is well-conceptualized and thoughtfully designed, with well-controlled experiments, additional experimental work is needed to substantiate the conclusions in regard to the involvement/role of mTORC1-mediated ATF4 upregulation and the consequent increase in the expression of SLC7A5 and essential amino acid uptake. Essentially, authors need to link (1) YAP/TAZ signaling with mTORC1 (at least experimentally implicate previously known link), (2) mTORC1 signaling with ATF4, and (3) essential amino acid uptake with the conversion of the airway secretory cells into AT1 cells.

Specific Comments:

1) It remains to be determined how essential amino acid uptake is facilitating the transition of secretory cells into DATP-AT1 cells. This is an important question which authors should address. Another question that needs to answered is whether there is/are few specific essential amino acids that are responsible for the phenotype change observed here. Authors should consider demonstrating an actual increase in the uptake of essential amino acids (which may also help answer the second question above) as cells transition from secretory phenotype to DATP/AT1 phenotype. Moreover, the impact of mTORC1 inhibition on essential amino acid uptake in LATS1/2 dKO cells should be studied.

2) ATF4 expression is known to be pre-dominantly regulated at the translational level, owing to increased phosphorylation of eIF2-alpha. Did authors evaluate a possible change in ATF4 protein levels and p-eIF2-alpha levels (by western blotting)?

3) Authors should demonstrate the role of ATF4 in the upregulation of the amino acid transporter SLC7A5 and the consequent increased uptake of essential amino acids.

4) It remains unclear how the activation of YAP/TAZ signaling is increasing mTORC1 activity and consequently ATF4 transcription. Essential amino acids are known positive modulators of mTORC1 activity. Is it conceivable that ATF4-mediated upregulation of SLC7A5 precedes and facilitates essential amino acid uptake, which in turn enhances mTORC1 activity? It may be an important avenue to look into in order to develop a better understanding of the mechanistic underpinnings of the observations.

5) How do authors rule out that suppression of ATF4 in Raptor deleted cells is not simply due to compromised mRNA translation, owing to impaired mTORC1 activity?

Response to the Reviewers' comments

We are grateful for the reviewers' valuable comments, which helped us strengthen our manuscript. We have comprehensively addressed all concerns with additional experiments, which we believe in providing stronger and clearer support for our conclusions.

Referee #1:

This is an interesting manuscript. The phenotype of Lats1/2 dKO is carefully examined in the lung bronchial airway. In the Lats1/2 dKO, the columnar secretory cells lose their normal architecture and function, and are transformed into a flattened cell type that resembles alveolar AT1 cells. This fate conversion appears to involve TORC1 and ATF4. The human syndrome bronchiolitis obliterans appears similar to the Lats1/2 dKO phenotype.

Overall, the quantity and quality of data analysis are highly impressive, and the results will be of great interest to the Hippo signalling field, as well as to pulmonary science.

Response: We thank the Reviewer for highlighting the novelty of our findings that advance the fields.

Minor comments

1. The human BO phenotype characterisation is important. Would the authors consider making this Figure 1 and mentioning it in the abstract, or even title of the manuscript?

Response: Following the reviewer's suggestion, we have amended the abstract by highlighting the physiological relevance of our finding in BO phenotypes in our revised manuscript (In abstract, page; 2, line; 10-13).

2. It is interesting that the dKO organoids form multilayered epithelia, while in vivo the dKO forms a monolayer. Can the authors speculate as to why?

Response: As shown in Fig. 1B, D and Fig. 2G, H, we observed multi-layered epithelium at day 5 post Lats1/2 deletion. It is likely that YAP/TAZ activation at the early phase before converting into DATP cell states enhanced the proliferation of secretory cells. However, some of them, except differentiated DATPs and AT1 cells, detached off, leaving the epithelium monolayered. In contrast, secretory and differentiated cells in 3D organoids do not seem to detach significantly, possibly due to the different microenvironments such as the lack of mucociliary airway clearance *in vitro*.

We thank the Reviewer for all the constructive and helpful suggestions.

Referee #2:

In this manuscript, Jeon et al., used elegant genetic and pharmacological models to modulate Yap, mTOR and amino acid uptake mechanisms and claim that activation of Yap in airway secretory cells converts them to alveolar type-1 (AT1) cells via mTOR/amino acid import mechanisms. Specifically, the authors used genetic models to modulate Yap signaling in airway secretory cell and claim that activation of YAP in SCGB1A1+ airway secretory cells convert them into AT1-like cells in vivo and in ex vivo organotypic cultures. Additionally, the authors state that this plasticity involves a recently identified DATP state through which secretory cells transition to AT1 cells. The authors also claim that YAP-mediated essential amino acid influx controls secretory cell plasticity via mTOR signaling. Finally, the authors claim that similar mechanisms are at play in human lung diseases including pulmonary fibrosis and bronchiolitis obliterans.

In summary, while the mechanisms described here are of potential importance to our understanding of airway cell plasticity, as I detailed below, the observations made here falls short of physiological relevance. Additionally, the data provided here does not support some of the claims (see below). Addressing the following comments would make this manuscript more meaningful and support the claims in this manuscript.

Major comments:

1. The authors show that activation of YAP/TAZ is sufficient to convert airway secretory cells into AT1 cells. However, plasticity observed here is somewhat "artificial" as the current data does not show whether the same mechanisms are involved in injury repair. Recent studies (Strunz et al., 2020) have indicated that Sox2-lineage labeled cells can generate AT2 and AT1 cells after bleomycin injury. Therefore, this reviewer questions the relevance of this finding to injury repair!

Response: We fully agree with the Reviewer's critical comment related to the physiological relevance of our findings to injury repair. Interestingly, we observed a transient YAP/TAZ activation in lineage-labeled secretory cells of *Scgb1a1-CreER*$^{TM/+}$*;R26R*$^{tdTomato/+}$ mice during airway injury repair in response to naphthalene treatment. At day 5 post-injury, increased nuclear localization of YAP/TAZ was detected in lineage-labeled cells, which lost CC10 expression and acquired the expression of DATP marker CLDN4 (Revised Fig. EV5A, B). We also observed the expression of phospho-S6 and ATF4 in these cells, which was then declined with the regeneration of secretory cells (Revised Fig. EV5A, B). There was no AT1 cell differentiation on this occasion. These results suggest the potential role of YAP/TAZ activation induced by injury in modulating an emergence of DATPs with mTORC1/ATF4 activity during airway injury repair. Significantly, we observed *Scgb1a1*$^{+}$ lineage-labeled AT1 cells in the airway epithelium post chronic airway injury by repetitive naphthalene treatment (Revised Fig. EV5C, D). Sustained YAP/TAZ activation induced by chronic damage likely promotes fate conversion of secretory cells into AT1 cells, relevant to chronic lung diseases such as BO and IPF. Future study needs to be elucidated to understand the cellular function of DATPs emerging during acute injury repair in the airway. It would also be important to understand how the sustained YAP/TAZ activation induced by chronic injury results in the altered fate conversion of secretory cells into AT1 fate while there is no visible abnormal change in the acute injury model. We have now included these new data and discussed the physiological relevance of YAP/TAZ activation in the discussion section of our revised manuscript (page:13, line; 11-21)

2. The secretory cell derived AT1 cells are located in airways, so, they may not have physiological relevance as these cells need to be connected to the vasculature for gas exchange. So, the question is whether there is micro-capillary associated with these AT1 cells? Additionally, prior studies have shown that airway secretory cells can migrate to alveolar

regions after damage (ex: flu infection). So, how about activating Yap in secretory cells once they migrate to alveoli after injury (ex: flu infection) to test whether these cells can generate AT1? I suggest the author test this as this will reveal the physiological relevance of secretory cell derived AT1 cells.

Response: We thank the reviewer for these critical comments. As suggested, we have carefully checked the vascular cells around AT1 cells derived from Lats1/2-deficient secretory cells. We found the microvascular structure expressing endothelial marker VECAM adjacent to lineage-labeled AT1 cells in the airways, suggesting that YAP/TAZ activation converted secretory cells into bona-fide AT1 cells retaining the potential for gas-exchange with capillary endothelial cells (Revised Fig. EV1E).

It would be interesting to test whether YAP-activated secretory cells can migrate and differentiate into AT1 cells in the alveoli post-injury. However, as we have shown, Lats1/2-deficient secretory cells quickly transited into DATPs and AT1 cells at day 5 post tamoxifen treatment. They caused severe pulmonary fibrosis with the compromised epithelial integrity in the airways. Thus, we were unable to deliver a further injury to these animals due to the health concerns. Also, conducting the flu infection experiment requires special facilities and permission, but we did not have both facilities and permission.
Instead, we have transplanted *Scgb1a1*$^{+}$ lineage-labeled secretory cells isolated from Lats1/2 dKO lungs into bleomycin-injured WT lungs via intratracheal administration (Rev_Figure 1). Importantly, we observed lineage-labeled AT1 cells in the alveoli, indicating the differentiation ability of YAP/TAZ-activating secretory cells into AT1 cells.

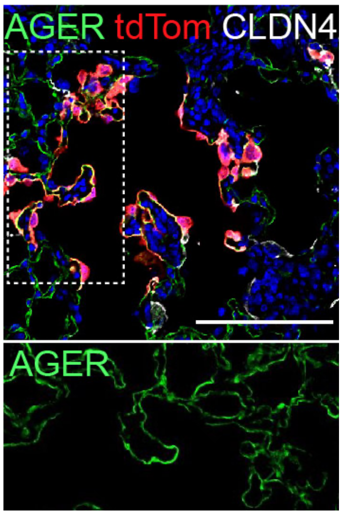

Rev_Figure 1. **Differentiation potential of YAP/TAZ-activating secretory cells into AT1 cells**. Representative immunofluorescent (IF) images showing lineage-labeled AT1 cells in alveolar regions at day 14 post transplantation: AGER (green), tdTom (red), CLDN4 (white), and DAPI (blue). Scale bar, 100μm.

3. The authors used the term DATPs. However, the plasticity observed here is due to genetic inactivation of Lats1/2 (and not due to injury/damage alone). It is more appropriate to use reprogrammed cells or Yap-induced transient state.

Response: We used the terminology of DATP as the intermediate cell state emerging by YAP/TAZ activation from secretory cells before AT1 cell differentiation showed the transcriptional signatures significantly shared with DATPs emerging during injury repair. Thus, we want to use the term of DATPs in this paper.

4. The authors used tdTomato+ cells from Scgb1a1-creER/R26-tdtomato mice for scRNA-sequencing (Fig. 2 and EV3). However, there are numerous immune, fibroblast and mesothelial cells. This suggests that there are contaminating cells and that might obscure some of the

findings (ex: organoid cultures). The authors need to provide additional data to support cell purity or repeat these experiments with pure cell populations.

Response: As the Reviewer pointed out, cells acquired for scRNA-seq analysis showed some contamination of non-epithelial cells. So, we removed these clusters for analysis as there is no tdTomato expression in immune cells, fibroblasts, and mesothelial cells. Additionally, we have performed cytospin staining to verify the cellular composition in the population of tdTomato$^+$ cells isolated from *Scgb1a1-CreER*$^{TM/+}$;*R26R*$^{tdTomato/+}$ mice before further experiments including organoid cultures. We have confirmed that CC10$^+$ secretory cells were mostly enriched in the population of tdTomato$^+$ cells (Rev_Figure 2).

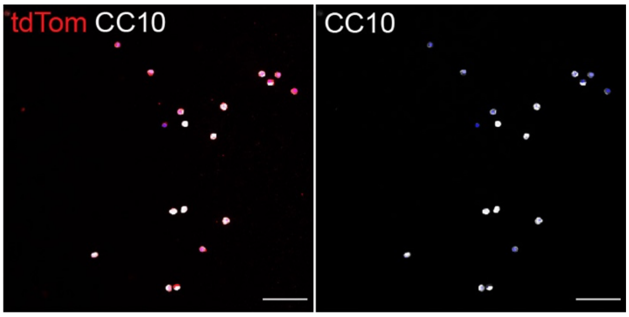

Rev_Figure 2. **Enrichment of secretory cells in tdTomato+ lineage-labeled cells.** Tomato$^+$ cells isolated from *Scgb1a1-CreER*$^{TM/+}$;*R26R*$^{tdTomato/+}$ lungs retain secretory cells. Representative IF images of cytospin staining from lineage-labeled cells isolated from *Scgb1a1-CreER*$^{TM/+}$;*R26R*$^{tdTomato/+}$ mice. CC10 (white), tdTom (red), and DAPI (blue). Scale bar, 100µm.

5. Day-21 Images in Fig-2G' and Fig. EV2E: It appears that the authors used the same images (red and green channels) in both figures. Although this does not affect the conclusions in this manuscript, this might be flagged as "image duplication".

Response: We apologize for the mis-organized figures. We have carefully checked and amended typos in our revised manuscript.

6. In Fig-3F, there are some tdTomato+ cells in EtOH treated condition (top right corner). Is that due to sporadic activation of cre recombinase or is this mouse line leaky?

Response: As the Reviewer pointed out, we detected the low incidence of labeled secretory cells in corn oil-treated *Scgb1a1-CreER*$^{TM/+}$; *R26R*$^{fGFP/+}$ mouse lungs, consistent with an original paper generating this mouse model (Rawlins et al., 2009) (Rev_Figure 3). In order to minimize this effect, we used 4-6 weeks-old male mice, which display minimum leakiness.

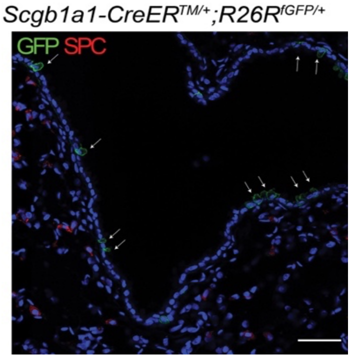

Rev_Figure 3. **Validation of the leakiness of reporter mouse model.** Representative IF images showing reporter expression after corn oil treatment in the indicated genotype: GFP (green), SPC (red), and DAPI (blue). Arrows point to report-positive cells. Scale bars, 50µm.

7. Generally, AT1 cells are localized to luminal side in most organoid cultures (Ex: Barkauskas et al., JCI 2013). However, in Fig-3D', AT1 markers are localized to basal side. Therefore, it is unclear whether the AT1-like cells that the authors observed in these models truly represent resident AT1 cells? Therefore, a comprehensive analysis of AT1-like cells in in vivo and ex vivo models and a direct comparison with resident AT1 cells is required to check the validity of the conclusions.

Response: As pointed out, AT1 cells retained in organoids derived from AT2 cells are localized luminal side (inner part) of organoids because AT2 cells can self-renew and also give rise to AT1 cells. However, in our study, YAP/TAZ activation in the secretory cells promotes the cellular conversion of secretory cells into AT1 cell fate via DATP cell states. Thus, AT1 cells were detected in the basal side of organoids derived from Lats1/2-deficient secretory cells. Furthermore, as the Reviewer suggested, we have confirmed the emergence of vasculature adjacent to lineage-labeled AT1 cells derived from Lats1/2-deficient secretory cells in the airways (Revised Fig. EV1E), and the differentiation potential of Lats1/2-deficient secretory cells into AT1 cells in the alveoli (Rev_Figure 1). These data support that AT1 cells derived from Lats1/2-deficient secretory cells are a bona-fide AT1 cell in our study with *in vivo* and *ex vivo* model.

8. In Fig. 5 and EV6, the authors claim that AT1-like cells are observed in airways of pulmonary fibrosis and bronchiolitis Obliterans lungs. However, the marker used here (AQP5) is not specific to AT1 cells. AQP5 is known to express in airway epithelial cells. Therefore, additional markers such as AGER and PDPN are necessary to support this claim. Additionally, the **cells do not appear to show flat and thin morphology as expected for AT1 cells**. So, this reviewer doubts the conclusion that they are AT1 cells.

Response: To prove the presence of AT1 cells in the airways, we also used another AT1 cell marker, Caveolin-1 (CAV1), in addition to AQP5 (Revised Fig. EV4A). Regarding the morphology of AT1 cells in BO tissues, we further observed monolayered thin epithelium retaining mostly AT1 cells and replaced the images in Fig. 6 and Fig. EV4 of our revised manuscript.

9. It is unclear what the authors are trying to show from the data in Fig. EV6A-C? There is no indication that neither AT1 nor DATP cells have originated from secretory cells. Again, the authors chose to label cluster #4 as DATPs. However, the original study (Haberman et al., Science Advances, 2020) have termed them basaloid cells. Altogether, the current data does not support whether de novo conversion of secretory cells into AT1 cells occurs in these diseases.

Response: Recent other studies, including ours (Choi et al., 2020; Kobayashi et al., 2020; Strunz et al., 2020), alongside Haberman et al., identified KRT8^{high} population (named as ADI, PATS, DATP) that show similar transcriptional signatures of Basaloid found in human IPF sample (Habermann et al., 2020). Thus, we named this population following those recent studies.

10. The authors claim that they identified KDR as a marker of secretory cells. There are other prior studies identified KDR as a marker and they (ex: Jiang et al., Developmental Cell, 2021) should be cited here.

Response: We have cited this paper in our revised manuscript.

Minor:
- In Fig-1B and D, it would be easier for the authors if the panels clearly show the mouse genotype.

Response: We have amended those figures following the reviewer's suggestion in our revised manuscript.

We thank the Reviewer for all the constructive and helpful suggestions.

Referee #3:

Summary:

The manuscript by Jeon et al., titled "Airway secretory cell fate conversion via YAP-mTORC1-dependent essential amino acid metabolism" offers some mechanistic understanding of a differentiation program in airway secretory cells, implicating YAP/TAZ signaling and downstream engagement and requirement of mTORC1-ATF4-mediated essential amino acid uptake. Although overall the findings are interesting, the study is well-conceptualized and thoughtfully designed, with well-controlled experiments, additional experimental work is needed to substantiate the conclusions in regard to the involvement/role of mTORC1-mediated ATF4 upregulation and the **consequent increase in the expression of SLC7A5 and essential amino acid uptake**. Essentially, authors need to link (1) YAP/TAZ signaling with mTORC1 (at least experimentally implicate previously known link), (2) mTORC1 signaling with ATF4, and (3) essential amino acid uptake with the conversion of the airway secretory cells into AT1 cells.

Specific Comments:

1) It remains to be determined **how essential amino acid uptake is facilitating the transition of secretory cells into DATP-AT1 cells**. This is an important question which authors should address. Another question that needs to answered is whether there is/are **few specific essential amino acids that are responsible for the phenotype change** observed here. Authors should consider demonstrating an actual increase in the uptake of essential amino acids (which may also help answer the second question above) as cells transition from secretory phenotype to DATP/AT1 phenotype. Moreover, the impact of mTORC1 inhibition on essential amino acid uptake in LATS1/2 dKO cells should be studied.

Response: We thank the Reviewer's valuable comments. Our study focused on defining how persistent YAP/TAZ activation caused altered cell fate conversion of airway secretory cells into squamous AT1 fate in the airways via mTORC1-ATF4 activity and its implication in human lung diseases such as BO and IPF. We further identified that EAA uptake via Slc7a5 is crucial for this fate transition via DATP cell state known to emerge during injury repair.
We fully agree that determining how EAA uptake promotes this cellular change would be an important question in our future study. Our recent study showed that glycolytic metabolism is a key driver for cell fate transition into DATPs retaining the capacity to convert into AT1 cells (Choi et al., 2020). It has been also suggested that Slc7a5-dependent EAA uptake is required for glycolysis metabolism (Yoon et al., 2018; Yue et al., 2017). Thus, it is likely that metabolic realignment into glycolysis, mediated by EAA uptake, seems to be critical for fate decision of secretory cells. We will test this hypothesis in the future.

We have extensively tried to further narrow down the candidate EAA critical for fate conversion of secretory cells. Nine EAA (Histidine, Isoleucine, Leucine, Methionine, Threonine, Valine, Glutamine, Arginine, Cysteine) were tested in our study. As shown in Rev_Figure 4, the addition of branched chain amino acids (BCAA; Isoleucine, Leucine, Valine) in AA-limited BME media was not sufficient to promote fate conversion of secretory cells into DATPs. However, we observed the emergence of DATPs in organoids treated with conditional EAA (Arginine, Cysteine, Glutamine, Glycine, Proline, Tyrosine). Thus, it is likely that 4 amino acids (Cysteine, Arginine, Glutamine, and Glycine) seem to be essential components to drive the fate conversion of secretory cells into DATP/AT1 cells, which is regulated by YAP/TAZ activation. Further investigation would be interesting to study how these specific EAAs are regulated in this process in the future.

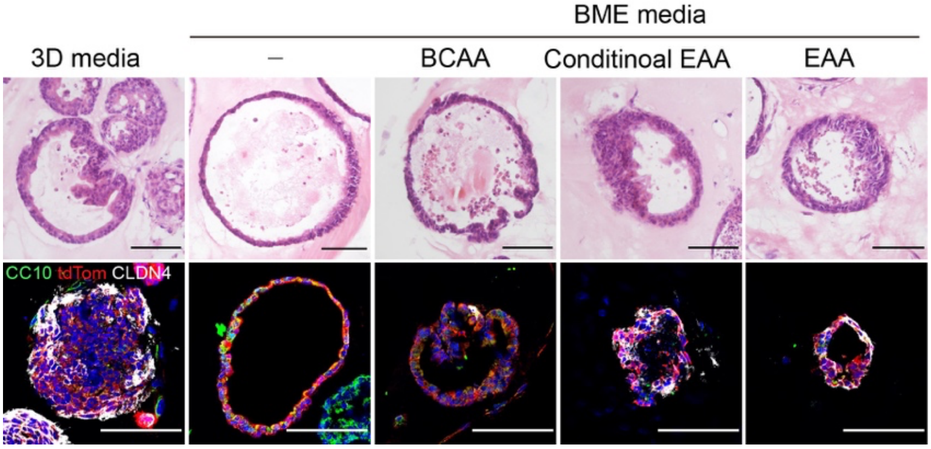

***Rev_Figure 4*. Fate conversion of secretory cells into DATPs via amino acid uptake.** Representative H&E (top) and IF (bottom) images of secretory, DATP, or AT1 cells in organoids derived from Lats1/2-deficient secretory cells in indicated culture conditions. Tomato (for Scgb1a1 lineage, red), CC10 (for secretory cells, green), CLDN4 (for DATPs, white), and DAPI (blue). Scale bars, 100µm.

As suggested, we measured the actual increase of amino acid uptake in the cellular transition by isolating lineage-labeled cells from control, Lats1/2 dKO, and Lats1/2;Raptor tKO lungs at day 5 post tamoxifen treatment. We observed the increased uptake of amino acids in Lats1/2 dKO cells where secretory cells transited into DATPs/AT1 cells (Revised Fig. 4H). Importantly, deletion of Raptor significantly inhibited the amino acid uptake by YAP/TAZ activation, in accordance with inhibiting cellular transitions of secretory cells into DATPs/AT1 cells.

2) ATF4 expression is known to be predominantly regulated at the translational level, owing to increased phosphorylation of eIF2-alpha. Did authors evaluate a possible change in ATF4 protein levels and p-eIF2-alpha levels (by western blotting)?

Response: As there are a limited number of secretory cells in the lungs, western blotting is not available in our system. Instead, we have already detected increased protein levels of nuclear ATF4 expression both in organoids and lung tissues from mice and humans using immunostaining analysis in our manuscript. As suggested, we also examined the dynamic change of p-eIF2-alpha in protein levels using IF staining. As shown in Rev_Figure 5, there was no significant upregulation of p-eIF2-alpha expression in the airways during cell fate transition by YAP/TAZ activation.

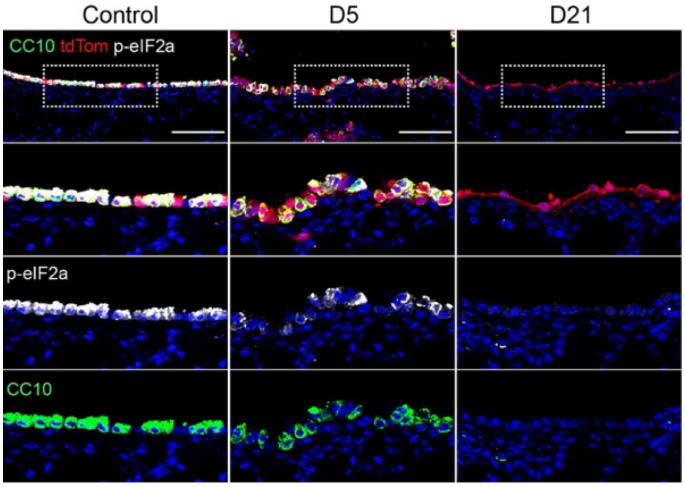

***Rev_Figure 5*. Expression levels of p-eIF2-alpha during fate conversion of secretory cells by YAP/TAZ activation.** Representative IF images of control and Lats1/2 dKO airways at indicated time points post tamoxifen treatment. Tomato (for Scgb1a1 lineage, red), CC10 (for secretory cell, green), p-eIF2-alpha (white), and DAPI (blue). Scale bar, 100µm.

3) Authors should demonstrate the role of ATF4 in the upregulation of the amino acid transporter SLC7A5 and the consequent increased uptake of essential amino acids.

Response: As suggested, we examined the effect of ATF4 in EAA uptake and fate decision of secretory cells and included these results in our revised manuscript (Revised Fig. 5). Consistent with Lats1/2 dKO lungs, constitutive activation of YAP signalling by overexpressing YAP 5SA mutant in the airway cell line enhanced the EAA uptake with increased expression of ATF4 and its target genes including Slc7a5 (Revised Fig. 5A-C). However, knockdown (KD) of ATF4 caused the defects in EAA uptake with reduced expression of Slc7a5 (Revised Fig. 5A, C). Furthermore, ATF4 KD in organoids derived from *Lats1/2*-deficient secretory cells impaired the fate conversion of secretory cells into DATPs and AT1 cells (Revised Fig. 5D-F). We also confirmed that Slc7a5 KD in Lats1/2-deficient secretory organoids inhibited their transition into DATPs and AT1 cells (Revised Fig. 5D-F). In contrast, sustained overexpression of ATF4 significantly promoted the fate conversion of secretory cells into DATPs and AT1 cells (Revised Fig. 5G-I). These results strongly support the functional role of ATF4 in upregulating SLC7A5 levels, allowing EAA uptake.

4) It remains unclear how the activation of YAP/TAZ signaling is increasing mTORC1 activity and consequently ATF4 transcription. Essential amino acids are known positive modulators of mTORC1 activity. **Is it conceivable that ATF4-mediated upregulation of SLC7A5 precedes and facilitates essential amino acid uptake, which in turn enhances mTORC1 activity?** It may be an important avenue to look into in order to develop a better understanding of the mechanistic underpinnings of the observations.

Response: We agree with the Reviewer's critical view that it is important to determine the molecular mechanisms of EAA and YAP-mTOR-ATF4 circuit in cell fate conversion. YAP/TAZ has been shown to directly and/or indirectly influence mTORC1 activity (Tumaneng et al., 2012; Hansen et al., 2015; Hu et al., 2017). As pointed out, we cannot completely rule out the possibility that enhanced mTORC1 activity by YAP/TAZ activation results from upregulation of EAA uptake by ATF4-dependent Slc7a5 expression. However, as shown in Revised Fig. 4I, organoids derived from Lats1/2-deficient secretory cells showed that the expression level of phospho-S6 was not significantly affected by EAA depletion (4OHT+BME media) compared to control (4OHT+3D media). Thus, it is likely that the upregulation of mTORC1 activity in Lats1/2-deficient secretory cells seems to be directly affected by YAP/TAZ activation. However, we observed the augmented activity of mTORC1 activity by addition of EAA (4OHT+EAA+BME media) compared to organoids cultured in limited amino acid (4OHT+BME media), which indicating that there is a feedforward positive feedback loop by EAA on mTORC1 activity. We hope that the Reviewer understands the challenge to show direct evidence that ATF4-mediated amino acid uptake comes before mTORC1 activity due to this positive feedback loop.

5) **How do authors rule out that suppression of ATF4 in Raptor deleted cells is not simply due to compromised mRNA translation**, owing to impaired mTORC1 activity?

Response: As the Reviewer pointed out, inhibition of mTORC1 by Raptor deletion in Lats1/2 dKO mice can impact global mRNA translational activity. Thus, we cannot rule out the possibility 100%. However, the deletion of Raptor enhanced the upregulation of CC10 expression in the secretory cells of Lats1/2 dKO mice alongside the reduction of ATF4 and other DATP-marker genes. Also, ATF4 KD in *Lats1/2*-deficient organoids impaired the fate conversion of secretory cells into DATPs and AT1 cells (Revised Fig. 5D-F), suggesting the critical role of ATF4 in this cell conversion. Thus, it is unlikely that the rescue of cell fate conversion by mTORC1 inhibition is simply due to the overall translational halt. We hope that this explanation satisfies the Reviewer.

Finally, we thank the Reviewer for all the constructive and helpful suggestions.

Reference

Choi, J., Park, J.E., Tsagkogeorga, G., Yanagita, M., Koo, B.K., Han, N., and Lee, J.H. (2020). Inflammatory Signals Induce AT2 Cell-Derived Damage-Associated Transient Progenitors that Mediate Alveolar Regeneration. Cell Stem Cell *27*, 366-382 e367.

Habermann, A.C., Gutierrez, A.J., Bui, L.T., Yahn, S.L., Winters, N.I., Calvi, C.L., Peter, L., Chung, M.I., Taylor, C.J., Jetter, C.*, et al.* (2020). Single-cell RNA sequencing reveals profibrotic roles of distinct epithelial and mesenchymal lineages in pulmonary fibrosis. Sci Adv *6*, eaba1972.

Hansen, C.G., Ng, Y.L., Lam, W.L., Plouffe, S.W., and Guan, K.L. (2015). The Hippo pathway effectors YAP and TAZ promote cell growth by modulating amino acid signaling to mTORC1. Cell Res *25*, 1299-1313.

Hu, J.K., Du, W., Shelton, S.J., Oldham, M.C., DiPersio, C.M., and Klein, O.D. (2017). An FAK-YAP-mTOR Signaling Axis Regulates Stem Cell-Based Tissue Renewal in Mice. Cell Stem Cell *21*, 91-106 e106.

Kobayashi, Y., Tata, A., Konkimalla, A., Katsura, H., Lee, R.F., Ou, J., Banovich, N.E., Kropski, J.A., and Tata, P.R. (2020). Persistence of a regeneration-associated, transitional alveolar epithelial cell state in pulmonary fibrosis. Nat Cell Biol *22*, 934-946.

Rawlins, E.L., Okubo, T., Xue, Y., Brass, D.M., Auten, R.L., Hasegawa, H., Wang, F., and Hogan, B.L. (2009). The role of Scgb1a1+ Clara cells in the long-term maintenance and repair of lung airway, but not alveolar, epithelium. Cell Stem Cell *4*, 525-534.

Strunz, M., Simon, L.M., Ansari, M., Kathiriya, J.J., Angelidis, I., Mayr, C.H., Tsidiridis, G., Lange, M., Mattner, L.F., Yee, M.*, et al.* (2020). Alveolar regeneration through a Krt8+ transitional stem cell state that persists in human lung fibrosis. Nat Commun *11*, 3559.

Tumaneng, K., Schlegelmilch, K., Russell, R.C., Yimlamai, D., Basnet, H., Mahadevan, N., Fitamant, J., Bardeesy, N., Camargo, F.D., and Guan, K.L. (2012). YAP mediates crosstalk between the Hippo and PI(3)K-TOR pathways by suppressing PTEN via miR-29. Nat Cell Biol *14*, 1322-1329.

Yoon, B.R., Oh, Y.J., Kang, S.W., Lee, E.B., and Lee, W.W. (2018). Role of SLC7A5 in Metabolic Reprogramming of Human Monocyte/Macrophage Immune Responses. Front Immunol *9*, 53.

Yue, M., Jiang, J., Gao, P., Liu, H., and Qing, G. (2017). Oncogenic MYC Activates a Feedforward Regulatory Loop Promoting Essential Amino Acid Metabolism and Tumorigenesis. Cell Rep *21*, 3819-3832.

Dear Dae-Sik, dear Joo-Hyeon,

Thank you for submitting your revised manuscript (EMBOJ-2021-109365R) to The EMBO Journal. Your amended study was sent back to the three referees for re-evaluation, and we have received comments from all of them, which I enclose below. As you will see, the referees stated that the issues raised have been adequately addressed and they are broadly now in favour of publication, pending a minor revision.

Thus, we are pleased to inform you that your manuscript has been accepted in principle for publication in The EMBO Journal.

Please consider the remaining point of referee #2 on the DAPT nomenclature carefully, and address it by adjusting the manuscript text where appropriate.

In addition, we need you to take care of a number of issues related to formatting and data representation as detailed below, which should be addressed at re-submission.

Please contact me at any time if you have additional questions related to below points.

As you might have noted on our web page, every paper at the EMBO Journal now includes a 'Synopsis', displayed on the html and freely accessible to all readers. The synopsis includes a 'model' figure as well as 2-5 one-short-sentence bullet points that summarize the article. I would appreciate if you could provide this figure and the bullet points.

Thank you for giving us the chance to consider your manuscript for The EMBO Journal. I look forward to your final revision.

Again, please contact me at any time if you need any help or have further questions.

Best regards,

Daniel

Daniel Klimmeck PhD
Senior Editor
The EMBO Journal

Formatting changes required for the revised version of the manuscript:

>> Adjust the title of the 'Competing Interests' section to 'Disclosure and Competing Interests Statement'.

>> Add a ToC on the first page of the Appendix .pdf file.

>> Add an http link for the GEO dataset in the Data Availability Section.

>> We appreciate your additional information provided on the data presented in Figures 4 and EV3. We do need you to provide Source Data - uncropped raw image files - for the microscopy images shown in Figures 4B, C,E, I, as well as Figure EV3. The sirius red-stained tissue section shown in Fig4E and EV3A appear similar, which needs clarification.

*****

Further information is available in our Guide For Authors: https://www.embopress.org/page/journal/14602075/authorguide

We realize that it is difficult to revise to a specific deadline. In the interest of protecting the conceptual advance provided by the work, we recommend a revision within 3 months (9th May 2022). Please discuss the revision progress ahead of this time with the editor if you require more time to complete the revisions. Use the link below to submit your revision:

https://emboj.msubmit.net/cgi-bin/main.plex

Referee #1:

The manuscript remains an impressive phenotypic characterisation with direct relevance to human lung inflammatory disease (BO / fibrosis). While I agree with Reviewer 2 that the mechanistic links between YAP, AA uptake, TORC1 activation and ATF4 are still a little unclear, this aspect is very much secondary to the main significance of the findings, which is implicating YAP activation in BO. Thus I recommend publication without further delay.

Referee #2:

Authors have addressed all comments. I commend the authors for addressing all comments. I recommend authors make following changes.

1. In response to my prior comment #9, author justified the use of DAPTs name instead of Basaloids. Although there are many similarities between ADI/PATS/DAPTs and basaloids, there do exist some differences. Therefore, it is appropriate that the authors use the name that was originally given by Habermann etal. 2020.

Referee #3:

The authors have adequately addressed all of my comments.

Response to the Reviewers' comments and Editor' suggestions

Referee #1:

The manuscript remains an impressive phenotypic characterisation with direct relevance to human lung inflammatory disease (BO / fibrosis). While I agree with Reviewer 2 that the mechanistic links between YAP, AA uptake, TORC1 activation and ATF4 are still a little unclear, this aspect is very much secondary to the main significance of the findings, which is implicating YAP activation in BO. Thus I recommend publication without further delay.

Response: We are grateful that Reviewer #1 appreciates our study's significant contribution, which implicates the role of YAP activity in BO.

Referee #2:

Authors have addressed all comments. I commend the authors for addressing all comments. I recommend authors make following changes.

1. In response to my prior comment #9, author justified the use of DAPTs name instead of Basaloids. Although there are many similarities between ADI/PATS/DAPTs and basaloids, there do exist some differences. Therefore, it is appropriate that the authors use the name that was originally given by Habermann etal. 2020.

Response: As suggested, we incorporated the terminology of basaloid in the study of human tissue analysis (Results, page 11, lines 7-9, 16)

Referee #3:

The authors have adequately addressed all of my comments.

Response: We are grateful that Reviewer #3 appreciated our improvements in addressing the concerns raised and the clarity in our revised manuscript.

Editor' suggestion:

Formatting changes required for the revised version of the manuscript:

>>Adjust the title of the 'Competing Interests' section to 'Disclosure and Competing Interests Statement'.

>> Add a ToC on the first page of the Appendix .pdf file.

>> Add an http link for the GEO dataset in the Data Availability Section.

Response: As suggested by Editor, (1) we newly incorporated the terminology of "basaloid" in the study of human tissue analysis. (2) We changed the title of the 'Competing Interests' section to 'Disclosure and Competing Interests Statement'. (3) added a ToC on the first page of the Appendix. (4) added an http link for the GEO dataset in the Data Availability Section.

>> We appreciate your additional information provided on the data presented in Figures 4 and EV3. We do need you to provide Source Data - uncropped raw image files - for the microscopy images shown in Figures 4B, C,E, I, as well as Figure EV3. The sirius red-stained tissue section shown in Fig4E and EV3A appear similar, which needs clarification.
Response:

We provided additional information on Figures 4 and EV3, and Source Data- uncropped raw image files (Figure 4B, C, E, I, EV3) and revised Figure 4 accordingly.

As pointed by editor, we found that the H & E staining of Fig4E and EV are the same because of the same control sample. Thanks for your important note. To avoid this confusion, we replaced this one with the same serial sectioned H & E staining of Fig4E. We also found that the Sirius red images for the Control+Vehicle group and dKO+AZD8055 group have been switched. We corrected this mistake.

1. Figure 4E: changed H&E images of Control and Lats1/2 dKO
2. Figure EV3G: switched Sirius red images of Control+Vehicle and dKO+AZD8055.

We are thankful for the reviewers' and editor's valuable comments, which helped us strengthen our study.

Dear Dr Dae-Sik Lim, dear Dr Joo-Hyeon Lee,

Thank you for submitting the revised version of your manuscript. I have now evaluated your amended manuscript and concluded that the remaining minor concerns have been sufficiently addressed.

Thus, I am pleased to inform you that your manuscript has been accepted for publication in the EMBO Journal.

Please note that it is EMBO Journal policy for the transcript of the editorial process (containing referee reports and your response letter) to be published as an online supplement to each paper. I would thus like to ask for your consent on keeping the additional reviewer figures included in this file.

Also, in case you might NOT want the transparent process file published at all, you will also need to inform us via email immediately. More information is available here: http://emboj.embopress.org/about#Transparent_Process

----

Please note that in order to be able to start the production process, our publisher will need and contact you regarding the following forms:

- PAGE CHARGE AUTHORISATION (For Articles and Resources)
http://onlinelibrary.wiley.com/journal/10.1002/(ISSN)1460-2075/homepage/tej_apc.pdf

- LICENCE TO PUBLISH (for non-Open Access)

Your article cannot be published until the publisher has received the appropriate signed license agreement. Once your article has been received by Wiley for production you will receive an email from Wiley's Author Services system, which will ask you to log in and will present them with the appropriate license for completion.

- LICENCE TO PUBLISH for OPEN ACCESS papers

Authors of accepted peer-reviewed original research articles may choose to pay a fee in order for their published article to be made freely accessible to all online immediately upon publication. The EMBO Open fee is fixed at $5,200 (+ VAT where applicable).

We offer two licenses for Open Access papers, CC-BY and CC-BY-NC-ND.
For more information on these licenses, please visit:
http://creativecommons.org/licenses/by-nc-nd/3.0/deed.en_US

- PAYMENT FOR OPEN ACCESS papers

You also need to complete our payment system for Open Access articles. Please follow this link and select EMBO Journal from the drop down list and then complete the payment process: https://authorservices.wiley.com/bauthor/onlineopen_order.asp

Should you be planning a Press Release on your article, please get in contact with embojournal@wiley.com as early as possible, in order to coordinate publication and release dates.

On a different note, I would like to alert you that EMBO Press is currently developing a new format for a video-synopsis of work published with us, which essentially is a short, author-generated film explaining the core findings in hand drawings, and, as we believe, can be very useful to increase visibility of the work. This has proven to offer a nice opportunity for exposure i.p. for the first author(s) of the study. Please see the following link for representative examples and their integration into the article web page:
https://www.embopress.org/video_synopses
https://www.embopress.org/doi/full/10.15252/embj.2019103932

Please let me know, should you be interested to engage in commissioning a similar video synopsis for your work. According operation instructions are available and intuitive.

If you have any questions, please do not hesitate to call or email the Editorial Office.

Thank you again for this contribution to The EMBO Journal and congratulations on a successful publication! Please consider us again in the future for your most exciting work.

Best regards,

Daniel Klimmeck

Daniel Klimmeck, PhD
Senior Editor
The EMBO Journal
EMBO
Postfach 1022-40
Meyerhofstrasse 1
D-69117 Heidelberg
contact@embojournal.org
Submit at: http://emboj.msubmit.net

EMBO PRESS

YOU MUST COMPLETE ALL CELLS WITH A PINK BACKGROUND ➔

PLEASE NOTE THAT THIS CHECKLIST WILL BE PUBLISHED ALONGSIDE YOUR PAPER

Corresponding Author Name: Dae-Sik Lim and Joo-Hyeon Lee
Journal Submitted to: EMBO Journal
Manuscript Number: EMBOJ-2021-109365

Reporting Checklist For Life Sciences Articles (Rev. June 2017)

This checklist is used to ensure good reporting standards and to improve the reproducibility of published results. These guidelines are consistent with the Principles and Guidelines for Reporting Preclinical Research issued by the NIH in 2014. Please follow the journal's authorship guidelines in preparing your manuscript.

A- Figures
1. Data
The data shown in figures should satisfy the following conditions:

- ➔ the data were obtained and processed according to the field's best practice and are presented to reflect the results of the experiments in an accurate and unbiased manner.
- ➔ figure panels include only data points, measurements or observations that can be compared to each other in a scientifically meaningful way.
- ➔ graphs include clearly labeled error bars for independent experiments and sample sizes. Unless justified, error bars should not be shown for technical replicates.
- ➔ if n< 5, the individual data points from each experiment should be plotted and any statistical test employed should be justified
- ➔ Source Data should be included to report the data underlying graphs. Please follow the guidelines set out in the author ship guidelines on Data Presentation.

2. Captions
Each figure caption should contain the following information, for each panel where they are relevant:

- ➔ a specification of the experimental system investigated (eg cell line, species name).
- ➔ the assay(s) and method(s) used to carry out the reported observations and measurements
- ➔ an explicit mention of the biological and chemical entity(ies) that are being measured.
- ➔ an explicit mention of the biological and chemical entity(ies) that are altered/varied/perturbed in a controlled manner.

- ➔ the exact sample size (n) for each experimental group/condition, given as a number, not a range;
- ➔ a description of the sample collection allowing the reader to understand whether the samples represent technical or biological replicates (including how many animals, litters, cultures, etc.).
- ➔ a statement of how many times the experiment shown was independently replicated in the laboratory.
- ➔ definitions of statistical methods and measures:
  - common tests, such as t-test (please specify whether paired vs. unpaired), simple χ^2 tests, Wilcoxon and Mann-Whitney tests, can be unambiguously identified by name only, but more complex techniques should be described in the methods section;
  - are tests one-sided or two-sided?
  - are there adjustments for multiple comparisons?
  - exact statistical test results, e.g., P values = x but not P values < x;
  - definition of 'center values' as median or average;
  - definition of error bars as s.d. or s.e.m.

Any descriptions too long for the figure legend should be included in the methods section and/or with the source data.

In the pink boxes below, please ensure that the answers to the following questions are reported in the manuscript itself. Every question should be answered. If the question is not relevant to your research, please write NA (non applicable). We encourage you to include a specific subsection in the methods section for statistics, reagents, animal models and human subjects.

USEFUL LINKS FOR COMPLETING THIS FORM

http://www.antibodypedia.com
http://1degreebio.org
http://www.equator-network.org/reporting-guidelines/improving-bioscience-research-repor
http://grants.nih.gov/grants/olaw/olaw.htm
http://www.mrc.ac.uk/Ourresearch/Ethicsresearchguidance/Useofanimals/index.htm
http://ClinicalTrials.gov
http://www.consort-statement.org
http://www.consort-statement.org/checklists/view/32-consort/66-title
http://www.equator-network.org/reporting-guidelines/reporting-recommendations-for-tumo
http://datadryad.org
http://figshare.com
http://www.ncbi.nlm.nih.gov/gap
http://www.ebi.ac.uk/ega
http://biomodels.net/
http://biomodels.net/miriam/
http://jjj.biochem.sun.ac.za
https://osp.od.nih.gov/biosafety-biosecurity-and-emerging-biotechnology/
http://www.selectagents.gov/

B- Statistics and general methods

Please fill out these boxes ➔ (Do not worry if you cannot see all your text once you press return)

1.a. How was the sample size chosen to ensure adequate power to detect a pre-specified effect size?	3-5 sampes were used per group
1.b. For animal studies, include a statement about sample size estimate even if no statistical methods were used.	3-5 mice were used for each group to get significant values using student's t-test
2. Describe inclusion/exclusion criteria if samples or animals were excluded from the analysis. Were the criteria pre-established?	No samples were excluded
3. Were any steps taken to minimize the effects of subjective bias when allocating animals/samples to treatment (e.g. randomization procedure)? If yes, please describe.	Mice within the sample genotypes were randomized for chemical treatments
For animal studies, include a statement about randomization even if no randomization was used.	Mice within the sample genotypes were randomized for chemical treatments
4.a. Were any steps taken to minimize the effects of subjective bias during group allocation or/and when assessing results (e.g. blinding of the investigator)? If yes please describe.	Samples were harvested and analyzed on the same days using the same methods to reduce subjective bias
4.b. For animal studies, include a statement about blinding even if no blinding was done	Quantifications of human and mouse data were performed blinded
5. For every figure, are statistical tests justified as appropriate?	Yes to the extent of our knowledge
Do the data meet the assumptions of the tests (e.g., normal distribution)? Describe any methods used to assess it.	Yes to the extent of our knowledge
Is there an estimate of variation within each group of data?	Yes to the extent of our knowledge

Is the variance similar between the groups that are being statistically compared?	Yes to the extent of our knowledge

C- Reagents

6. To show that antibodies were profiled for use in the system under study (assay and species), provide a citation, catalog number and/or clone number, supplementary information or reference to an antibody validation profile. e.g., Antibodypedia (see link list at top right), 1DegreeBio (see link list at top right).	Yes. This is described in Materials and Methods section
7. Identify the source of cell lines and report if they were recently authenticated (e.g., by STR profiling) and tested for mycoplasma contamination.	NA

* for all hyperlinks, please see the table at the top right of the document

D- Animal Models

8. Report species, strain, gender, age of animals and genetic modification status where applicable. Please detail housing and husbandry conditions and the source of animals.	Lats1fl/fl, Lats2fl/fl, Yapfl/fl, Tazfl/fl, Raptorfl/fl, Scgb1a1-CreERTM, R26-LSL-tdTomato, C57BL/6J male mice at the age of 4-5 weeks. All mice are housed and breeding under specific-pathogen-free (SPF) conditions at the KAIST animal facility. This is described in Matierals and Methods section.
9. For experiments involving live vertebrates, include a statement of compliance with ethical regulations and identify the committee(s) approving the experiments.	All mouse experiments were performed in accordance with recommendations approved by the Institutional Animal Care and Use Committee of KAIST.
10. We recommend consulting the ARRIVE guidelines (see link list at top right) (PLoS Biol. 8(6), e1000412, 2010) to ensure that other relevant aspects of animal studies are adequately reported. See author guidelines, under 'Reporting Guidelines'. See also: NIH (see link list at top right) and MRC (see link list at top right) recommendations. Please confirm compliance.	NA

E- Human Subjects

11. Identify the committee(s) approving the study protocol.	The institutional review board of Yonsei University Severance Hospital
12. Include a statement confirming that informed consent was obtained from all subjects and that the experiments conformed to the principles set out in the WMA Declaration of Helsinki and the Department of Health and Human Services Belmont Report.	Yes. This is described in Matierals and Methods section
13. For publication of patient photos, include a statement confirming that consent to publish was obtained.	NA
14. Report any restrictions on the availability (and/or on the use) of human data or samples.	NA
15. Report the clinical trial registration number (at ClinicalTrials.gov or equivalent), where applicable.	NA
16. For phase II and III randomized controlled trials, please refer to the CONSORT flow diagram (see link list at top right) and submit the CONSORT checklist (see link list at top right) with your submission. See author guidelines, under 'Reporting Guidelines'. Please confirm you have submitted this list.	NA
17. For tumor marker prognostic studies, we recommend that you follow the REMARK reporting guidelines (see link list at top right). See author guidelines, under 'Reporting Guidelines'. Please confirm you have followed these guidelines.	NA

F- Data Accessibility

18: Provide a "Data Availability" section at the end of the Materials & Methods, listing the accession codes for data generated in this study and deposited in a public database (e.g. RNA-Seq data: Gene Expression Omnibus GSE39462, Proteomics data: PRIDE PXD000208 etc.) Please refer to our author guidelines for 'Data Deposition'.<br><br>Data deposition in a public repository is mandatory for:<br>a. Protein, DNA and RNA sequences<br>b. Macromolecular structures<br>c. Crystallographic data for small molecules<br>d. Functional genomics data<br>e. Proteomics and molecular interactions	Yes, we deposited our single cell RNA-Seq data to GEO as #GSE178829.
19. Deposition is strongly recommended for any datasets that are central and integral to the study; please consider the journal's data policy. If no structured public repository exists for a given data type, we encourage the provision of datasets in the manuscript as a Supplementary Document (see author guidelines under 'Expanded View' or in unstructured repositories such as Dryad (see link list at top right) or Figshare (see link list at top right).	NA
20. Access to human clinical and genomic datasets should be provided with as few restrictions as possible while respecting ethical obligations to the patients and relevant medical and legal issues. If practically possible and compatible with the individual consent agreement used in the study, such data should be deposited in one of the major public access-controlled repositories such as dbGAP (see link list at top right) or EGA (see link list at top right).	NA
21. Computational models that are central and integral to a study should be shared without restrictions and provided in a machine-readable form. The relevant accession numbers or links should be provided. When possible, standardized format (SBML, CellML) should be used instead of scripts (e.g. MATLAB). Authors are strongly encouraged to follow the MIRIAM guidelines (see link list at top right) and deposit their model in a public database such as Biomodels (see link list at top right) or JWS Online (see link list at top right). If computer source code is provided with the paper, it should be deposited in a public repository or included in supplementary information.	NA

G- Dual use research of concern

22. Could your study fall under dual use research restrictions? Please check biosecurity documents (see link list at top right) and list of select agents and toxins (APHIS/CDC) (see link list at top right). According to our biosecurity guidelines, provide a statement only if it could.	NA
